# Heart rate recovery as a marker of post-exercise lipid metabolism following moderate- and vigorous-intensity exercise

**DOI:** 10.1007/s00421-026-06184-y

**Published:** 2026-03-07

**Authors:** Dirk Weber, Paola G. Ferrario, Achim Bub

**Affiliations:** 1 Institute of Sports and Sports Science, Karlsruhe Institute of Technology, Engler-Bunte-Ring 15, 76131 Karlsruhe, Germany; 2 Department of Physiology and Biochemistry of Nutrition, Max Rubner-Institute, Karlsruhe, Germany

**Keywords:** Exercise metabolomics, Heart rate recovery, Autonomic nervous system, Lipid metabolism, Acylcarnitines

## Abstract

**Purpose:**

Heart rate recovery (HRR) indicates post-exercise autonomic regulation and serves as a marker of cardiorespiratory fitness and mortality risk. Autonomic and metabolic recovery are both integral to post-exercise homeostasis, yet how HRR relates to metabolic recovery remains unclear.

**Methods:**

To address this gap, we analyzed data from a randomized crossover trial in 17 healthy, physically active young men, each performing 30 min of both moderate- and vigorous-intensity ergometer cycling. Plasma samples collected before exercise and at multiple recovery time points were analyzed using UPLC–MS/MS–based untargeted metabolomics, covering more than 1000 metabolites. HRR was calculated using a monoexponential decay model, and associations were examined using linear mixed models.

**Results:**

Individuals with faster HRR exhibited significantly lower post-exercise levels across a range of lipid metabolites, particularly acylcarnitines. These associations were stronger for HRR than VO₂peak and were statistically significant only in the later recovery period (90–180 min post-exercise), exclusively following vigorous-intensity exercise. Our findings suggest that HRR reflects post-exercise lipid metabolism under conditions of high metabolic demand. The observed metabolite patterns are indicative of differences in β-oxidation, lipid accumulation, reliance on ω-oxidation, and mitochondrial turnover, and are consistent with more efficient post-exercise lipid metabolism.

**Conclusion:**

HRR may provide a simple marker of metabolic or cardiorespiratory fitness and could be relevant for monitoring exercise responses and assessing cardiometabolic health. However, confirmation in larger and more diverse cohorts is required.

**Clinical trials register:**

The trial was registered on October 5, 2017, at the German Clinical Trials Register under the registration number DRKS00009743 (Universal Trial Number of WHO: U1111-1200–2530).

**Graphical abstract:**

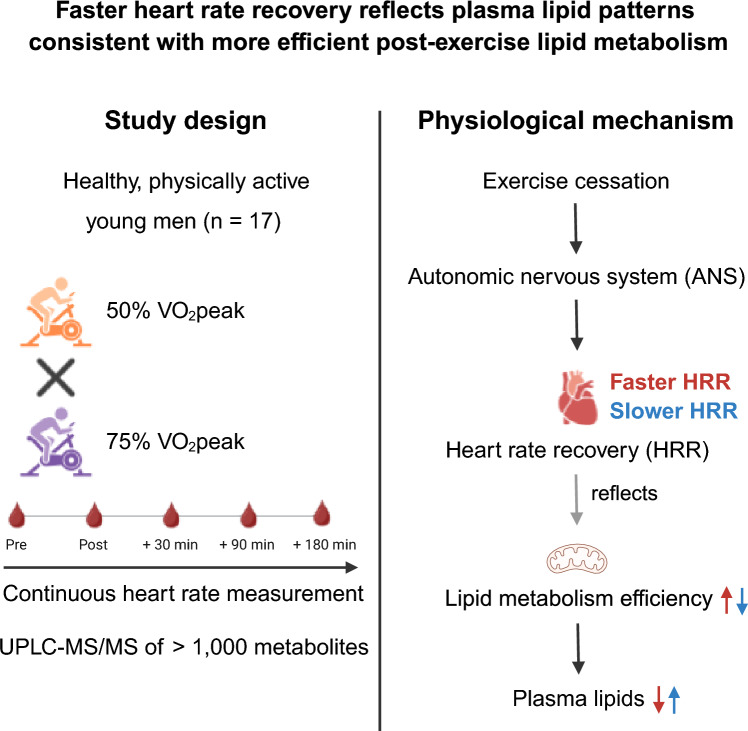

**Supplementary Information:**

The online version contains supplementary material available at 10.1007/s00421-026-06184-y.

## Introduction

Post-exercise heart rate recovery (HRR) refers to the decline in heart rate following exercise cessation and is recognized as a non-invasive indicator of autonomic nervous system regulation during recovery (Borresen and Lambert 2008). HRR is driven by rapid parasympathetic reactivation during the immediate phase of recovery, together with more gradual sympathetic withdrawal (Pierpont and Voth 2004). HRR is typically quantified as the difference between heart rate at exercise cessation and heart rate measured at fixed time points during the first minutes of recovery (e.g., 60 or 120 s) (Buchheit 2014; Daanen et al. 2012). Slower HRR has consistently been reported as an independent predictor of cardiovascular and all-cause mortality (Cole et al. 1999; Peçanha et al. 2014), whereas faster HRR is frequently linked to better cardiorespiratory fitness (Daanen et al. 2012; Jolly et al. 2011). Accordingly, HRR serves both as a prognostic indicator of mortality in clinical settings and as a non-invasive marker of training status and physiological adaptation in sports contexts (Bosquet et al. 2008).

Untargeted metabolomics provides a comprehensive approach to investigate metabolic responses to exercise and subsequent recovery (Khoramipour et al. 2022). Circulating metabolites provide an indirect proxy of systemic metabolism, but not all metabolites enter the bloodstream and their appearance may be time- and tissue-dependent. Exercise metabolomics studies have demonstrated that exercise induces pronounced systemic alterations across multiple metabolic pathways, particularly those related to energy metabolism. These alterations are characterized by increases in tricarboxylic acid (TCA) cycle intermediates, glycolytic end-products, and intermediates of lipid metabolism, such as acylcarnitines (Kelly et al., 2020; Schranner et al. 2020). After exercise cessation, most metabolites gradually return toward baseline, whereas certain lipid metabolites remain elevated for a longer duration, reflecting sustained fatty acid β-oxidation as part of metabolic homeostatic regulation (Magkos et al. 2009). These lipid responses illustrate that metabolic recovery can extend well beyond the normalization of cardiorespiratory variables (Magkos et al. 2009). Interactions between autonomic and metabolic regulation have been demonstrated, with autonomic activity influencing substrate utilization (Nonogaki 2000; Wangler et al. 2026) and metabolites modulating sympathetic activity via the muscle metaboreflex (Boushel 2010; Michelini et al. 2015). Despite these findings, it remains unclear whether HRR, as an indicator of autonomic regulation, is linked to metabolic recovery after exercise.

Therefore, this study aimed to investigate whether HRR reflects post-exercise metabolic recovery in healthy, physically active young men. To address this, we analyzed data from a randomized crossover trial in which 17 participants completed 30 min of ergometer exercise at moderate and vigorous intensities. As metabolic responses to exercise depend on exercise intensity (Weber et al. 2025), moderate- and vigorous-intensity conditions were included to explore whether the relationship between heart rate recovery and post-exercise metabolic changes differs across exercise intensities. Plasma samples collected before and during recovery were analyzed by untargeted metabolomics to characterize metabolite recovery trajectories. We hypothesized that HRR reflects the recovery dynamics of metabolites and metabolic pathways involved in energy metabolism. As both HRR and metabolic recovery capture aspects of post-exercise homeostasis, examining their relationship may provide insights into the interplay between autonomic and metabolic regulation. Establishing such a link could enable the use of HRR as a simple, non-invasive marker of metabolic recovery, with potential applications in exercise monitoring and clinical assessment of metabolic health.

## Methods

### Study design

This study was conducted in accordance with the CONSORT extension for reporting randomized crossover trials (Dwan et al. 2019). The study design, participant characteristics, and measurement procedures have been described in detail elsewhere (Kistner et al. 2023; Weber et al. 2025). In brief, 17 healthy, physically active men aged 18–30 years participated in the trial. Inclusion and exclusion criteria are summarized in Supplementary Table 1. Sample size was calculated based on the study’s pre-specified primary outcome. Analyses of metabolomics and HRR were conducted as secondary analyses.

Anthropometric measures were taken before study onset and included body weight and height (Seca 285, Hamburg, Germany), from which body mass index (BMI) was calculated. Body composition was assessed by bioelectrical impedance analysis (BIA Nutriguard MS, Data Input, Pöcking, Germany) to estimate lean body mass, fat mass, and percent body fat. Resting heart rate (HRrest) as well as systolic and diastolic blood pressure were measured after participants had been seated at rest for at least 5 min (Boso Carat Professional, Bosch & Sohn, Jungingen, Germany).

The trial was conducted at the Max Rubner-Institute (Karlsruhe, Germany) between October 2017 and May 2018. It consisted of two exercise interventions at different intensities: continuous moderate-intensity exercise (CME) and continuous vigorous-intensity exercise (CVE). The study followed a randomized two-period crossover design, with each participant completing both interventions in a randomized order. As sustained metabolic alterations are not expected following a single exercise bout (Kelly et al., 2020), a washout period of at least one week was considered sufficient to minimize potential carry-over effects. To individualize exercise intensity, participants performed a cardiopulmonary exercise test on a bicycle ergometer (SRM Sport High Performance, SRM, Jülich, Germany) during a familiarization session before the trial. Peak oxygen uptake (VO₂peak) was determined and used to calculate workloads, set at 50% (CME) and 75% (CVE) of each participant’s power output at VO₂peak. Participants were instructed to refrain from strenuous physical activity on the day before each trial and followed a standardized diet on the trial days and preceding days. Details on energy intake, macronutrient composition, and water consumption are provided in Supplementary Table 2.

**Fig. 1:**
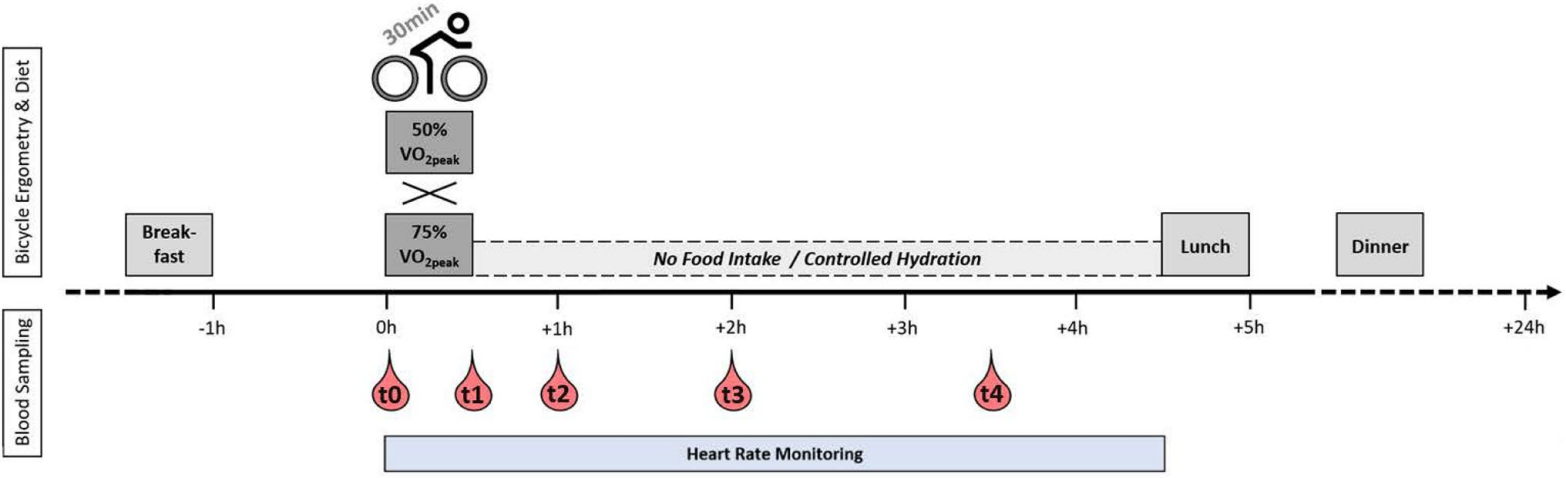
Study design of the randomized crossover trial. Seventeen healthy, physically active young men completed a 30-min continuous moderate exercise (CME) trial and a 30-min continuous vigorous exercise (CVE) trial in randomized order. On both trial days, participants received standardized breakfast and lunch meals. Venous blood samples were collected at baseline before exercise (t0), immediately after exercise (t1), and during recovery at 30 min (t2), 90 min (t3), and 180 min (t4) post exercise

On each study day, participants arrived at the study center at 7:30 a.m. after an overnight fast. Study procedures were explained and a venous catheter was inserted for blood sampling. 90 min before the experimental trial, a standardized breakfast was served, after which the participants remained seated at rest for 1 h. Both exercise sessions were carried out between 9:00 a.m. and 10:00 a.m. The testing room was maintained under standardized conditions in line with the Practice Guidelines for Exercise Testing (Wonisch et al. 2014). Immediately before exercise, the first blood sample (t0) was collected. Each session began with a 2 min warm-up at 50 W (CME) or 100 W (CVE), followed by 30 min of continuous cycling at 50% (CME) or 75% (CVE) of VO₂peak. A continuous exercise bout of 30 min is generally considered sufficient to induce substantial metabolic responses, as this duration has been widely applied in previous studies (Brugnara et al. 2012; Danaher et al. 2015) and was shown to induce pronounced metabolic changes in a previous analysis of this dataset (Weber et al. 2025). These studies have demonstrated alterations in lipid metabolism, glycolysis, and TCA cycle intermediates, indicating that this duration is sufficient to induce substantial metabolic perturbations. After completion, participants completed an active recovery period at 50 W for 3 min (CME) or at 100 W for 1 min followed by 2 min at 50 W (CVE). Further blood samples were obtained immediately after exercise (t1), and at 30 (t2), 90 (t3), and 180 (t4) minutes post exercise. At 240 min post exercise, participants received a standardized lunch. Between breakfast and lunch, participants remained sedentary and consumed only water, which was recorded throughout the trial. Figure 1 illustrates the study protocol.

### Heart rate recovery (HRR) assessment

Heart rate (HR) was continuously monitored from the beginning of the experimental trial until lunch (Polar H7, Polar, Kempele, Finland). Values were recorded at fixed intervals of 10 or 15 s, which varied across exercise sessions but were consistent within each session. As exercise cessation was recorded only to the nearest full minute, it did not align with available HR sampling time points. Therefore, exercise cessation was defined as the first HR value after the recorded end of exercise for which at least ten subsequent values were each ≥ 3 bpm lower than that value. HRR was modeled over 10 min post exercise to account for the slower decline during active recovery and because most participants did not achieve full recovery within 5 min.

In some exercise sessions, a substantial number of HR values were missing, due to technical recording problems. Specifically, three exercise sessions (two CME and one CVE) exhibited extensive missing HR data and were therefore excluded. In the remaining sessions, HR values were excluded as implausible if they were at least 10 bpm lower than any two subsequent values or at least 10 bpm higher than any two preceding values. This criterion was applied to identify isolated artifacts while preserving the underlying heart rate recovery trajectory. To reduce measurement noise, HR data were smoothed using a cubic smoothing spline (R package *stats*, function smooth.spline; spar = 0.8).

HRR is best described by a first-order exponential decay curve (Buchheit 2014; Pierpont & Voth 2004). Therefore, HRR curves were fitted using a monoexponential decay model via nonlinear least squares, with the Levenberg–Marquardt algorithm (R package *minpack.lm*, function nlsLM):

HR(t) = HRrest + (HRpeak − HRrest) ⋅ e^−t/τ^

HRrest is the resting heart rate, HRpeak the heart rate at exercise cessation, and τ is the time constant (in seconds) quantifying the rate of recovery. The model-derived HRrest was constrained to a physiologically meaningful range of 45–100 bpm, whereas the other model parameters (HRpeak and τ) were left unconstrained. With starting values based on HRrest and the immediate post-exercise heart rate (HRpeak), the model was fitted for each participant and exercise session. HRR was defined as the time constant (τ) of the model, with lower τ values indicating faster recovery. Model fit was evaluated using residual standard error (RSE), mean absolute error (MAE), and by comparing observed and predicted HR values as well as their residuals through visual inspection. Model parameters and fit metrics are presented in Supplementary Table 3, and the corresponding fit visualizations are provided in Supplementary Fig. 1. Most models showed good fit based on these criteria. However, two CVE sessions demonstrated poor fit quality (RSE > 4.0 and MAE > 3.5) and were excluded from further analyses. Importantly, this exclusion applied only to these specific sessions and not to the corresponding participants, whose remaining sessions were retained. A bi-exponential decay model was also tested but failed to produce stable fits, and simpler approaches (e.g., HR₆₀, logarithmic method) were not compatible with the study design.

### Metabolomics analysis

The metabolomics data processing has been described in detail elsewhere (Weber et al. 2025). Briefly, blood samples were centrifuged at 2500×*g* for 10 min at 4 °C to obtain plasma, which was subsequently aliquoted into cryovials and stored in the gas phase of liquid nitrogen at − 196 °C. Plasma samples were sent to Metabolon (Metabolon Inc., North Carolina, USA) for untargeted metabolomics profiling and analyzed using multiple UPLC-MS/MS methods. In total, 1262 metabolites were detected. All identified metabolites were assigned to super-pathways and sub-pathways according to Metabolon’s classification system (Supplementary Table 4). We obtained the raw peak area data from Metabolon and excluded all metabolites with more than 20% missing values. Missing values in the resulting dataset of 1025 metabolites were then imputed using a random forest imputation approach (R package *missForest*, function missForest).

### Statistical analyses

Linear mixed models were used to investigate the relationship between HRR and post-exercise metabolic recovery (R package *lme4*, function lmer). Each metabolite was analyzed in a separate model as the dependent variable. The base model had the structure *Metabolite* ~ *HRR* × *Time* + *Intensity* + *Metbaseline* + *(1 | Participant)*. It included HRR, Time, and their interaction (HRR × Time) as fixed effects, with baseline metabolite levels at t0 (Metbaseline) included to account for interindividual differences in starting values, and a random intercept for Participant.

Several potential covariates were evaluated in 25 randomly selected metabolites by extending the base model (base + covariate) and comparing model fit (R package *stats*, function anova). Covariates included Sequence and Period of intervention, HRinterval (10 vs. 15 s sampling), and participant characteristics with high coefficients of variation (fat mass %, HRrest, power at lactate threshold, power at individual anaerobic threshold). We also evaluated fitness indicators (VO₂peak, HRmax, Pmax) to account for differences in aerobic capacity. Covariates were retained if they improved model fit (ΔAIC < –2) in > 50% of tested models. A ΔAIC < –2 indicates a substantially better trade-off between goodness of fit and model complexity and is widely used as a criterion for meaningful improvement. All test results for the tested models are detailed in Supplementary Table 5. As no covariate met this criterion, all analyses were conducted with the base model.

The association between HRR and metabolite recovery trajectories was assessed via the HRR × Time interaction, using the immediate post-exercise time point (t1) as the reference. The immediate post-exercise time point was used as the reference to specifically examine post-exercise metabolite trajectories. Prior to analysis, HRR was standardized (z-score) to allow comparability of effect sizes. Model estimates were calculated on log₂-transformed metabolite levels and back-transformed to fold changes for interpretability. Normality of residuals was assessed by Q–Q plots. All p-values were corrected for multiple testing using the Benjamini–Hochberg approach, resulting in q-values. Statistical significance was defined as q < 0.05. Pathway enrichment analyses were conducted using Fisher’s exact test with the same adjustment for multiple testing. This approach compares the number of significant metabolites within each pathway to the total number of metabolites assigned to that pathway, testing whether the observed proportion exceeds what would be expected by chance. Differences in energy intake, nutrient intake, and water consumption were tested using paired Wilcoxon signed-rank tests. All analyses and figures were generated in R (version 4.5.1).

## Results

### Participant characteristics

The 17 healthy men had a mean BMI of 22.8 ± 1.9 kg/m2 and a mean resting heart rate (HRrest) of 63 ± 16 bpm. In preliminary exercise testing, participants reached a mean maximal heart rate (HRmax) of 191 ± 8 bpm and a mean VO₂peak of 56.6 ± 6.3 mL·kg⁻1·min⁻1. During the exercise trials, participants reached a mean peak HR of 132 ± 13 bpm in the CME trial and 169 ± 13 bpm in the CVE trial. Participant characteristics and exercise parameters are summarized in Table 1.

**Table 1:** Descriptive characteristics of participants (n = 17) and exercise parameters

Participant characteristics	Mean ± SD	Minimum	Maximum
Age (years)	24.8 ± 2.7	18.7	30.3
Body height (cm)	183 ± 6	175	191
Body weight (kg)	75.8 ± 7.1	67.0	90.8
Body mass index (kg m^−2^)	22.8 ± 1.9	20.2	25.9
Lean body mass (kg)	64.4 ± 6.1	56.8	75.4
Fat mass (%)	14.9 ± 4.6	7.4	24.8
Systolic blood pressure (mmHg)	125 ± 10	108	146
Diastolic blood pressure (mmHg)	76 ± 7	66	87
HRrest (bpm)	63 ± 16	49	112
*Preliminary exercise testing*			
VO_2_peak (mL kg^−1^ min^−1^)	56.6 ± 6.3	45.0	68.0
Pmax (W)	340.9 ± 43.3	273.7	450.0
PLT (W)	164.6 ± 31.1	113.3	238.0
PIAT (W)	215.6 ± 35.0	149.5	289.6
HRmax (bpm)	191 ± 8	173	204
*Exercise interventions*			
*CME trial*			
P50% (W)	129.4 ± 20.4	77.0	156.0
Peak HR (bpm)	132 ± 13	109	153
*CVE trial*			
P75% (W)	224.4 ± 31.2	163.0	270.0
Peak HR (bpm)	169 ± 13	148	189

*SD* standard deviation, *HRrest* resting heart rate, *VO*_*2*_*peak* peak oxygen uptake; Pmax, maximum power, *PLT* power at the lactate threshold; PIAT, power at the individual anaerobic threshold, *HRmax* maximal heart rate, *P50%* power at 50% VO_2_peak, *P75%* power at 75% VO_2_peak, *Peak HR* highest HR during the exercise trial

### Associations between HRR and metabolic recovery

**Fig. 2:**
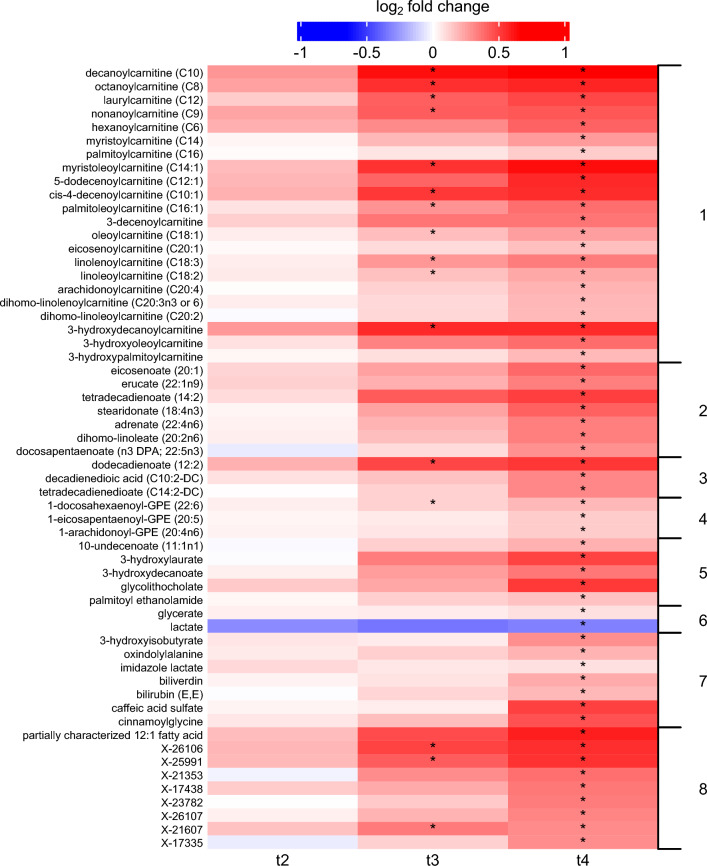
Heatmap of associations between HRR and metabolite levels at post-exercise time points. Rows correspond to individual metabolites and columns to recovery time points. Colors represent the direction and strength of associations, with red indicating that individuals with faster HRR showed lower levels relative to the immediate post-exercise value, and blue indicating that individuals with faster HRR showed higher levels. Only metabolites with at least one significant association (q < 0.05) are displayed. Metabolites are ordered hierarchically by super-pathway, sub-pathway, and effect estimate. Row groups (1–8) on the right indicate biochemical classes: 1 = acylcarnitines; 2 = long-chain monounsaturated and polyunsaturated fatty acids; 3 = fatty acid dicarboxylates; 4 = lysophospholipids; 5 = other lipids; 6 = glycolysis-related metabolites; 7 = other metabolites; 8 = partially characterized or uncharacterized compounds

Separate linear mixed models were used to analyze associations between HRR and metabolite recovery trajectories, with the HRR × Time interaction serving as the primary effect of interest. To visualize model results, a heatmap (R package *ComplexHeatmap* (Gu et al. 2016)) of all significant metabolites was generated (Fig. 2). No metabolites were significantly associated with HRR 30 min post exercise, whereas 16 were significant at 90 min and 58 at 180 min after exercise. All metabolites significant at 90 min remained significant at 180 min, with a consistent direction of association across time points. Detailed model results for all associations between HRR and metabolites across post-exercise time points are provided in Supplementary Table 6. Positive estimates indicate that faster HRR (lower τ) was associated with lower post-exercise levels relative to the immediate post-exercise value (t1), whereas negative estimates indicate higher levels. For all significant metabolites, faster HRR was associated with lower post-exercise levels, with lactate being the only exception, showing higher post-exercise levels. Effect sizes ranged from 0.79-fold to 1.58-fold per SD in HRR. All metabolite values represent semi-quantitative peak areas rather than absolute concentrations.

**Fig. 3:**
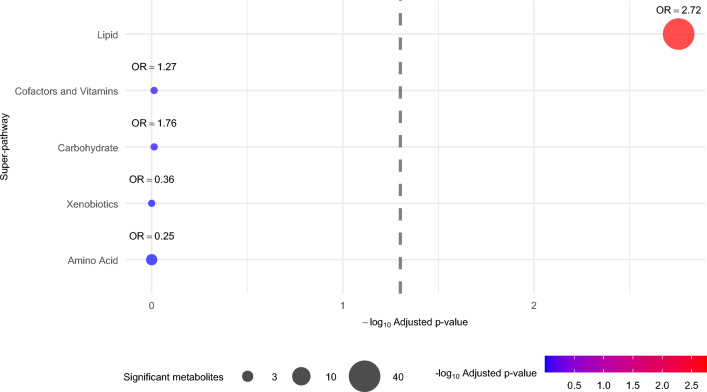
Super-pathway enrichment analysis. Fisher’s exact test was used to identify significantly enriched pathways based on Metabolon’s super-pathway classification. P-values were adjusted for multiple testing using the Benjamini–Hochberg procedure. The grey dashed vertical line marks the significance threshold at an adjusted p-value of 0.05, with pathways to the right considered significantly enriched. The color scale represents the -log₁₀ of the adjusted p-value, ranging from blue (lower values) to red (higher values). Odds ratios (OR) indicate whether a pathway is overrepresented (OR > 1) or underrepresented (OR < 1) among significant metabolites. Only pathways with at least two significant metabolites are displayed

To determine whether certain metabolic pathways were overrepresented among the metabolites significantly associated with HRR, we performed a super-pathway enrichment analysis (Fig. 3). The analysis was based on Metabolon’s pathway classification and focused on time point t4, which showed the highest number of significant associations. Among all tested super-pathways, only the lipid pathway was significantly overrepresented, comprising 40 of the 58 significant metabolites. As the Metabolon sub-pathway classification groups lipid metabolites in a heterogeneous manner (e.g., splitting acylcarnitines into multiple arbitrary subcategories), we did not conduct a sub-pathway enrichment analysis. Significant lipid metabolites predominantly included acylcarnitines (n = 22), long-chain monounsaturated and polyunsaturated fatty acids (n = 7), fatty acid dicarboxylates (n = 3), and lysophospholipids (n = 3).

### Relationship between HRR and lipid metabolites

**Fig. 4:**
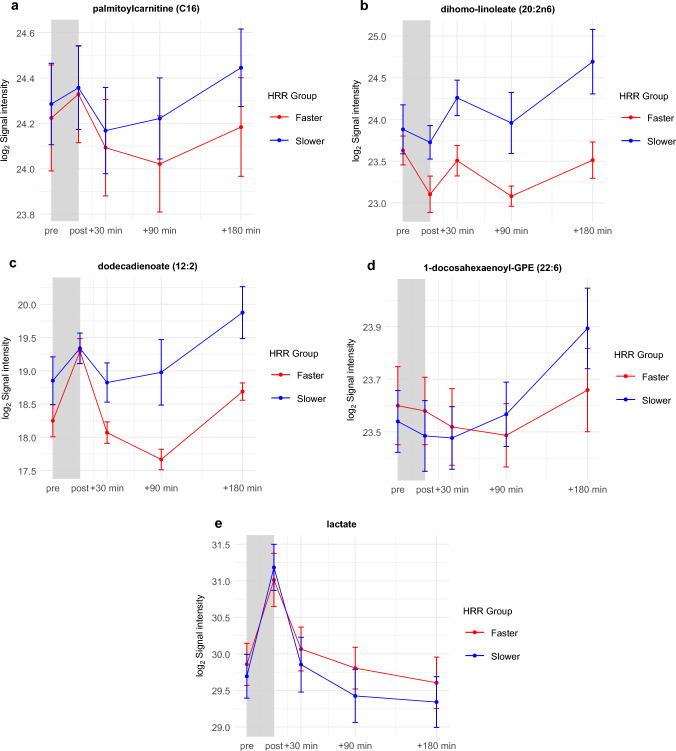
Line plots of selected metabolites from the continuous vigorous exercise (CVE) trial. Each line plot is plotted on an independent y-axis scale to emphasize within-metabolite dynamics; absolute values are therefore not directly comparable across metabolites. Mean log₂-transformed signal intensities with their standard error of the mean (SEM) are shown across participants (n = 14 (faster HRR: n = 7; slower HRR: n = 7)) over time. Participants were stratified by heart rate recovery (HRR) relative to the median (slower HRR: blue; faster HRR: red). The x-axis displays sampling time points relative to the exercise intervention, and the y-axis indicates the mean log₂ signal intensity. The grey area marks the duration of the exercise intervention

To visualize differences in metabolic recovery between individuals with faster and slower HRR, participants were stratified by the median HRR into two groups (faster HRR: n = 7; slower HRR: n = 7), and mean metabolite levels from the CVE trial (n = 14) were plotted for each group (Fig. 4a–e). To illustrate patterns across lipid categories, we selected one representative metabolite per group: Palmitoylcarnitine (C16) (saturated long-chain acylcarnitine), dihomo-linoleate (20:2n6) (long-chain polyunsaturated fatty acid), dodecadienoate (C12:2) (fatty acid dicarboxylate), and 1-docosahexaenoyl-GPE (22:6) (lysophospholipid), alongside lactate. Across lipid metabolites, levels decreased immediately after exercise and then increased again from 90 to 180 min but remained consistently lower in the faster HRR group throughout recovery. These trajectories reflect greater post-exercise decreases in the faster HRR group, where metabolite levels declined more markedly and dropped below baseline. Minor differences between the faster and slower HRR groups were observed in both pre-exercise and immediate post-exercise values, most notably for dodecadienoate (C12:2), but the main divergence between groups emerged during recovery. In contrast, lactate concentrations decreased throughout recovery, with a more pronounced decline in individuals with slower HRR.

### Robustness and specificity analyses

To assess whether the observed associations differed by exercise intensity, we repeated the main analysis separately for each intensity, focusing on t4. The analysis revealed that after vigorous exercise, 115 metabolites were significantly associated with HRR, compared with 58 when both intensities were combined, whereas no significant associations were observed after moderate exercise. Most significant metabolites in the vigorous-intensity analysis were lipids and acylcarnitines, consistent with the main analysis.

As HRR has frequently been linked to cardiorespiratory fitness, we examined its associations in relation to VO₂peak, a widely used measure of fitness. We quantified the correlation between HRR and VO₂peak using Spearman’s rank correlation and found that they were weakly but significantly correlated (ρ =  − 0.38, p = 2.6 × 10⁻5). Additionally, we included VO₂peak in the main model, which did not substantially affect the associations. When HRR was replaced by VO₂peak in the main model, the interaction between VO₂peak and Time at t4 was significant for 21 metabolites, most of which were acylcarnitines. However, compared with HRR, both the number of significant associations and their effect sizes were lower for VO₂peak. When both HRR and VO₂peak were modeled together with their interaction with Time at t4, the number of significant associations for HRR was slightly reduced, whereas no associations were detected for VO₂peak.

As some lipids, such as dodecadienoate (12:2), showed a stronger acute response to the exercise intervention in participants with faster HRR, we considered whether this might have influenced the observed associations between HRR and post-exercise lipid metabolism in the main analysis. To test this, we excluded t1 from the analysis so that t2 served as the reference, allowing us to evaluate recovery patterns independently of the acute response. The results were highly consistent with those of the main analysis. Additionally, we tested whether the acute metabolic response (t0 to t1) was associated with HRR using the main model limited to these time points, and without baseline adjustment. None of the metabolites were significant after correction for multiple testing.

Because acylcarnitines showed the most consistent associations, we grouped them based on biochemical characteristics to enable clearer functional interpretation. Specifically, we summed acylcarnitines by chain length (short-, medium-, and long-chain), degree of unsaturation (saturated, monounsaturated, polyunsaturated), hydroxylation status (hydroxy, non-hydroxy), and biochemical origin (lipid-derived, non-lipid-derived). Metabolites could belong to more than one subgroup depending on their properties. The summed subgroup values (log₂-transformed) were then analyzed using the same linear mixed models as for individual metabolites, with p-values adjusted for multiple testing using the Benjamini–Hochberg procedure. All subgroups showed significant associations with the HRR × Time interaction at t4, with faster HRR being associated with lower levels, except for the non–lipid-derived acylcarnitines, which did not show significant associations (Supplementary Table 7).

To test whether results were influenced by single participants, we conducted leave-one-participant-out analyses, repeating the models while excluding each participant in turn. Results were highly consistent across participants, and none of the key metabolites of interest lost significance in any iteration. Additionally, we tested various HR smoothing algorithms with different strengths and found that these had minimal impact on our results.

## Discussion

This study investigated whether heart rate recovery (HRR) reflects post-exercise metabolic recovery in healthy, physically active young men. HRR was significantly associated with numerous lipid metabolites, including acylcarnitines, long-chain monounsaturated and polyunsaturated fatty acids, fatty acid dicarboxylates, and lysophospholipids. Individuals with faster HRR consistently exhibited lower post-exercise levels of these metabolites, whereas lactate was the only metabolite to show higher levels. These associations were more pronounced for HRR than for VO₂peak and were restricted to the later recovery period (90–180 min post-exercise) and to vigorous-intensity exercise.

Associations between HRR and lipid metabolites emerged only in the later recovery period, a phase characterized in our data by a marked increase in circulating lipid metabolites. As exercise constitutes a substantial metabolic challenge, differences in lipid metabolism would be expected to manifest primarily during the exercise bout itself. However, no associations emerged immediately post-exercise, likely because standardizing workload to VO₂peak minimized interindividual variation in metabolic strain. In contrast, the recovery period cannot be standardized to fitness, allowing differences in metabolic efficiency to emerge gradually and become apparent in the later recovery period. This timing aligns with established recovery dynamics, as oxygen consumption remains elevated during post-exercise recovery (Børsheim and Bahr 2003), and energy provision increasingly shifts toward fatty acid oxidation to spare glucose for glycogen resynthesis (Kimber et al. 2003; Magkos et al. 2009). With the progressive upregulation of fatty acid oxidation, interindividual differences in lipid metabolism become evident, which may help explain why associations between HRR and lipid metabolites are observed specifically during the later recovery period. Notably, the observed associations occurred exclusively after vigorous exercise. This aligns with evidence that the post-exercise upregulation of fatty acid oxidation depends on exercise intensity, due to greater glycogen depletion at higher intensities (Bahr et al. 1991; Børsheim and Bahr 2003). Together, these observations suggest that HRR reflects interindividual differences in lipid metabolism only when metabolic demand is sufficiently high and when variation in metabolic strain is not minimized by workload standardization.

Acylcarnitines represented by far the largest group of metabolites associated with HRR, and their accumulation reflects both metabolic flux and incomplete β-oxidation (Koves et al. 2008). Therefore, lower levels in individuals with faster HRR are consistent with reduced incomplete β-oxidation and likely reflect a higher β-oxidation flux during recovery. This interpretation is supported by the concomitant behavior of long-chain monounsaturated and polyunsaturated fatty acids. As these are less favored substrates for mitochondrial fatty acid oxidation (Poirier et al. 2006), lower levels may suggest diminished upstream accumulation. A similar pattern was observed for fatty acid dicarboxylates. When mitochondrial β-oxidation is insufficient, ω-oxidation is upregulated to compensate, producing these dicarboxylated species (Ranea-Robles and Houten 2023). Their lower abundance in individuals with faster HRR therefore suggests reduced reliance on this auxiliary pathway, consistent with more efficient mitochondrial β-oxidation. However, as long-chain polyunsaturated fatty acids and fatty acid dicarboxylates are mainly metabolized via peroxisomal β-oxidation (Poirier et al. 2006; Ranea-Robles & Houten 2023), it remains unclear whether these changes primarily reflect mitochondrial β-oxidation or additionally involve peroxisomal β-oxidation. As mitochondrial β-oxidation is particularly susceptible to metabolic bottlenecks (Koves et al. 2023), interindividual differences in its efficiency may best capture the variability reflected by HRR. Other pathways, such as the TCA cycle, receive only limited substrate input when glycolysis is downregulated and therefore show little or no accumulation of intermediates that could be reflected by HRR. However, because we did not directly measure metabolic flux dynamics, any conclusions regarding differences in the efficiency of specific pathways, such as β-oxidation, must be considered indirect.

In line with the concept of differences in mitochondrial lipid metabolism efficiency, individuals with faster HRR showed lower post-exercise levels of lysophospholipids, which reflect membrane deacylation and turnover (Patton-Vogt and de Kroon 2020). The observed lysophospholipids belong to the lysophosphatidylethanolamine subclass, which is enriched in mitochondrial membranes (Calzada et al. 2019), and lower levels therefore likely indicate less mitochondrial membrane stress. Although exercise stimulates mitochondrial turnover, acute mitophagy is greater in untrained muscle due to higher mitochondrial damage (Chen et al. 2018). Lower lysophospholipid levels in individuals with faster HRR may therefore reflect reduced acute mitochondrial membrane remodeling, consistent with more resilient mitochondrial structures.

**Fig. 5:**
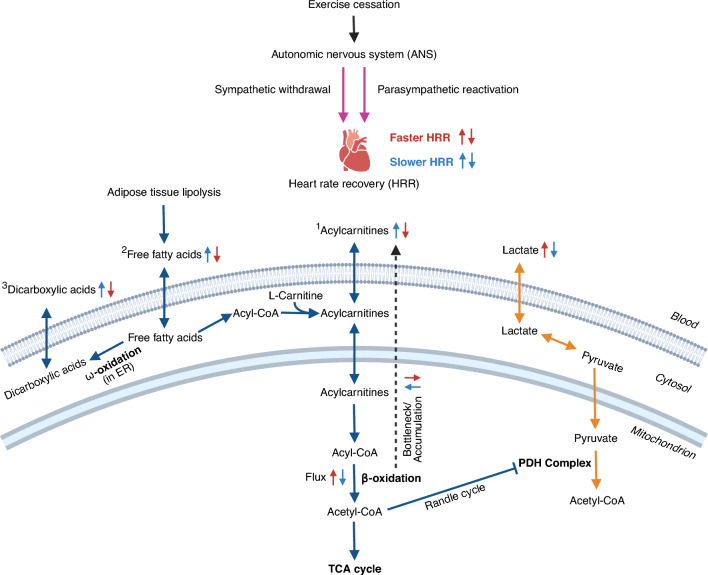
Proposed mechanistic model linking HRR to post-exercise metabolic recovery. Exercise cessation induces autonomic nervous system (ANS) changes, with sympathetic withdrawal and parasympathetic reactivation driving HRR. Slower HRR (blue) is associated with reduced β-oxidation flux, resulting in spillover of acylcarnitines, and secondary increases in free fatty acids, ω-oxidation, and fatty acid dicarboxylates. Faster HRR (red) is characterized by greater β-oxidation flux, reduced spillover, and higher acetyl-CoA production, which contributes to inhibition of the pyruvate dehydrogenase (PDH) complex and to upstream lactate accumulation, consistent with the Randle cycle. Numbers (1–3) indicate representative example metabolites for each group, as listed below: (1) acylcarnitines such as palmitoylcarnitine (C16:0), myristoleoylcarnitine (C14:1), and linoleoylcarnitine (C18:2); (2) free fatty acids such as dihomo-linoleate (20:2n6), docosapentaenoate (n3 DPA; 22:5n3), and eicosenoate (20:1); and (3) fatty acid dicarboxylates such as dodecadienoate (12:2), decadienedioic acid (C10:2-DC), and tetradecadienedioate (C14:2-DC)

The behavior of lactate, although initially counterintuitive, is consistent with our mechanistic interpretation. Increased β-oxidation flux elevates acetyl-CoA availability, which inhibits the pyruvate dehydrogenase complex and thereby promotes lactate accumulation. This regulatory interaction, known as the Randle cycle, is an important component of post-exercise metabolic homeostasis and supports glycogen resynthesis (Challa 2025; Randle et al. 1963). While high lactate levels during exercise impair performance, slower lactate clearance during recovery can be beneficial, as lactate serves both as an indirect substrate for glycogen resynthesis and as an important signaling molecule (Brooks et al. 2022). However, the proposed mechanistic regulation via the Randle cycle is plausible but not definitive, as additional physiological processes may also contribute to the observed response. Taken together, our findings suggest that HRR reflects lipid metabolism during post-exercise recovery, as demonstrated by enhanced β-oxidation, reduced upstream lipid accumulation, decreased reliance on ω-oxidation, and more resilient mitochondrial membranes, consistent with more efficient post-exercise lipid metabolism. The proposed mechanistic model is shown in Fig. 5.

Exercise induces profound mitochondrial adaptations, including enhanced biogenesis and expansion of mitochondrial volume (Huertas et al. 2019). These adaptations improve both the capacity and efficiency of mitochondrial β-oxidation (Koves et al. 2005; Molé et al. 1971), and likewise enhance mitochondrial resilience (Chen et al. 2018). At the system level, exercise also improves cardiorespiratory fitness and autonomic regulation (Grässler et al. 2021), both of which are closely related to HRR (Daanen et al. 2012; Jolly et al. 2011). As metabolic, cardiorespiratory, and autonomic systems all adapt with exercise training, it is plausible that individuals with faster HRR also exhibit greater mitochondrial efficiency during post-exercise recovery. However, metabolic fitness in terms of skeletal muscle oxidative capacity does not always coincide with cardiorespiratory fitness, as metabolic and cardiorespiratory adaptations can differ depending on exercise type and intensity (Boyd et al. 2013; Memme et al. 2021). During exercise, sympathetic activation promotes a shift toward glycolytic metabolism, whereas recovery is characterized by parasympathetic reactivation and a transition back to lipid oxidation (Nonogaki 2000; Wangler et al. 2026). As a marker of autonomic function, faster HRR may reflect a more rapid transition from sympathetic to parasympathetic dominance, which may in turn promote an earlier shift toward fatty acid oxidation, potentially accompanied by more efficient mitochondrial lipid metabolism. However, a direct regulatory effect remains speculative, and a parallel adaptation of autonomic and metabolic recovery appears more plausible.

The link between HRR and lipid metabolism reinforces the relevance of HRR as a robust marker of exercise training status and fitness. Reported associations between HRR and cardiorespiratory fitness are not entirely consistent, and HRR is not considered a gold-standard measure of cardiorespiratory fitness, unlike other heart rate-derived metrics or VO₂max (Buchheit et al. 2012). Such inconsistencies may largely reflect differences in HRR calculation methods, many of which oversimplify the complex, non-linear pattern of HRR after exercise. For instance, HRR is most commonly calculated as the difference between heart rate at exercise cessation and the heart rate measured at fixed time points during recovery, such as 60 or 120 s. Because sympathetic withdrawal occurs more gradually over several minutes after exercise cessation, simple HRR calculation methods may capture only part of the overall recovery response (Buchheit 2014; Daanen et al. 2012). In the present study, HRR showed a stronger association with post-exercise lipid metabolism than VO₂peak, which is commonly used as a proxy for VO₂max, the gold-standard measure of cardiorespiratory fitness. Unlike VO₂max, which is strongly effort-dependent, HRR can be assessed independently of maximal effort, making it a more practical measure in many contexts. Consequently, HRR may not only be a more informative marker of cardiorespiratory fitness than previously assumed, but also serve as an accessible indicator of metabolic fitness, an aspect of physical fitness that currently lacks a practical biomarker.

Individuals with slower HRR exhibited a greater accumulation of lipid metabolites, consistent with less efficient β-oxidation. This pattern is physiologically meaningful, as impaired fatty acid oxidation and excess lipid accumulation are associated with cellular dysfunction and metabolic stress, a phenomenon referred to as lipotoxicity (Weinberg 2006). Therefore, β-oxidation efficiency and fatty acid metabolism are tightly linked to several disease states such as mitochondrial dysfunction, insulin resistance, and different heart diseases (Hirabara et al. 2010; Lopaschuk et al. 2010). Fatty acid oxidation is central to cardiac metabolism, representing the predominant source of energy for the heart (Fillmore et al. 2014). For instance, in heart failure, β-oxidation is impaired and a substrate switch occurs (Zhou and Tian 2018). As a consequence of reduced β-oxidation efficiency, a vast spectrum of acylcarnitines and fatty acid dicarboxylates accumulates (Lai et al. 2014). As acylcarnitines have been significantly associated with the disease severity markers NT-ProBNP, urea and cholesterol, acylcarnitines were proposed as potential markers of heart failure (Ruiz et al. 2017). HRR may therefore hold relevance as a marker of metabolic health, but the present findings are limited to a homogeneous cohort of healthy, physically active young men and do not permit conclusions beyond this population.

Several methodological and contextual considerations should be acknowledged when interpreting these findings. As our study was based on plasma metabolite profiling using an untargeted metabolomics approach, we cannot draw conclusions about absolute metabolite concentrations. Consequently, the extent to which the observed changes reflect biologically meaningful alterations in metabolite concentrations remains uncertain. The dataset comprised a large number of measured metabolites but only a modest number of biological samples, creating the so-called p > n problem, that limits statistical power. Consequently, the reported associations should be regarded as exploratory. It also cannot be determined whether the alterations in fatty acid oxidation arise exclusively from skeletal muscle or involve additional contributions from the heart, or even from the liver and kidney, where ω-oxidation primarily occurs.

With regard to HRR, various methods for quantification are available, and simple measures are commonly applied in practice. In our analysis, a monoexponential decay model was used, and this approach may be required to uncover metabolic associations. Nevertheless, it remains uncertain whether other HRR metrics would yield comparable results, which may be more feasible for practical applications. Additionally, heart rate variability (HRV) could not be assessed because the available data were not suitable for reliable HRV analysis. However, HRR remains a validated and widely applied marker of post-exercise autonomic function (Borresen and Lambert 2008). Furthermore, we implemented an active recovery period in our study and we cannot determine whether it influenced our findings, as HRR is typically assessed without such a period. As we included only healthy, physically active young men, we cannot draw conclusions about potential sex differences, age-related effects, or the applicability of HRR in older adults or in populations with metabolic or cardiovascular disease. Women were not included in this study to avoid potential confounding effects of hormonal fluctuations across the menstrual cycle that could influence metabolism and because the available sample size did not allow for adequate stratification by sex. Moreover, autonomic regulation, mitochondrial function, and lipid metabolism are known to change with aging and disease, which may alter the relationship between HRR and post-exercise metabolic recovery. Future studies should investigate whether these associations persist in different cohorts and when using alternative HRR metrics.

## Conclusions

In conclusion, our findings indicate that HRR reflects lipid metabolism during post-exercise recovery. The observed associations become apparent only under conditions of metabolic demand, such as during the later recovery period following vigorous-intensity exercise. Individuals with faster HRR display a metabolic profile consistent with more efficient mitochondrial and peroxisomal lipid metabolism, characterized by enhanced β-oxidation, reduced upstream lipid accumulation, lower reliance on ω-oxidation, and potentially more stable mitochondrial membranes. This metabolic pattern may indicate a more efficient substrate shift, supporting glycogen resynthesis and reducing fatty acid accumulation. Under the applied physiological conditions, HRR serves either as a marker of cardiorespiratory fitness, or as a specific indicator of metabolic fitness. Moreover, given the close link between fatty acid metabolism and metabolic diseases, HRR may be relevant for monitoring metabolic health. However, our results indicate that precise HRR quantification and sufficiently high exercise intensity are required to uncover these associations. Future studies should evaluate the predictive utility of HRR in both exercise and clinical contexts across different populations.

## Supplementary Information

Below is the link to the electronic supplementary material.

Supplementary file1 (DOCX 3953 KB)

Supplementary file2 (DOCX 659 KB)

Supplementary file3 (DOCX 665 KB)

Supplementary file4 (XLSX 13 KB)

Supplementary file5 (XLSX 38 KB)

Supplementary file6 (XLSX 21 KB)

Supplementary file7 (XLSX 17 KB)

Supplementary file8 (DOCX 18 KB)

## Data Availability

The R script underlying this analysis is publicly available at: https://zenodo.org/records/18195821. All data will be made available upon reasonable request to the corresponding author.

## Abbreviations

CME: Continuous moderate-intensity exercise
CVE: Continuous vigorous-intensity exercise
HRinterval: HR sampling interval (10 vs. 15 s)
HRR: Heart rate recovery
HRpeak: Heart rate at exercise cessation
HRrest: Resting heart rate
Metbaseline: Baseline metabolite levels at t0
VO₂peak: Peak oxygen uptake
τ: Time constant (in seconds) of the monoexponential decay model
