# Supplementary material for: Heart rate recovery as a marker of post-exercise lipid metabolism following moderate- and vigorous-intensity exercise
Source: Eur J Appl Physiol. 2026 Mar 7;126(7):3747–61. doi: 10.1007/s00421-026-06184-y (PMC13380569; doi:10.1007/s00421-026-06184-y)

**Supplementary Figure 1**

Heart rate curves with corresponding monoexponential model fits and residuals for each participant and exercise session.

**Heart rate recovery as a marker of** **post-exercise lipid metabolism following moderate- and vigorous-intensity exercise**

Dirk Weber^1^, Paola G. Ferrario^2^, Achim Bub^1,2^

^1^ Institute of Sports and Sports Science, Karlsruhe Institute of Technology, Karlsruhe, Germany,

^2^ Department of Physiology and Biochemistry of Nutrition, Max Rubner-Institute, Karlsruhe, Germany

*European Journal of Applied Physiology (Springer)*

**Corresponding author:**

Dirk Weber

Karlsruhe Institute of Technology (KIT)

Engler-Bunte-Ring 15

76131 Karlsruhe (Germany)

[dirk.weber@kit.edu](mailto:dirk.weber@kit.edu)

# Participant 1 – CME trial

## Mono-exponential decay model fit


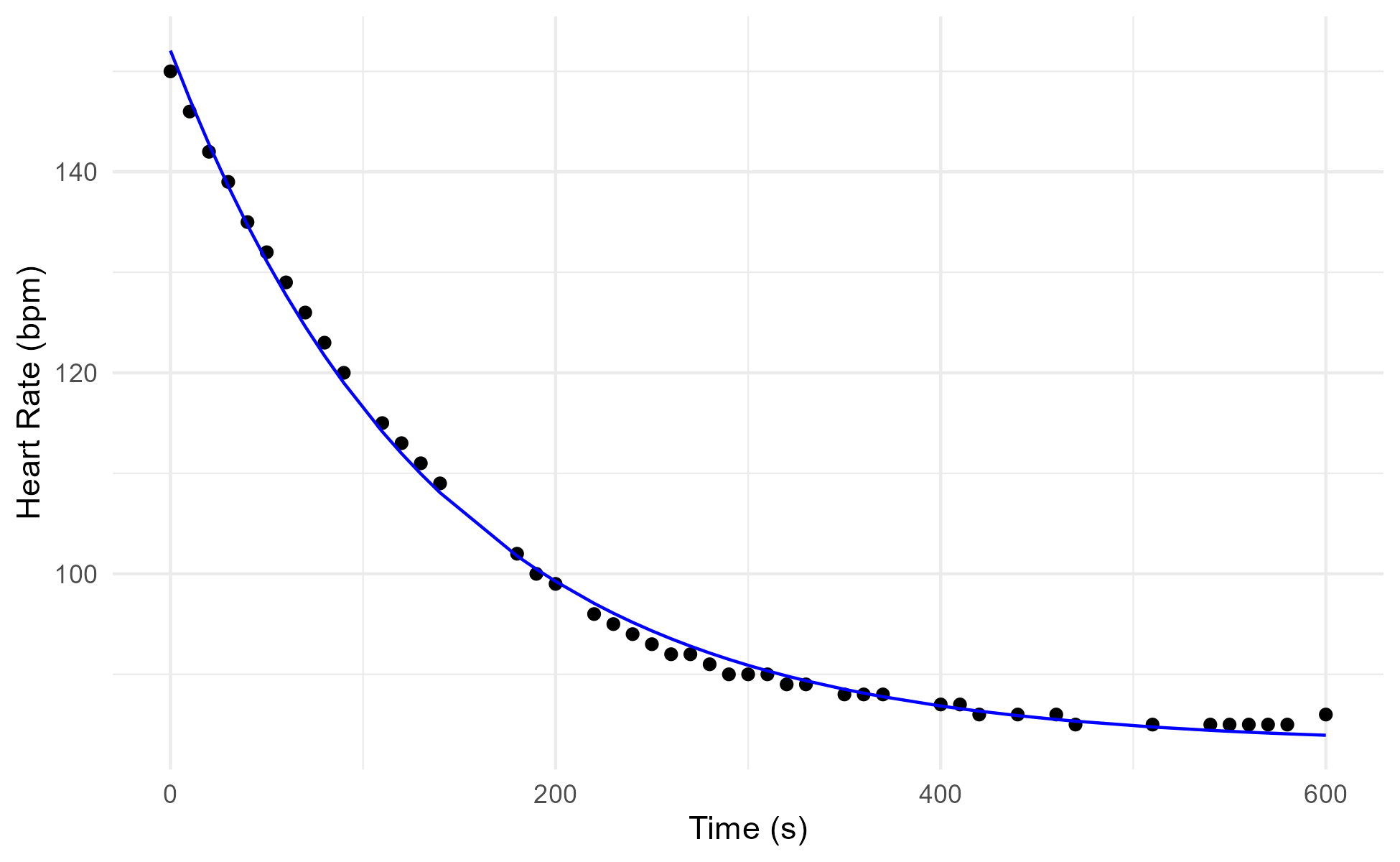


## Residuals of model fit


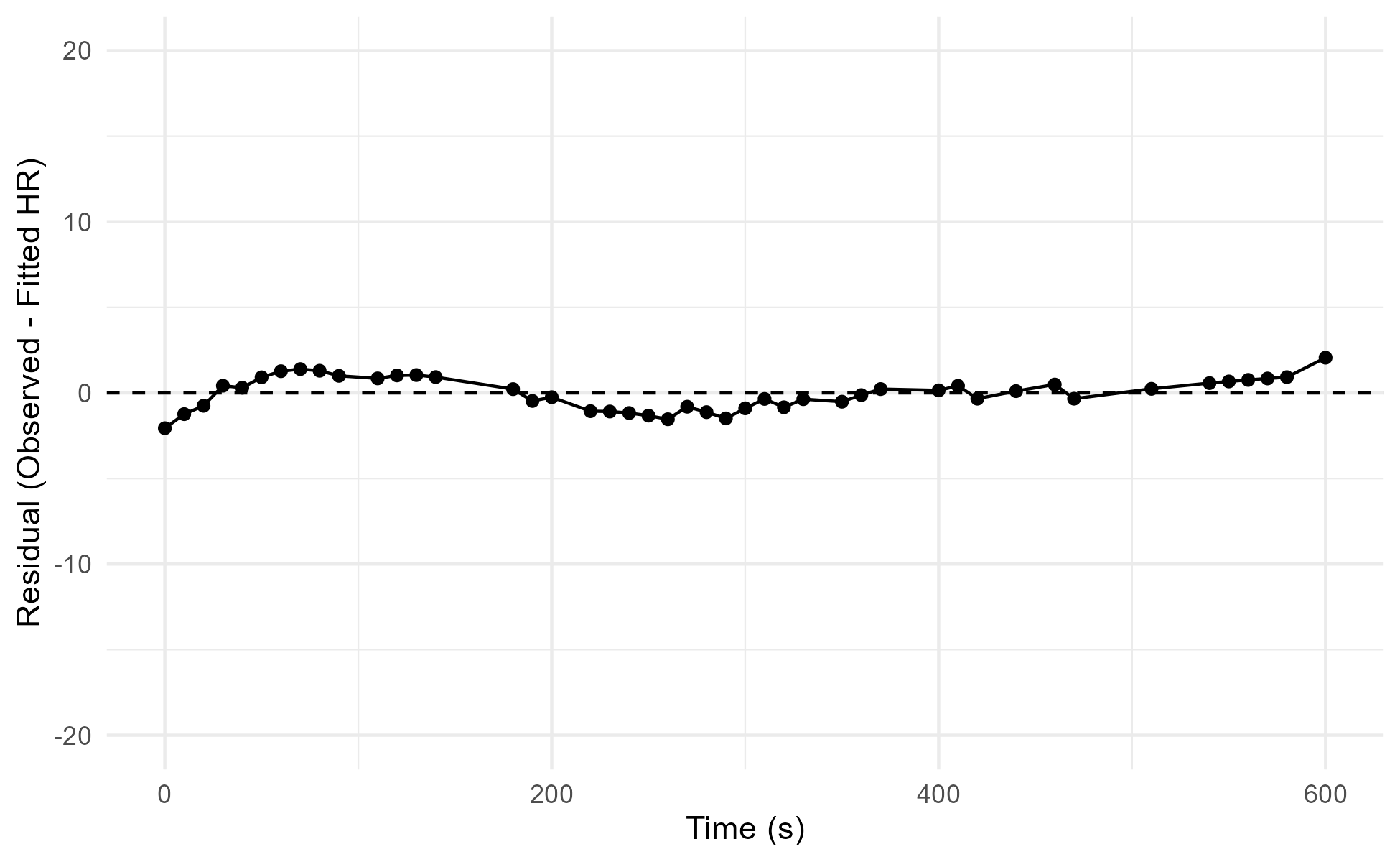


# Participant 1 – CVE trial

## Mono-exponential decay model fit


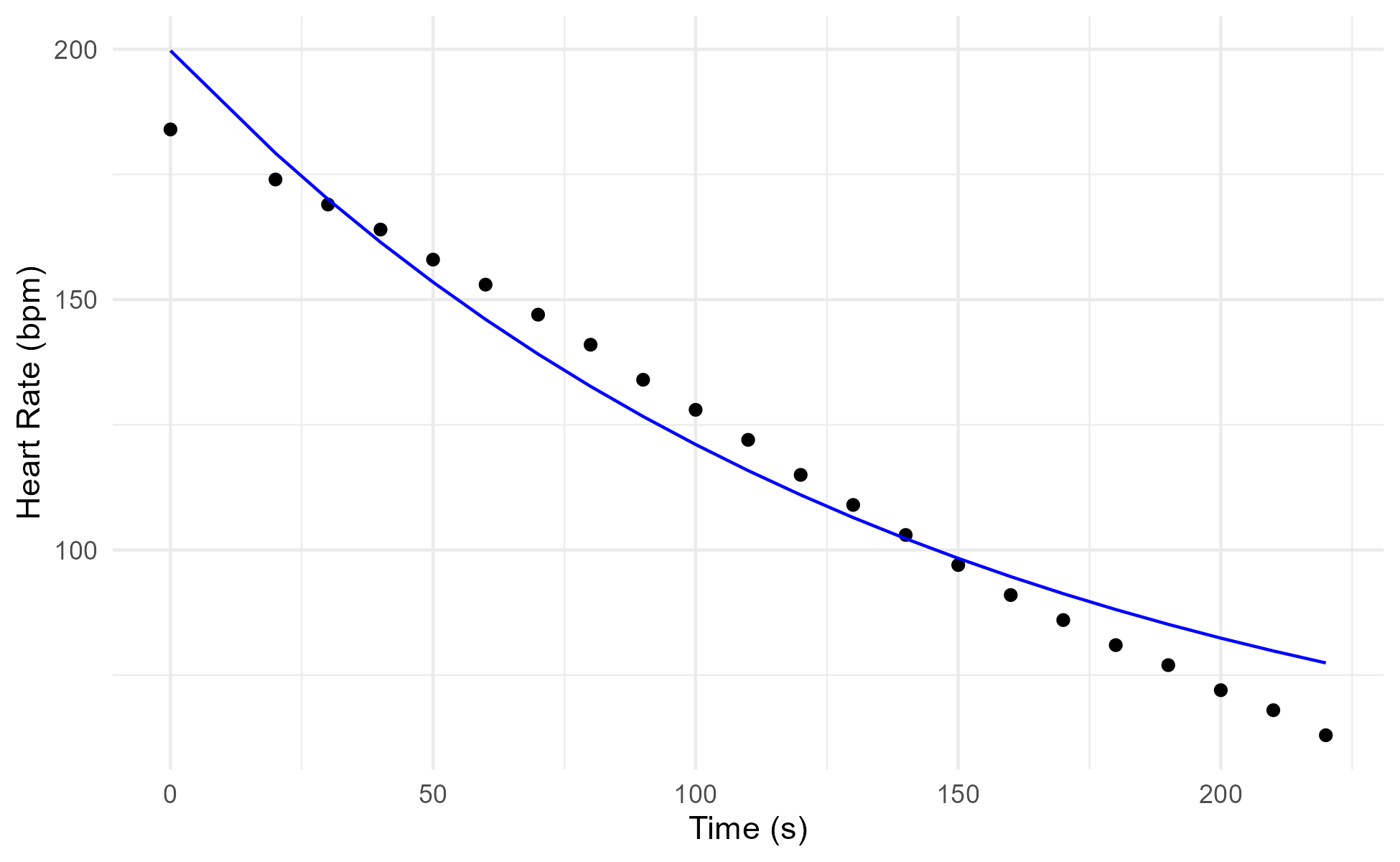


## Residuals of model fit


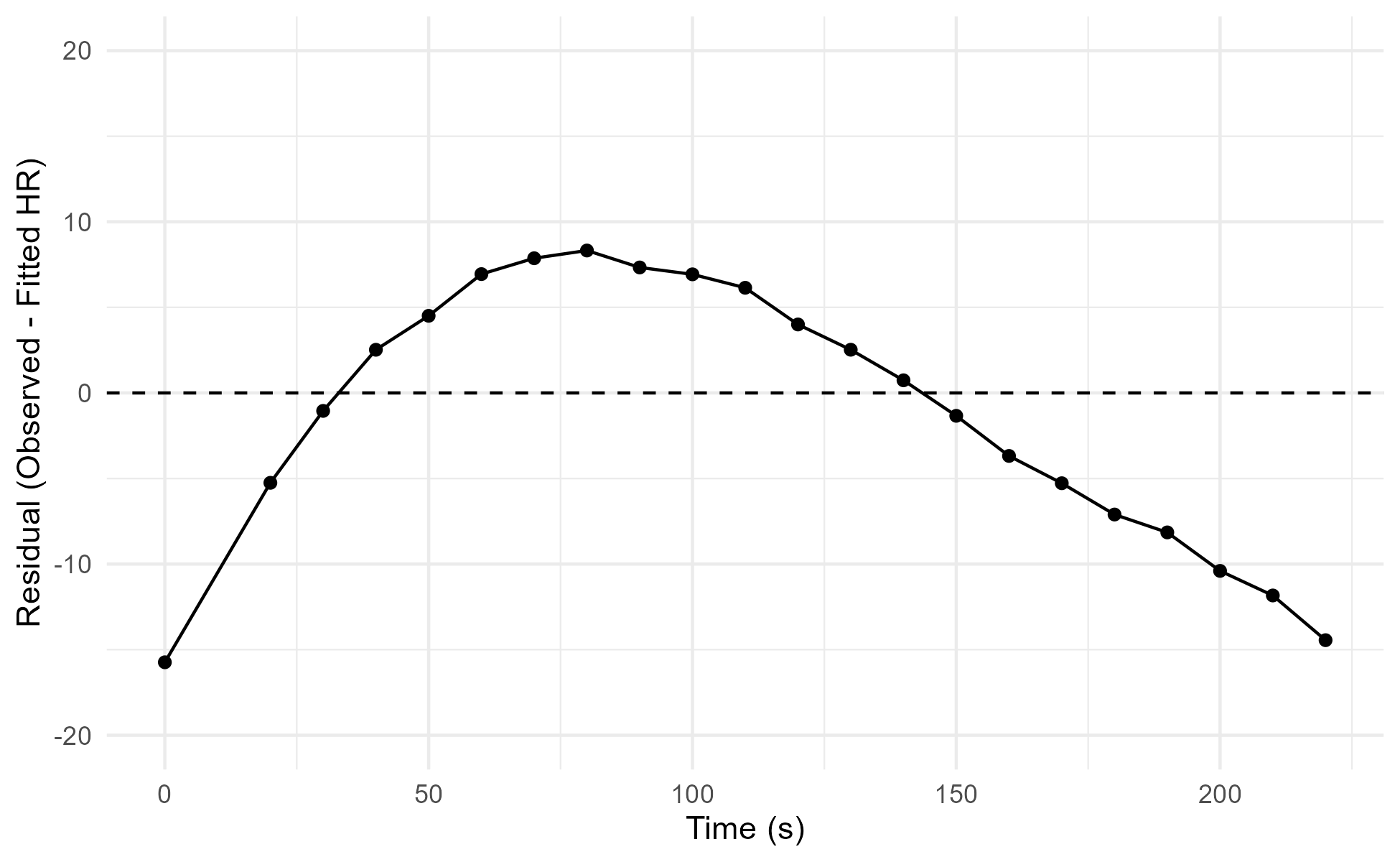


# Participant 2 – CME trial

## Mono-exponential decay model fit


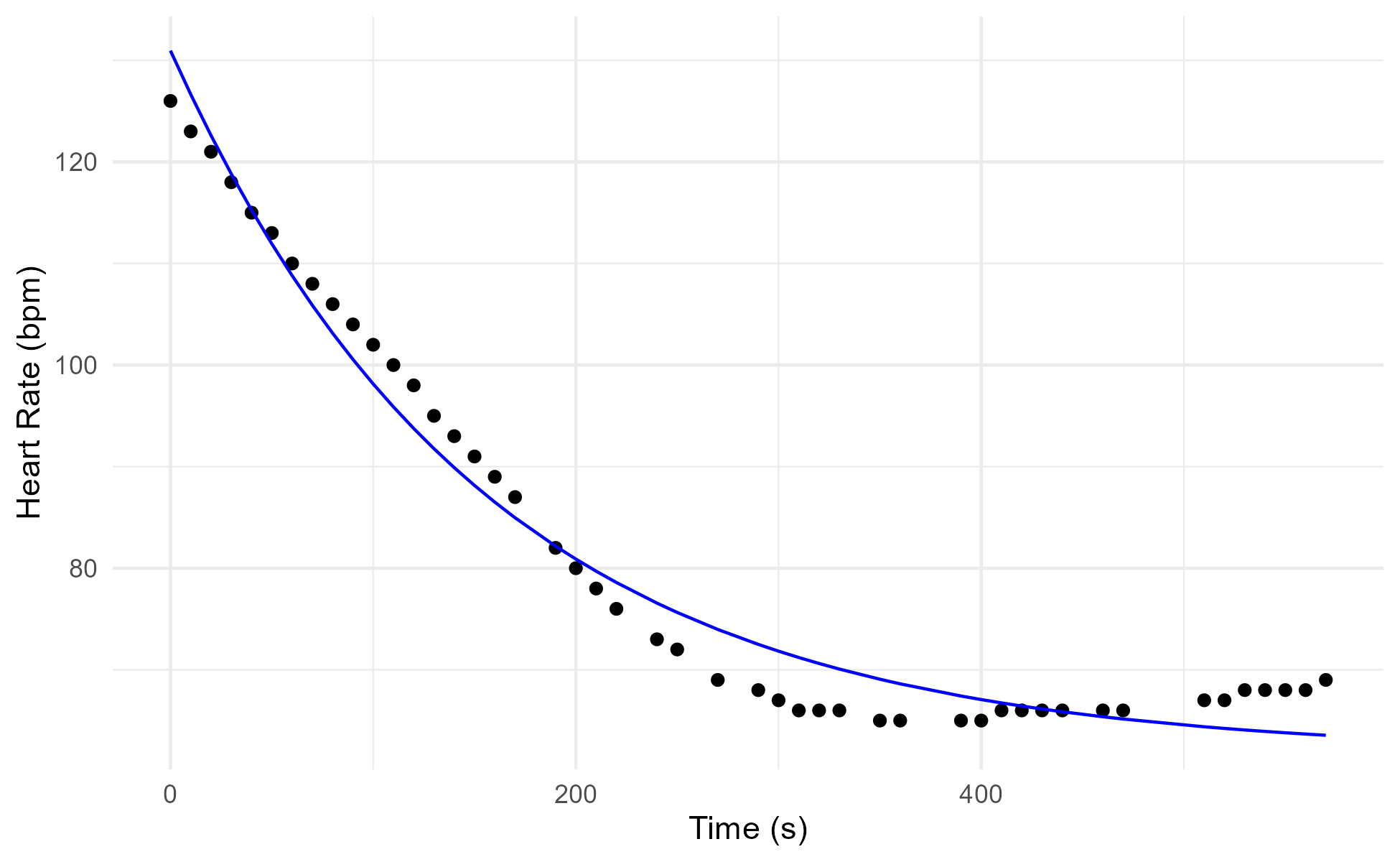


## Residuals of model fit


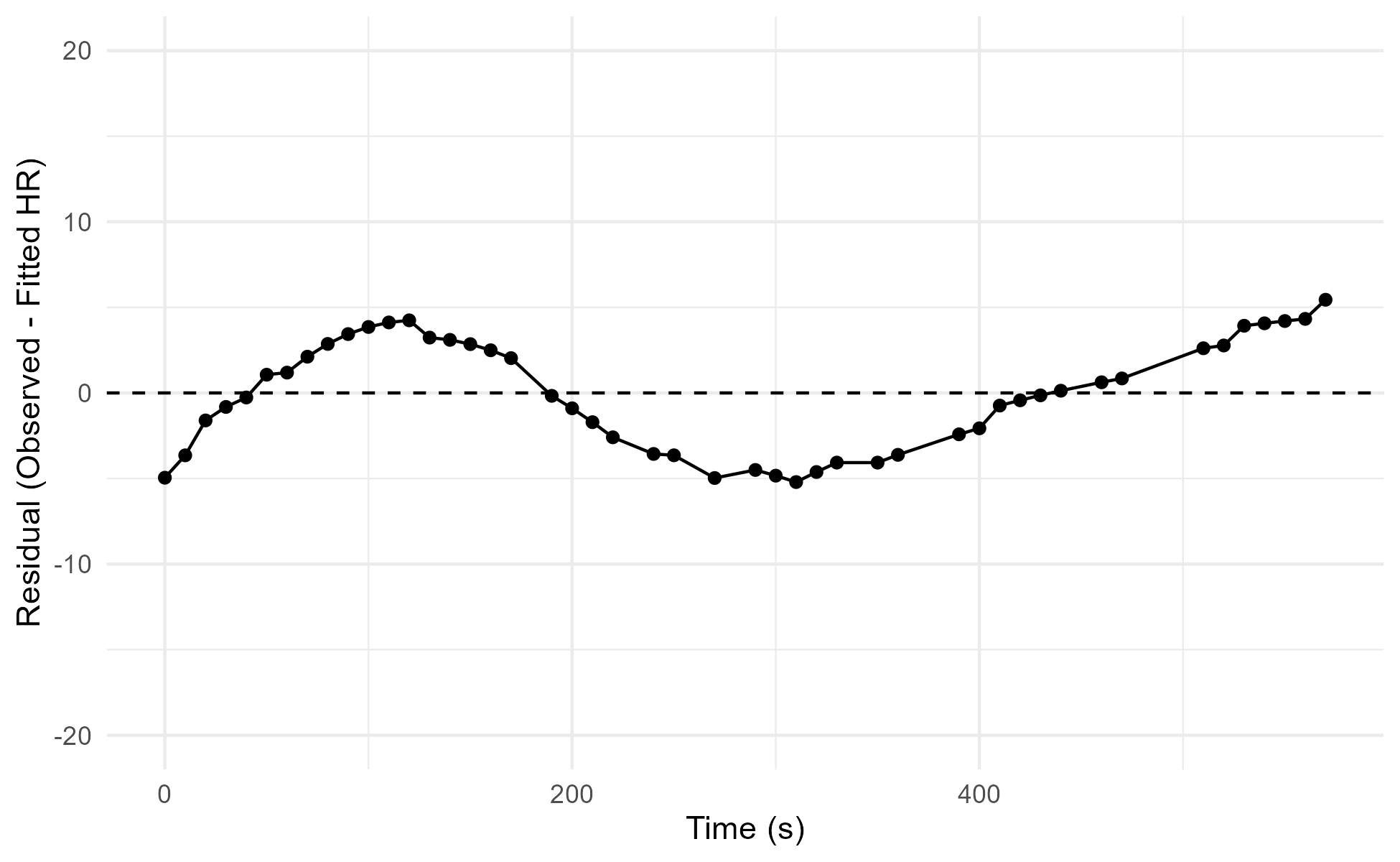


# Participant 2 – CVE trial

## Mono-exponential decay model fit


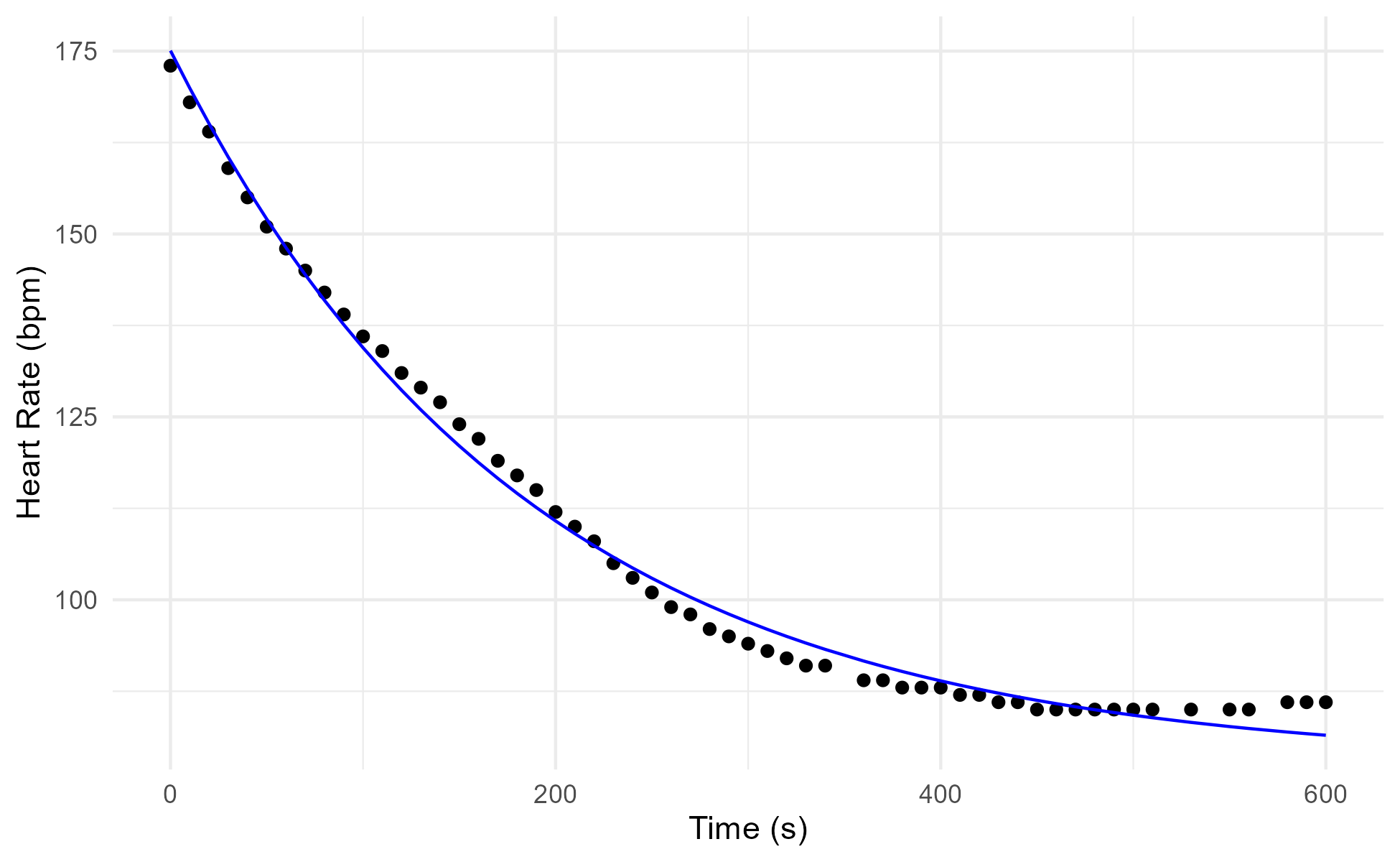


## Residuals of model fit


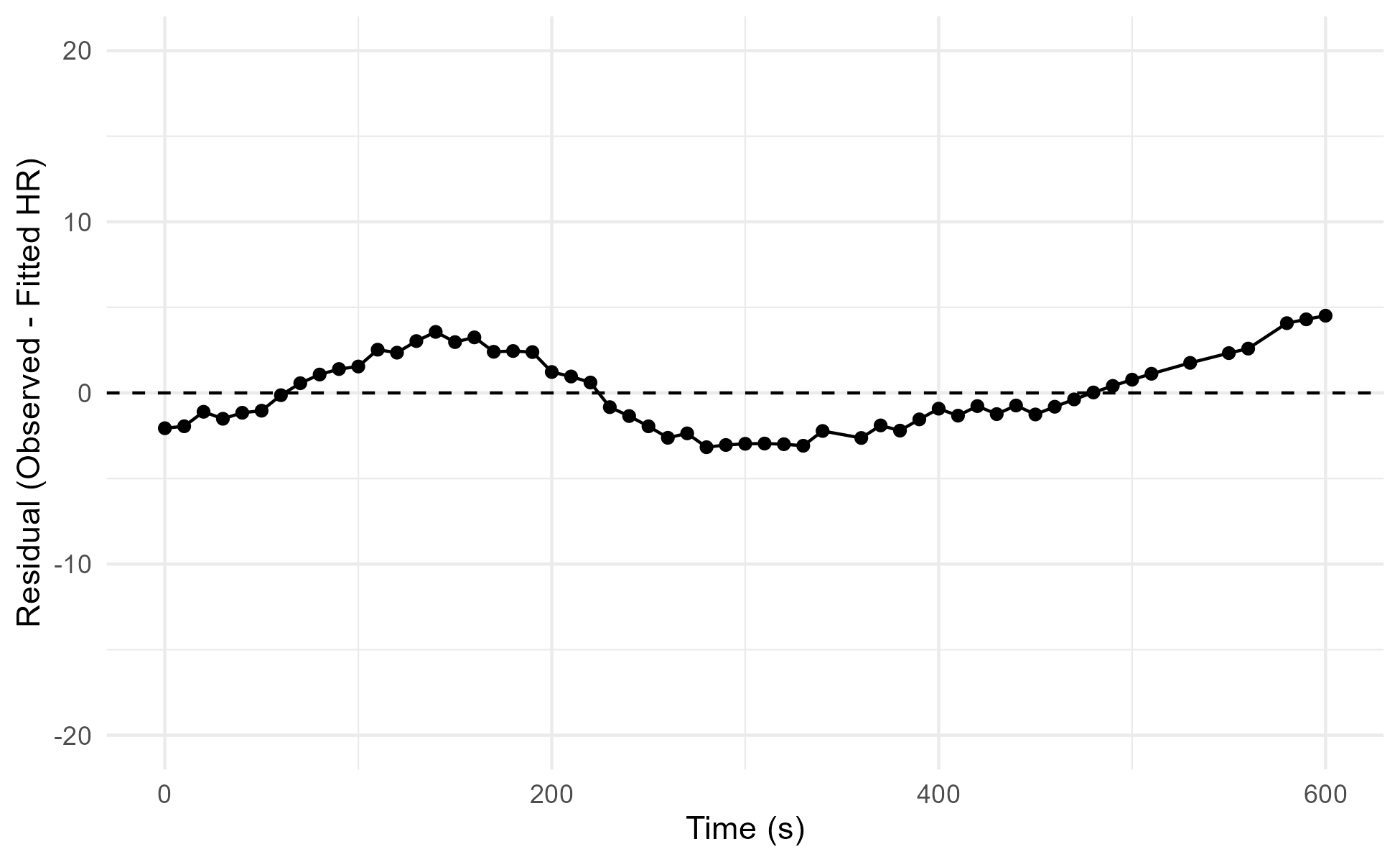


# Participant 3 – CVE trial

## Mono-exponential decay model fit


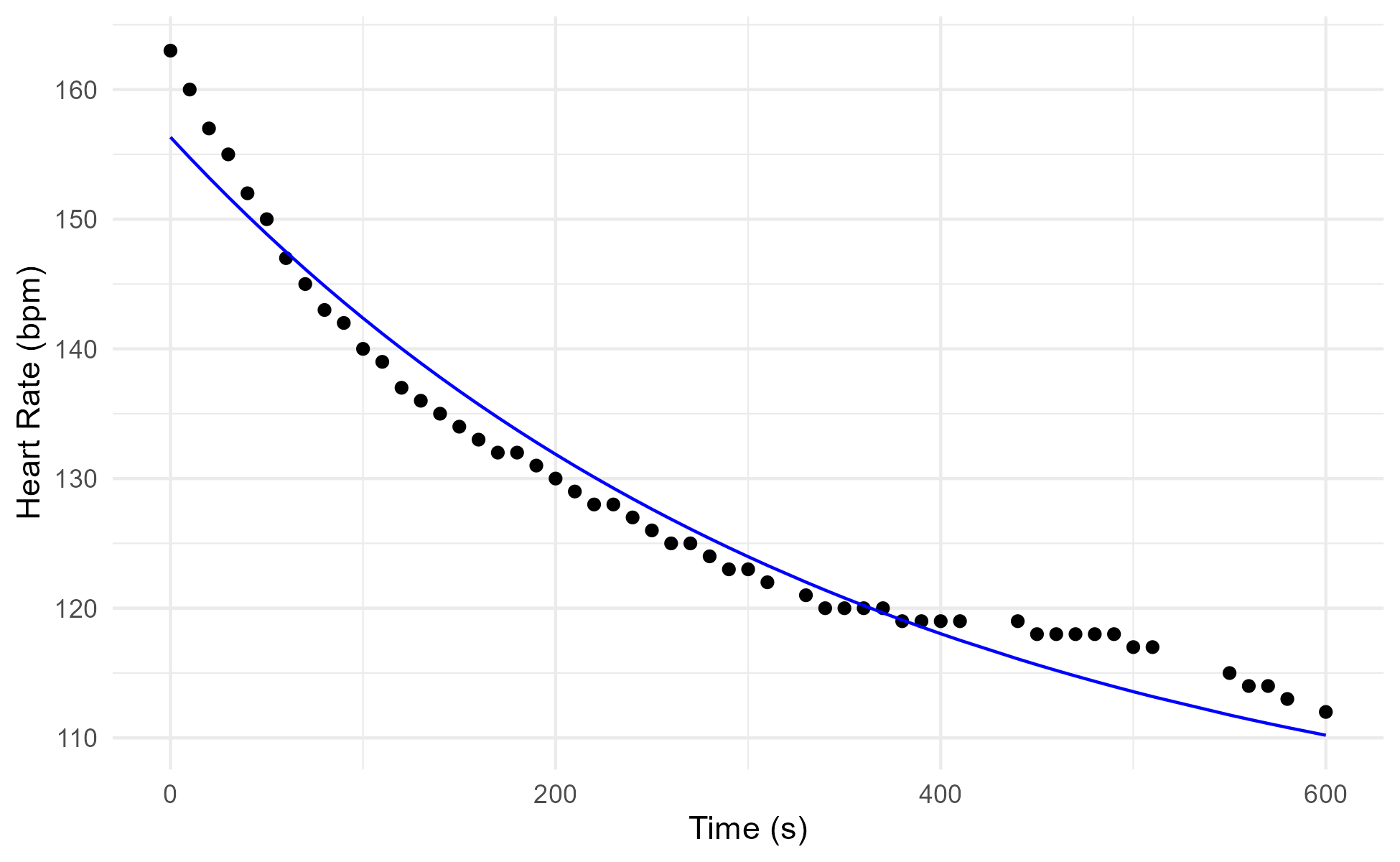


## Residuals of model fit


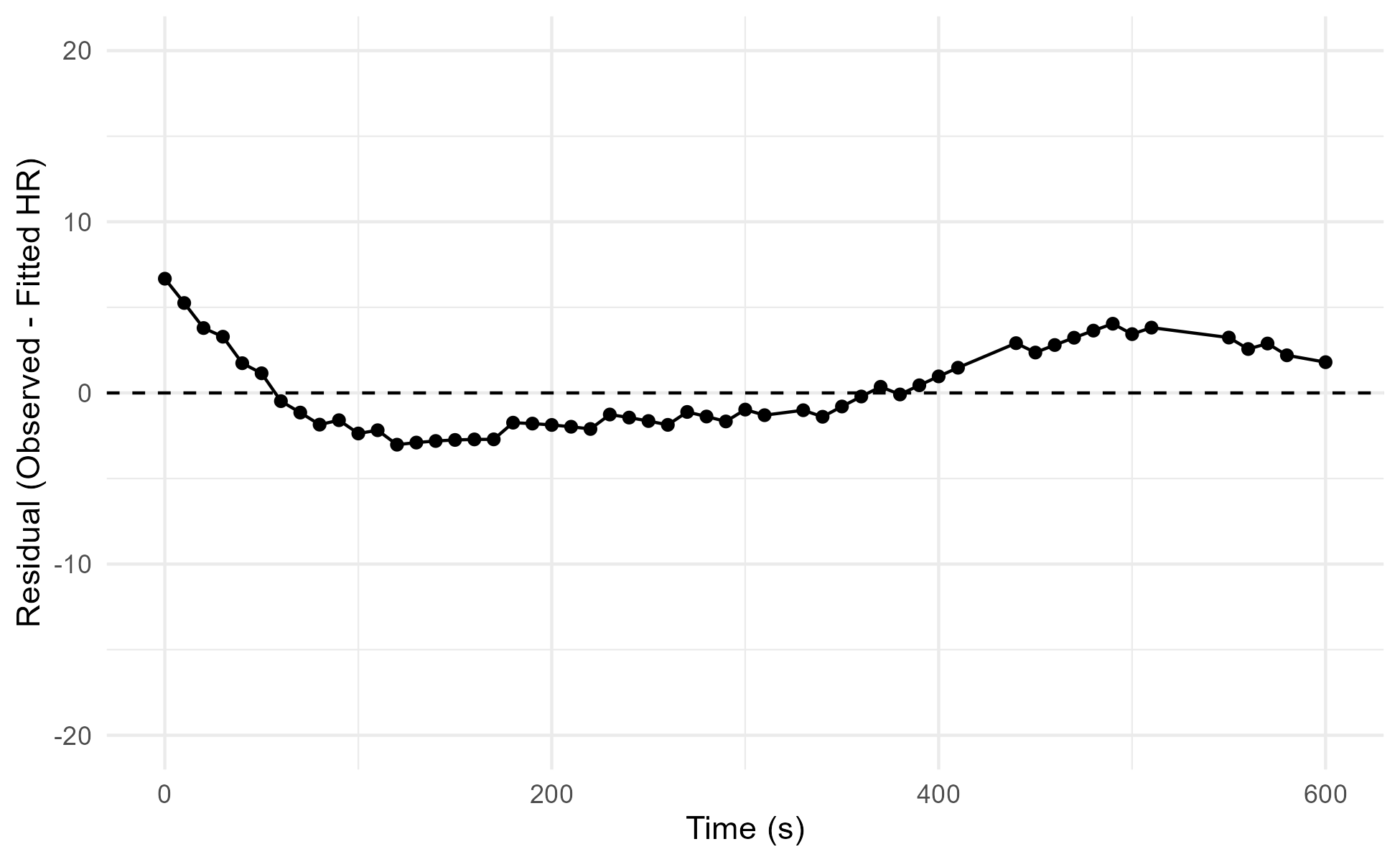


# Participant 4 – CME trial

## Mono-exponential decay model fit


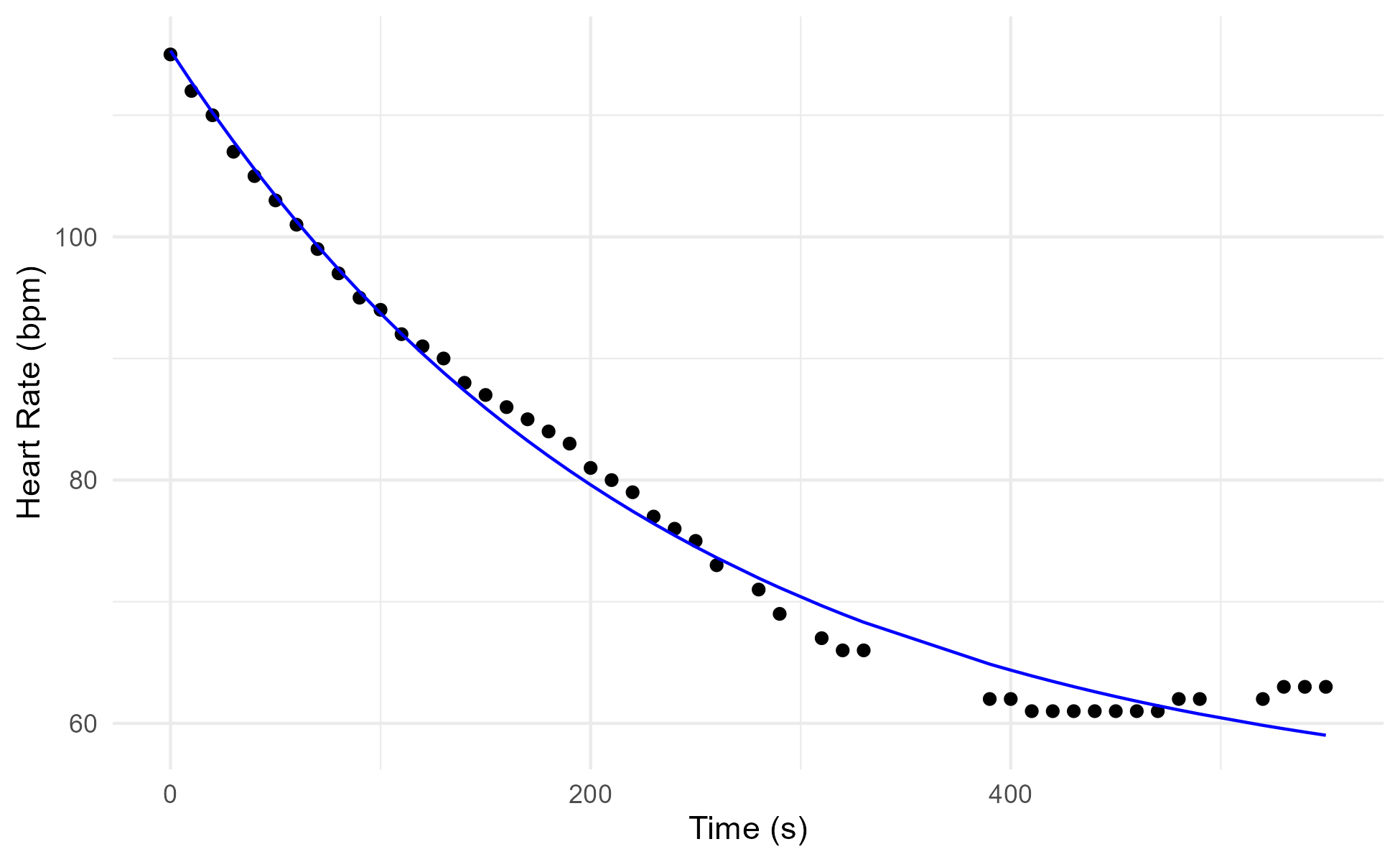


## Residuals of model fit


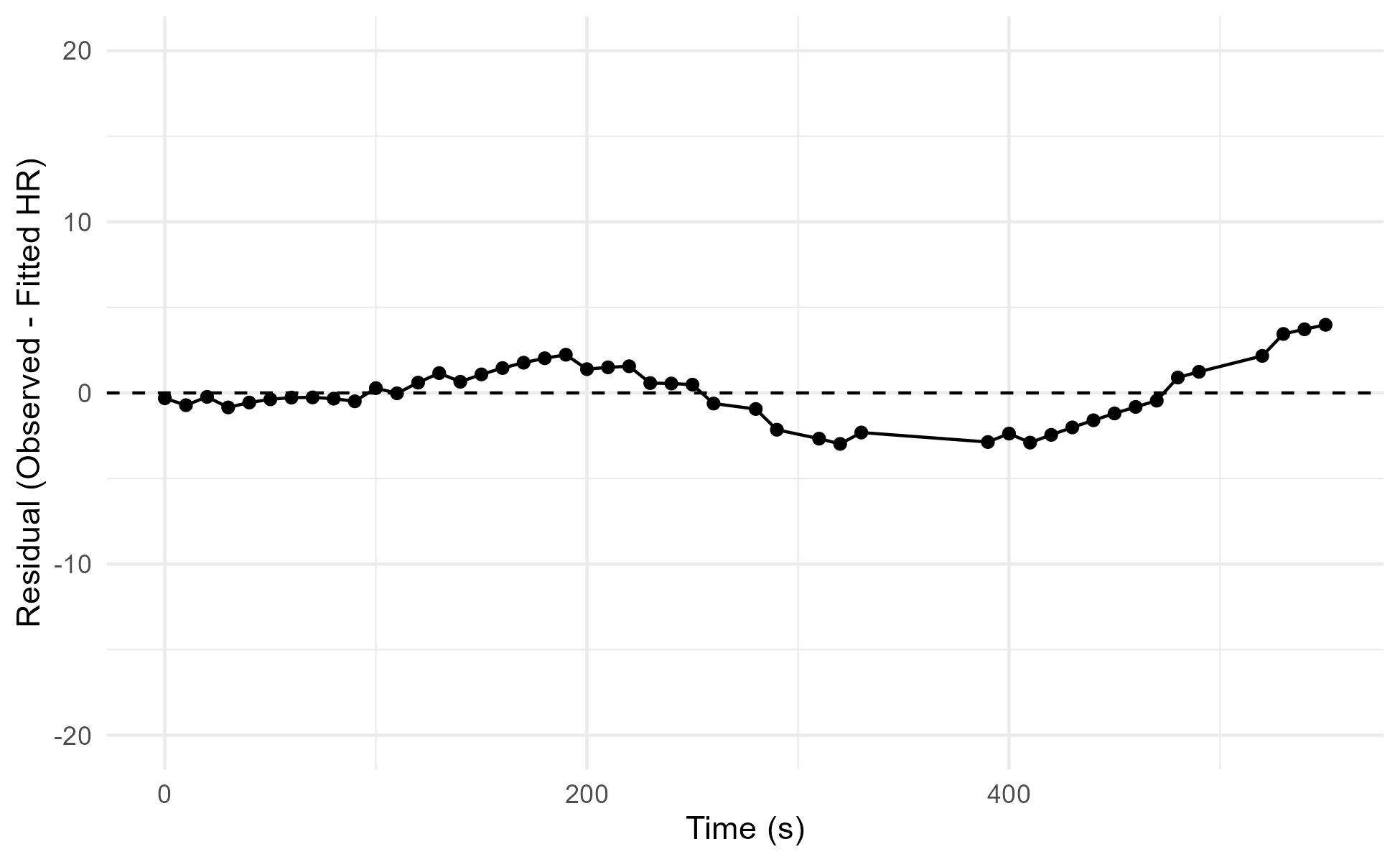


# Participant 5 – CME trial

## Mono-exponential decay model fit


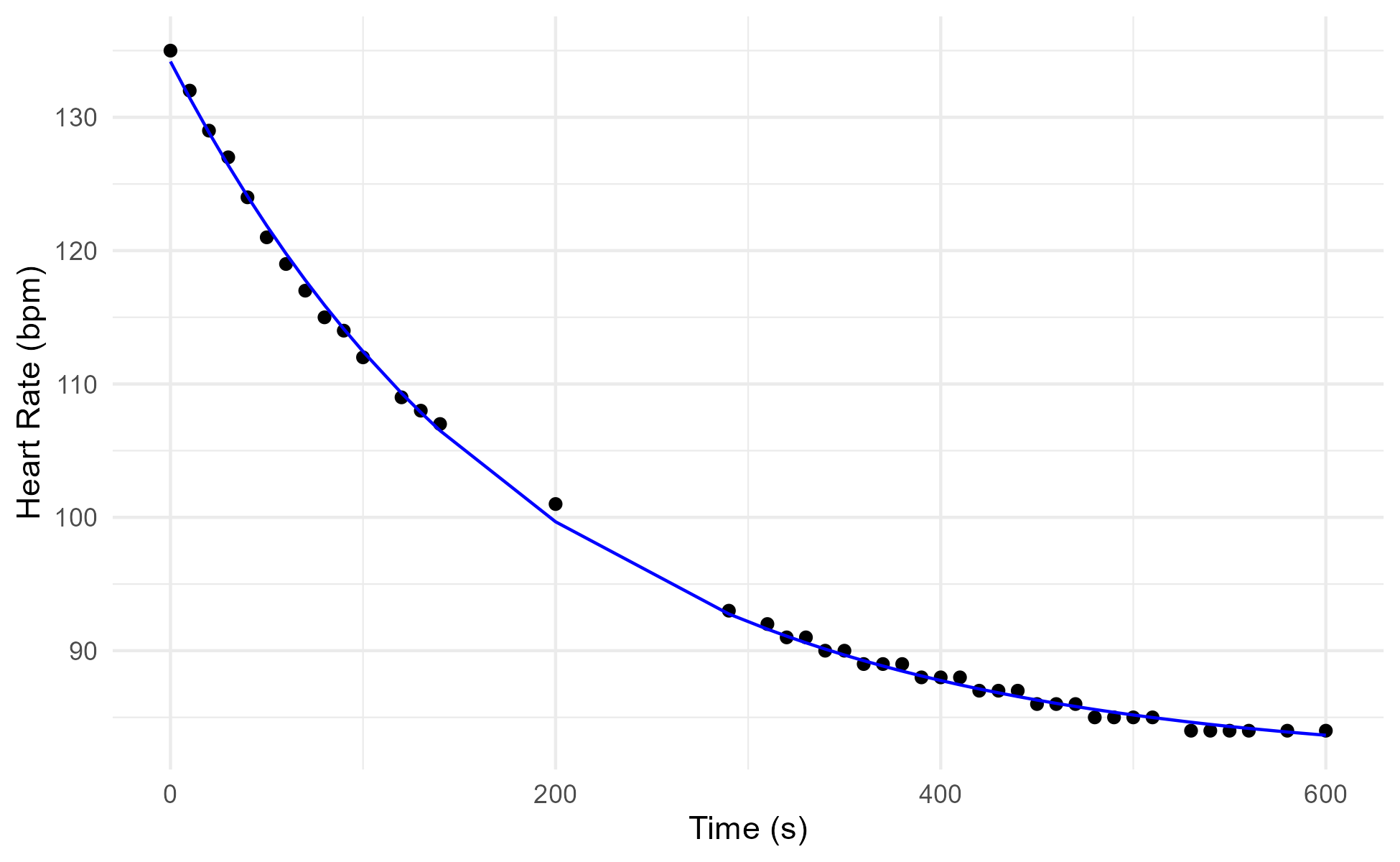


## Residuals of model fit


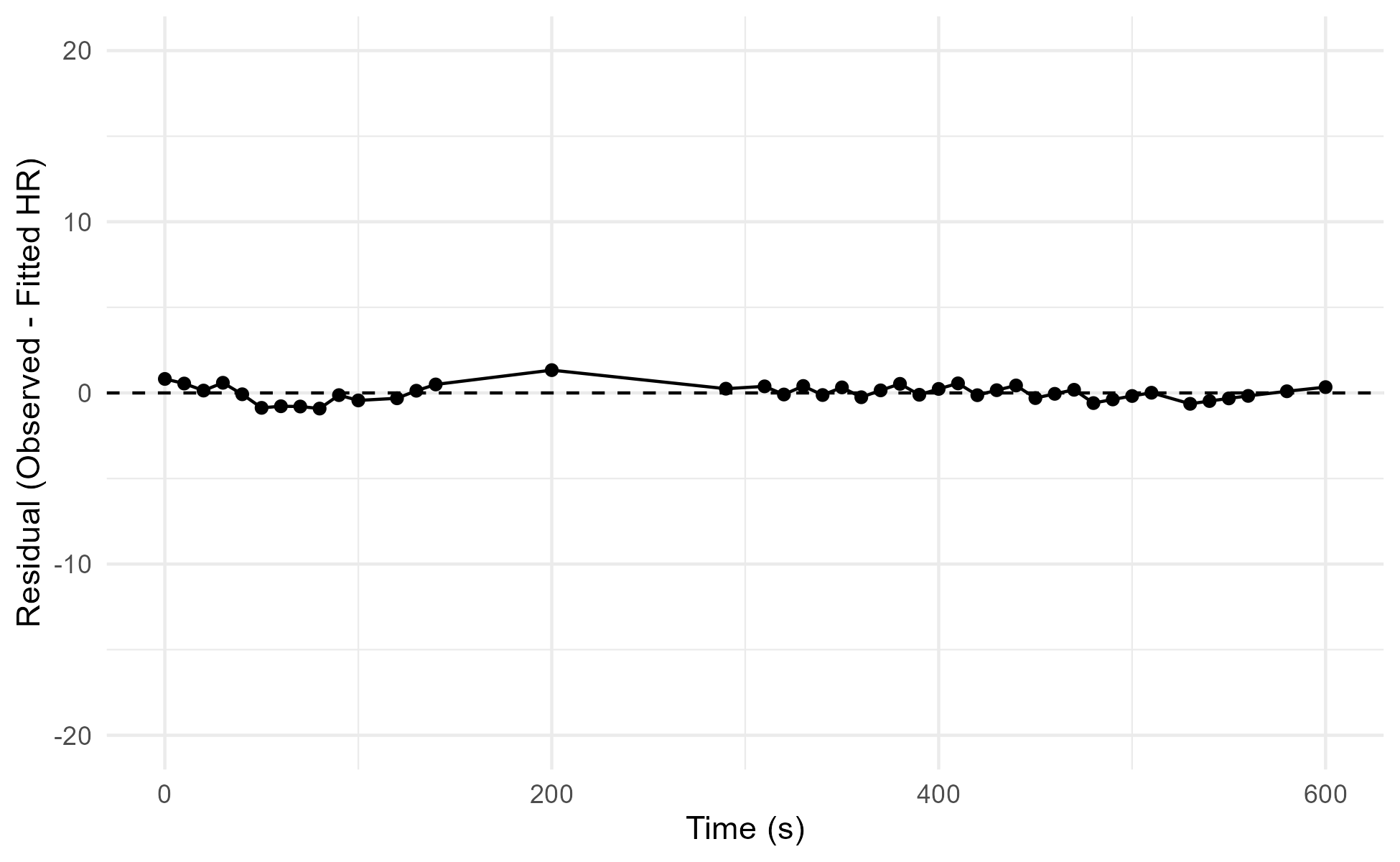


# Participant 5 – CVE trial

## Mono-exponential decay model fit


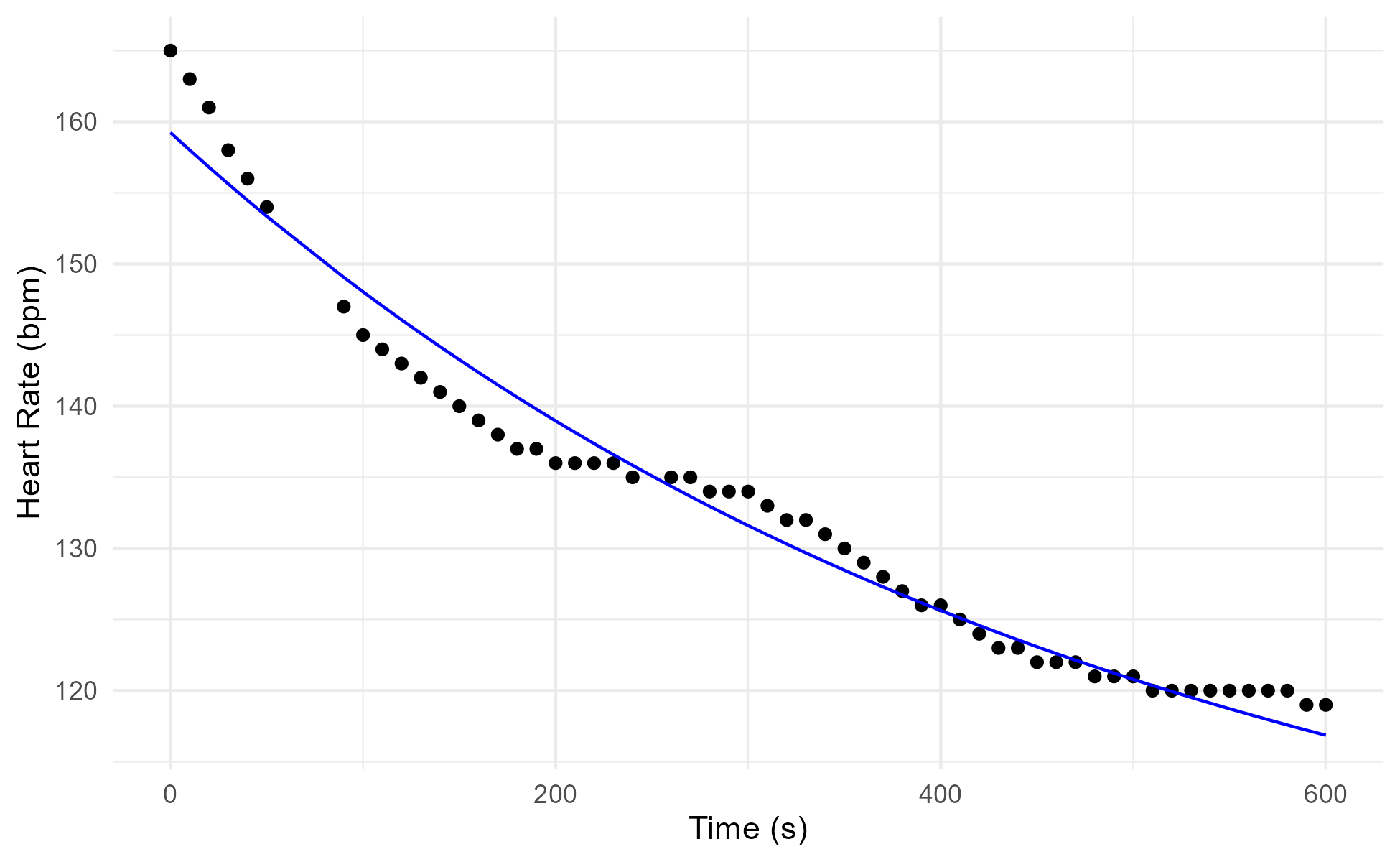


## Residuals of model fit


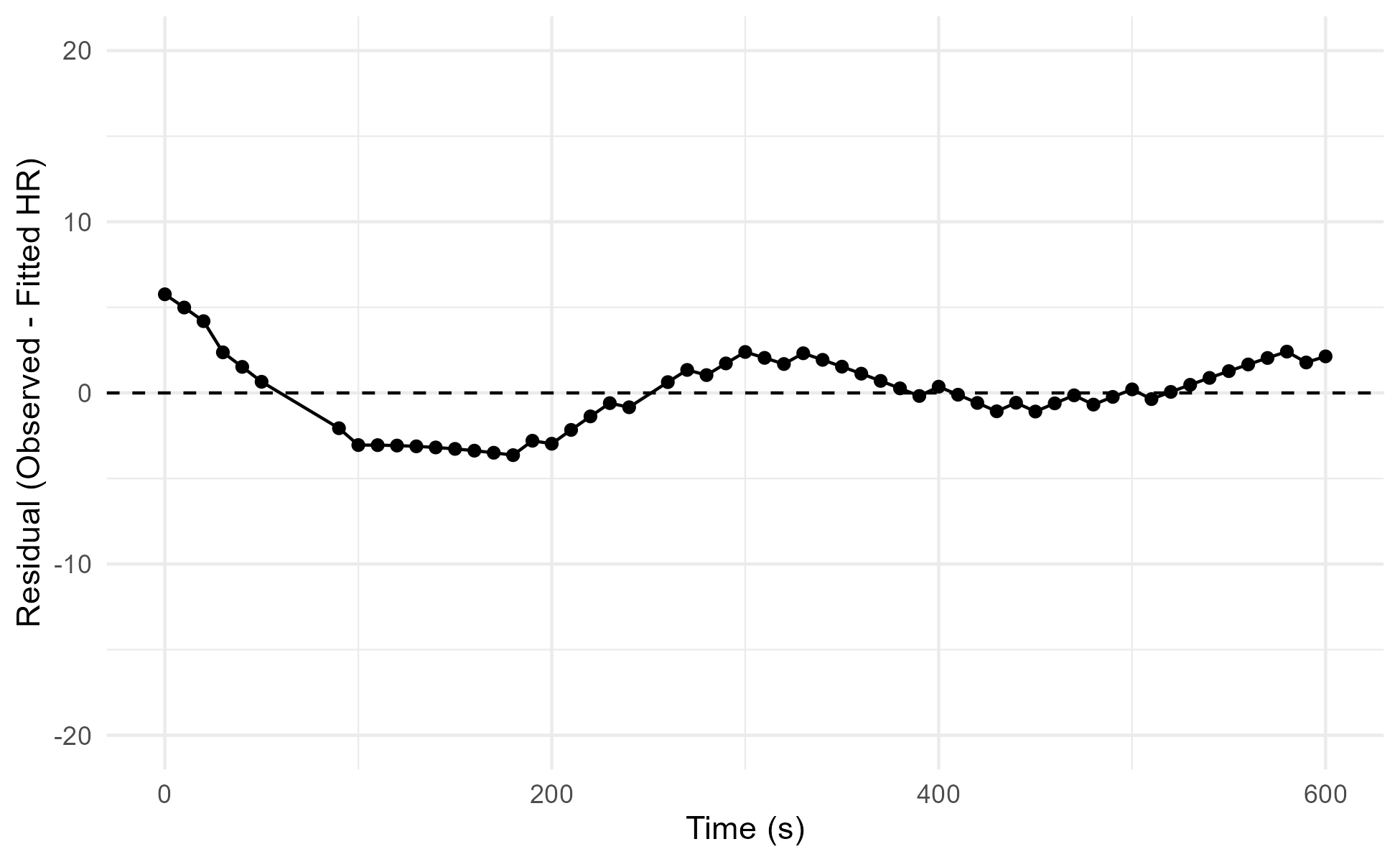


# Participant 6 – CME trial

## Mono-exponential decay model fit


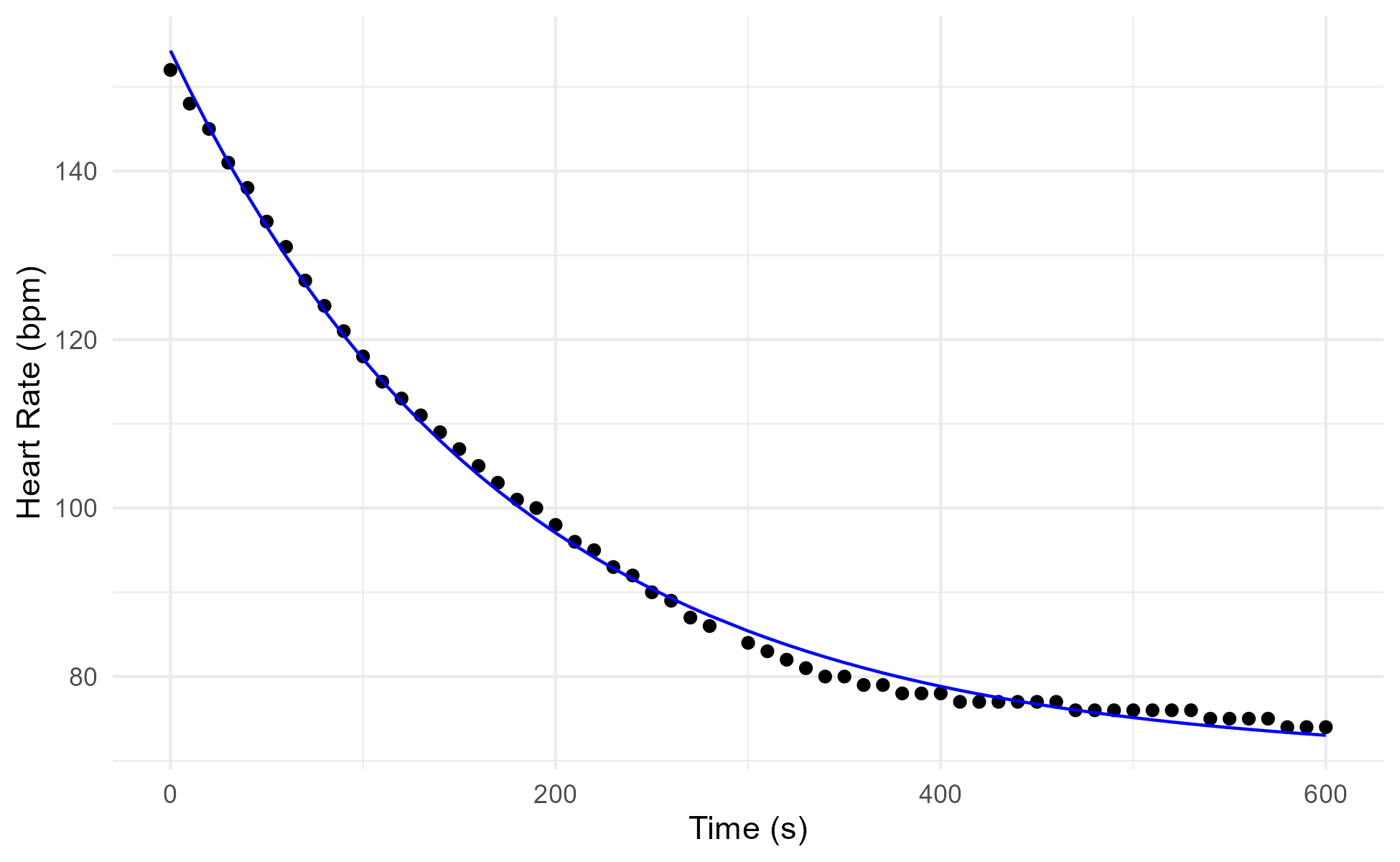


## Residuals of model fit


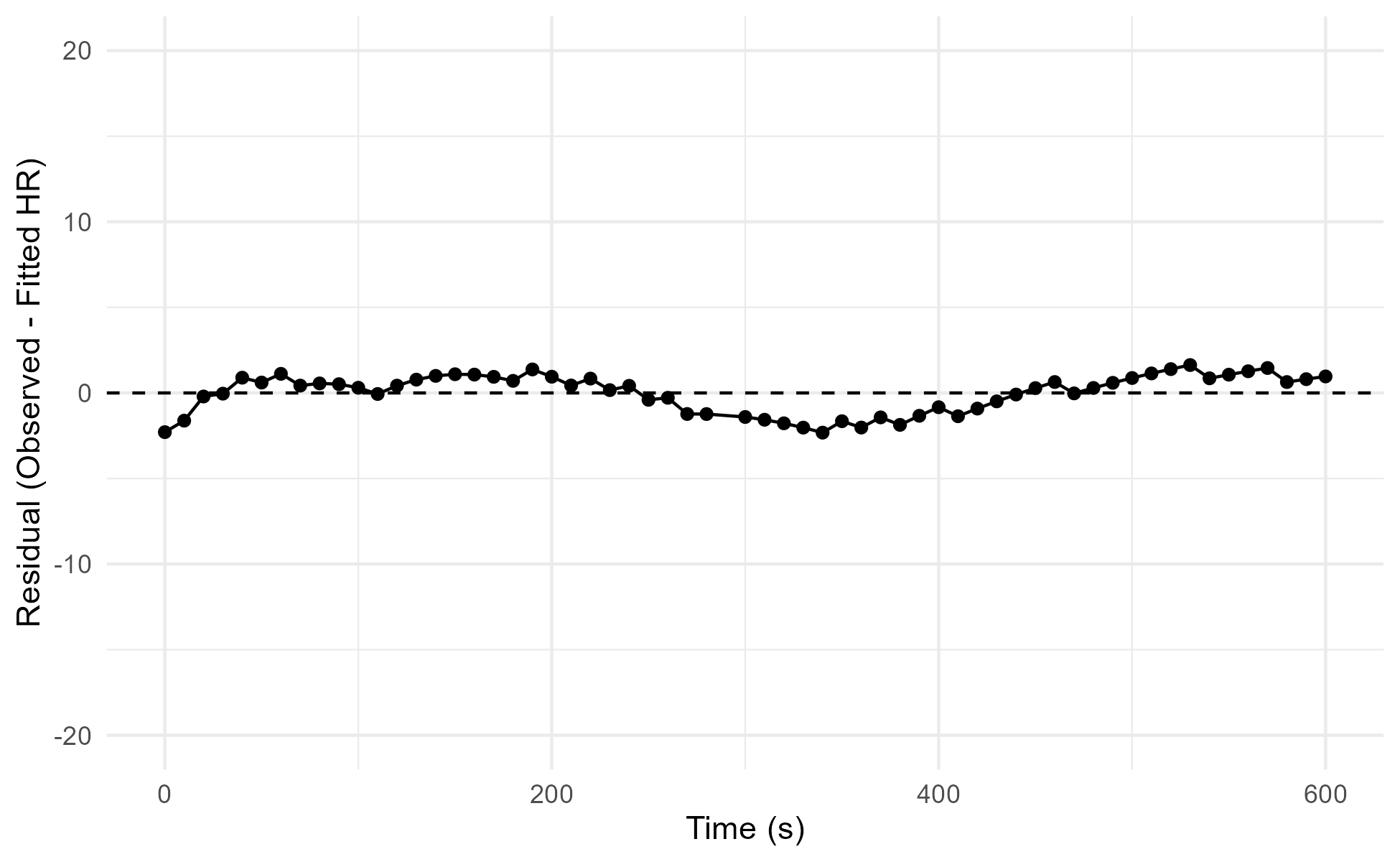


# Participant 6 – CVE trial

## Mono-exponential decay model fit


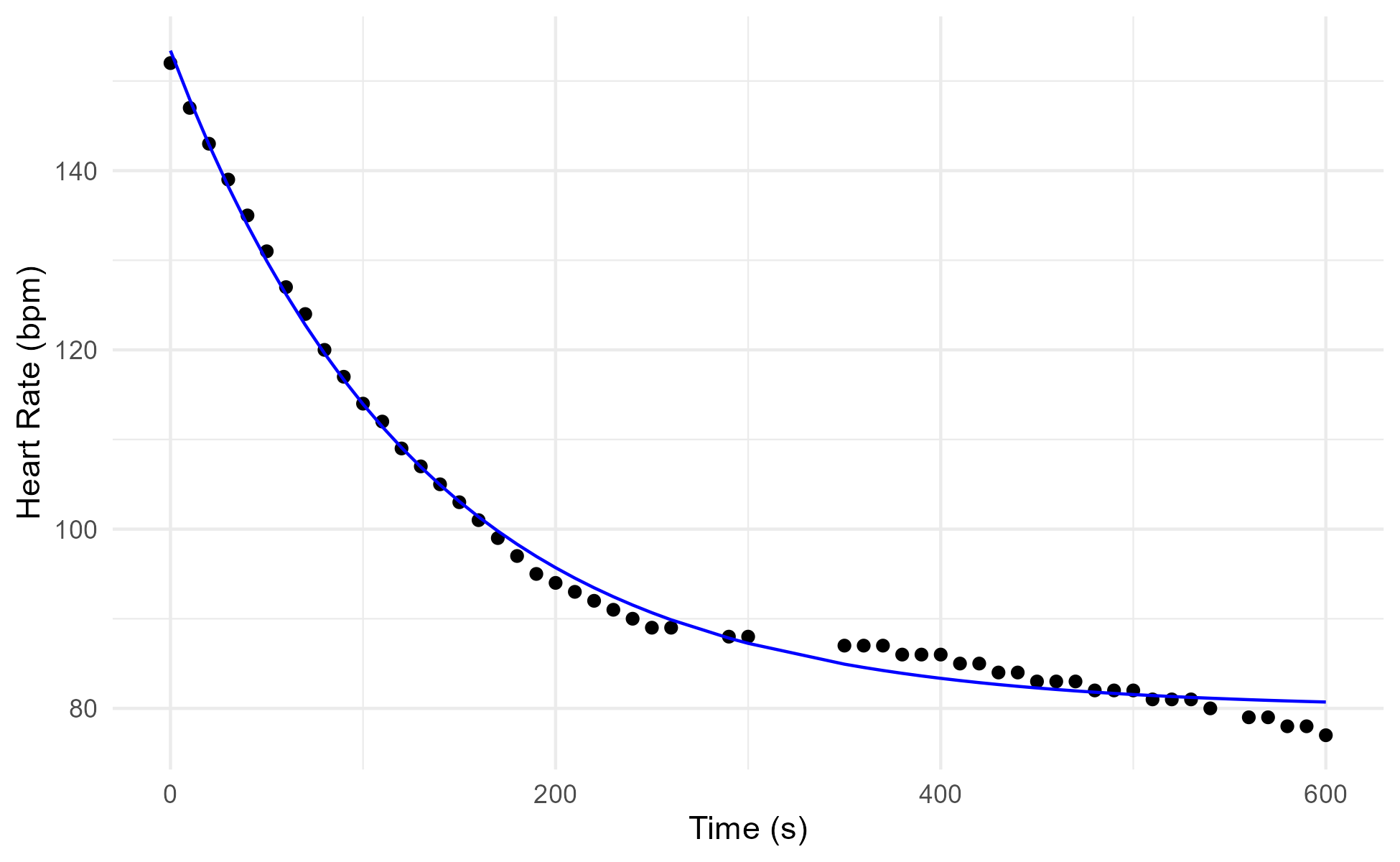


## Residuals of model fit


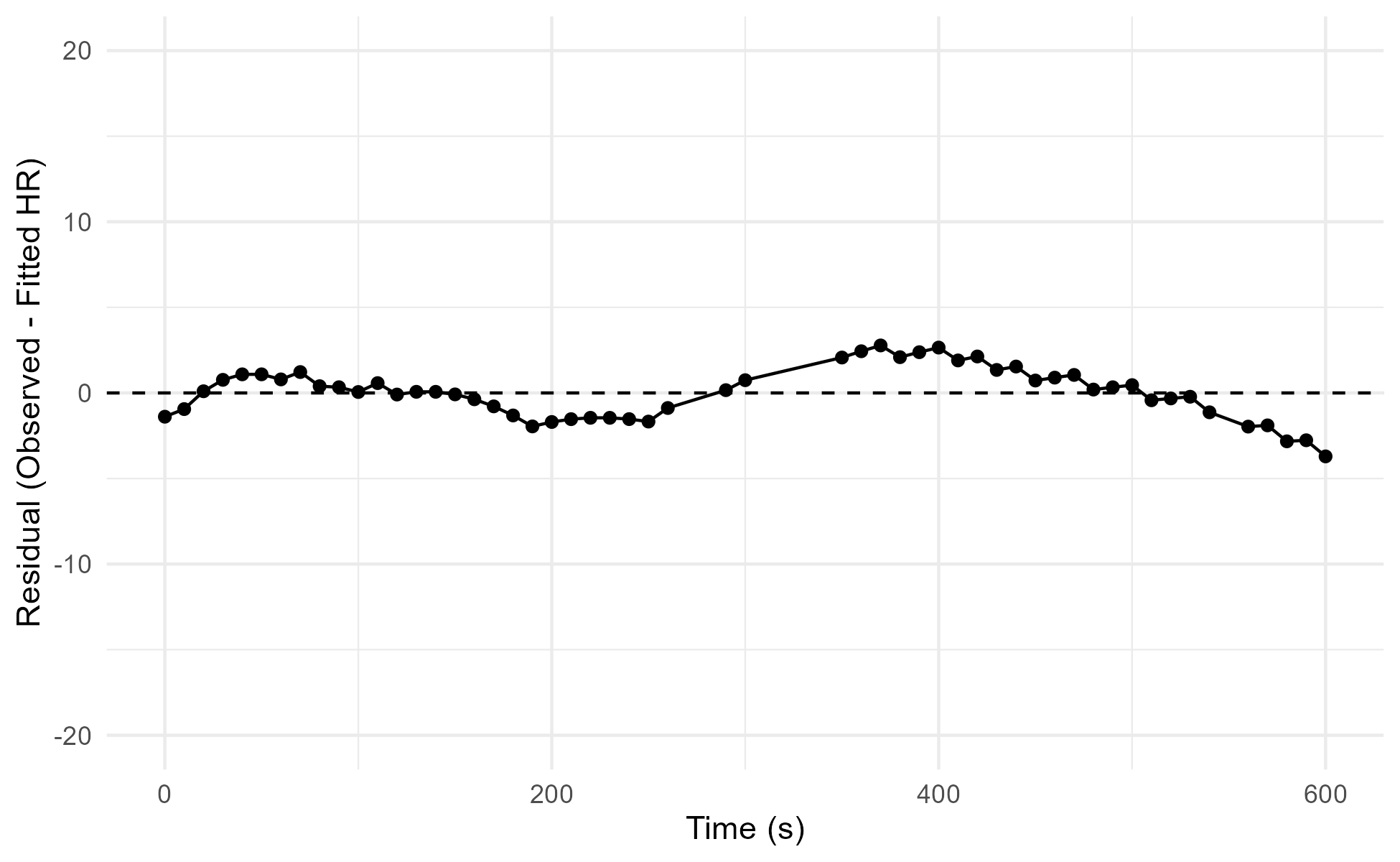


# Participant 7 – CME trial

## Mono-exponential decay model fit


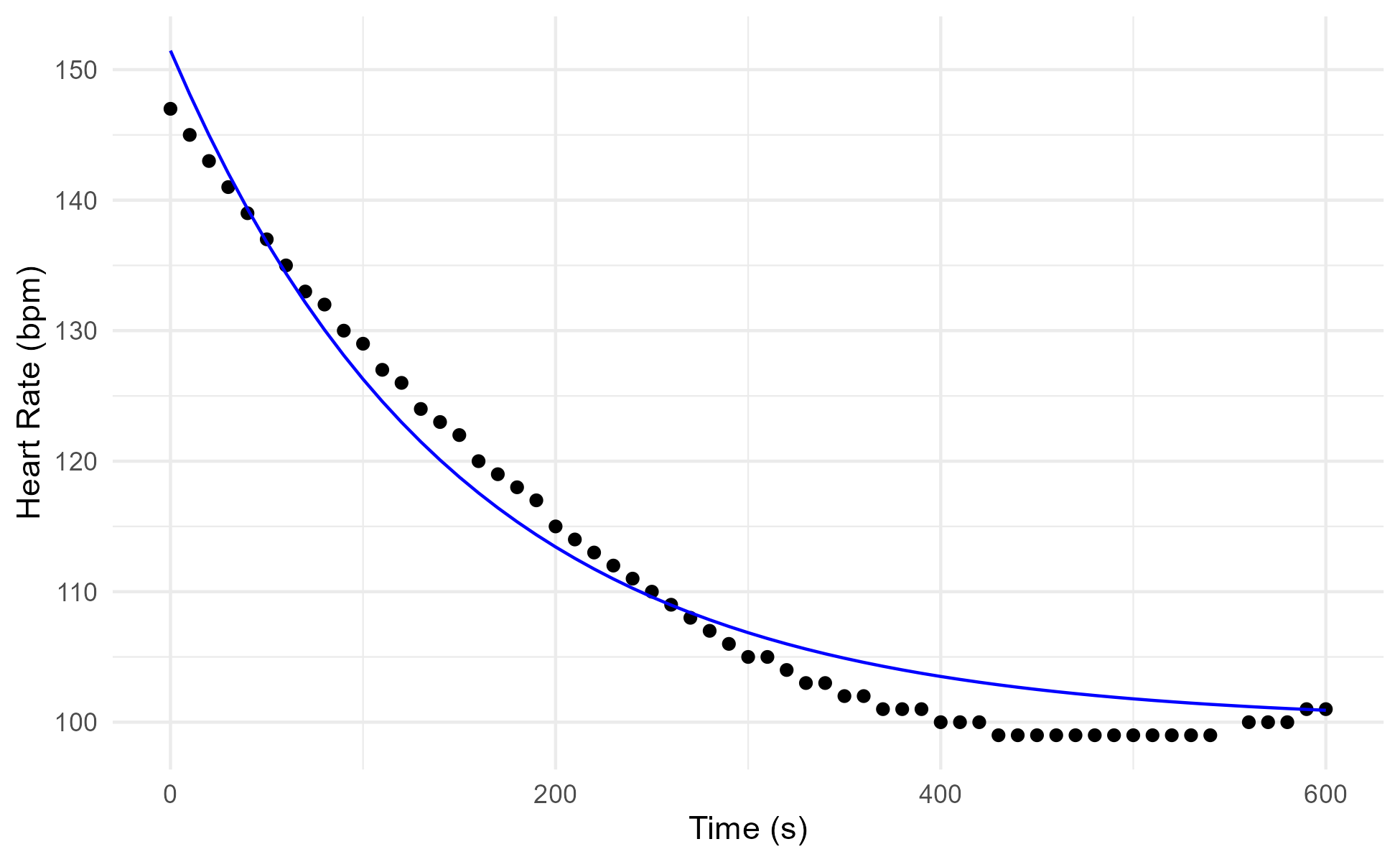


## Residuals of model fit


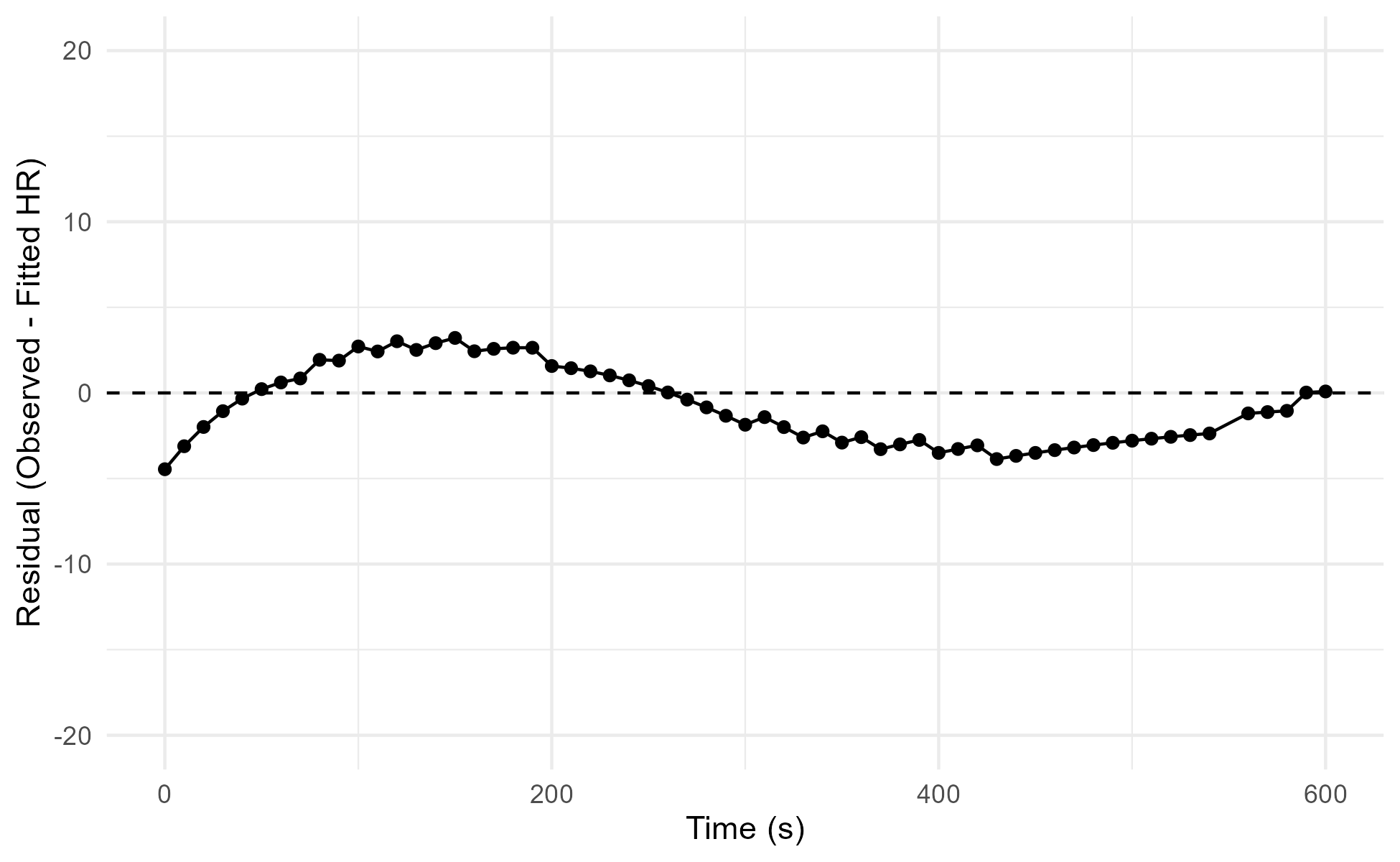


# Participant 7 – CVE trial

## Mono-exponential decay model fit


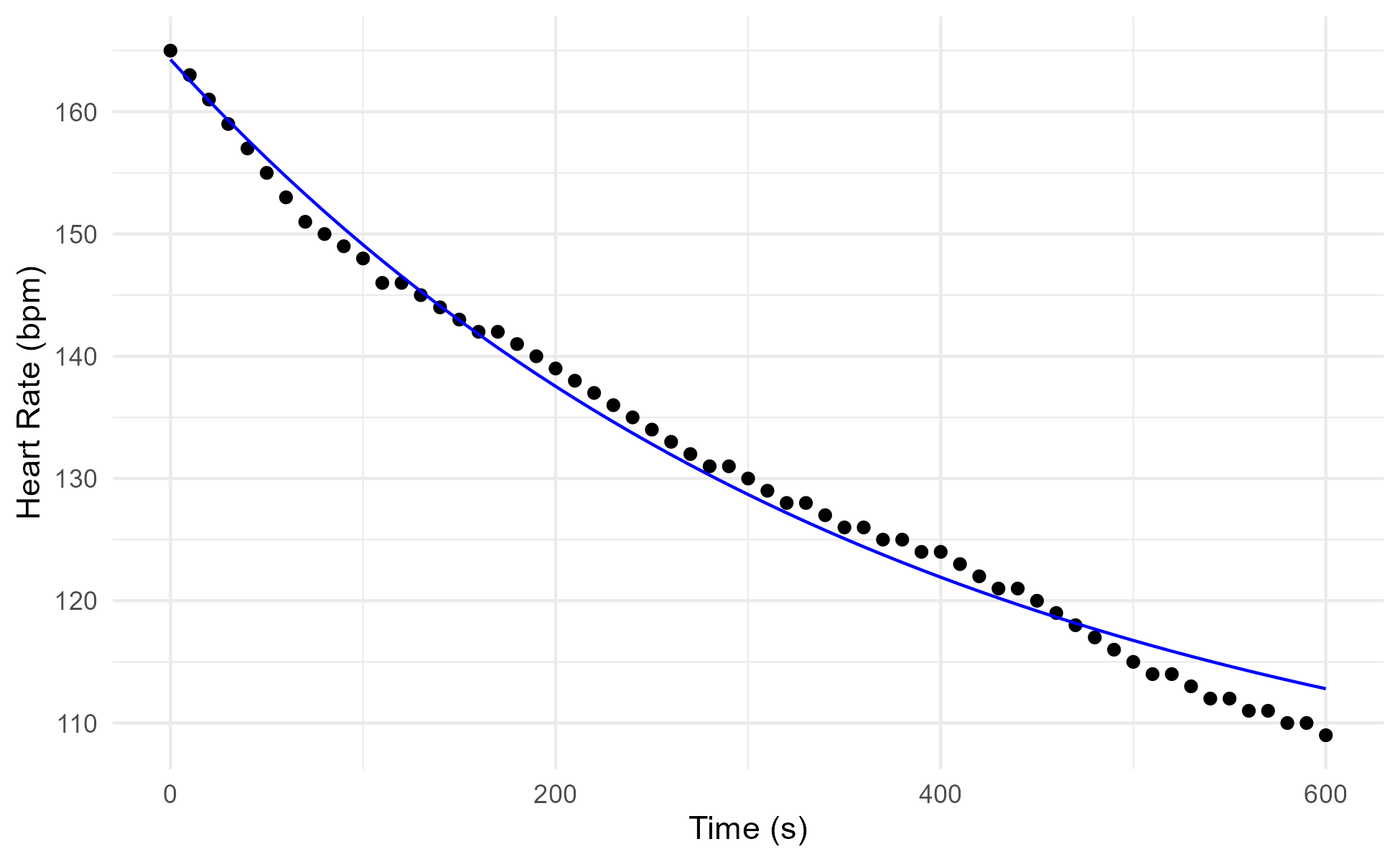


## Residuals of model fit


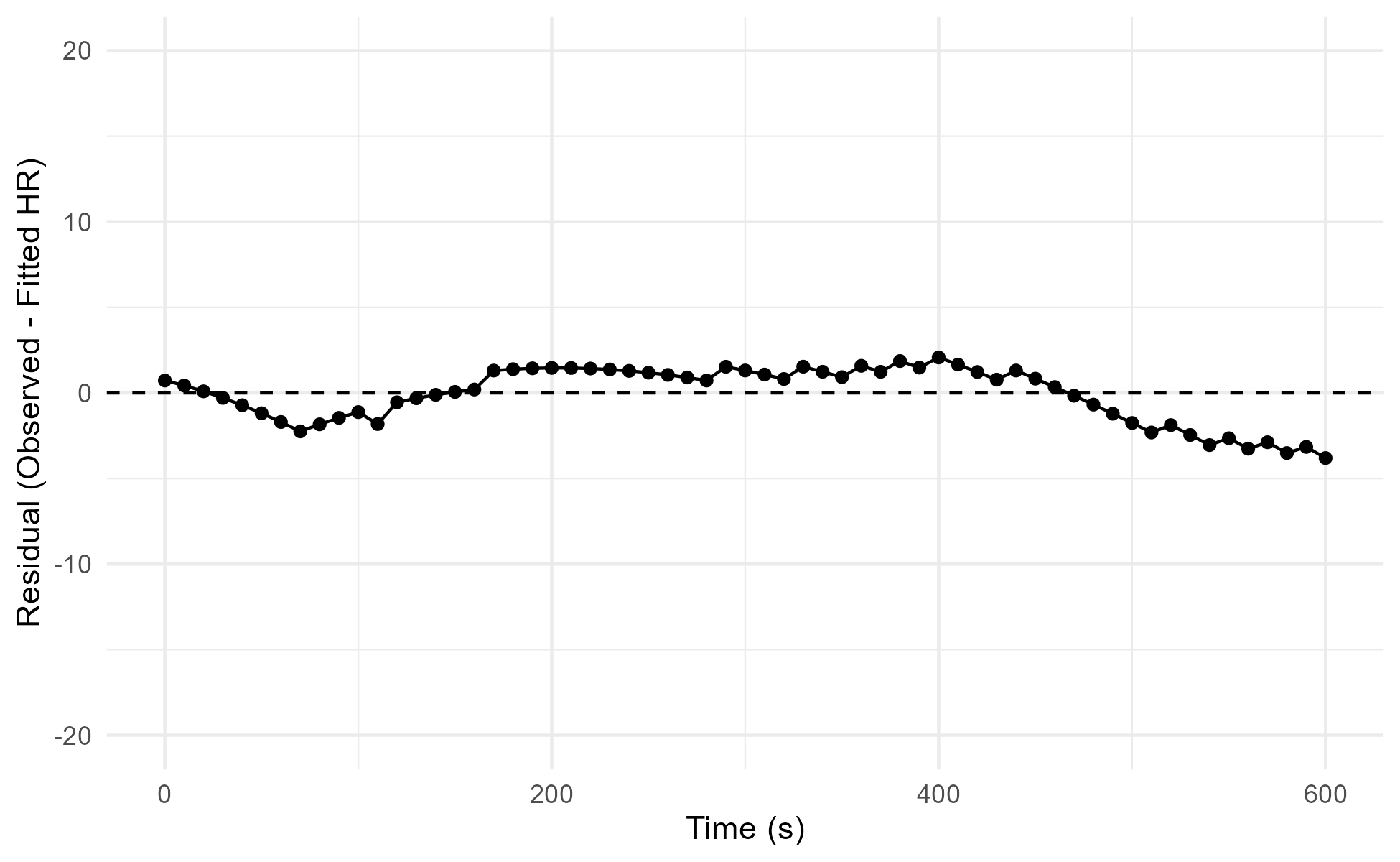


# Participant 8 – CME trial

## Mono-exponential decay model fit


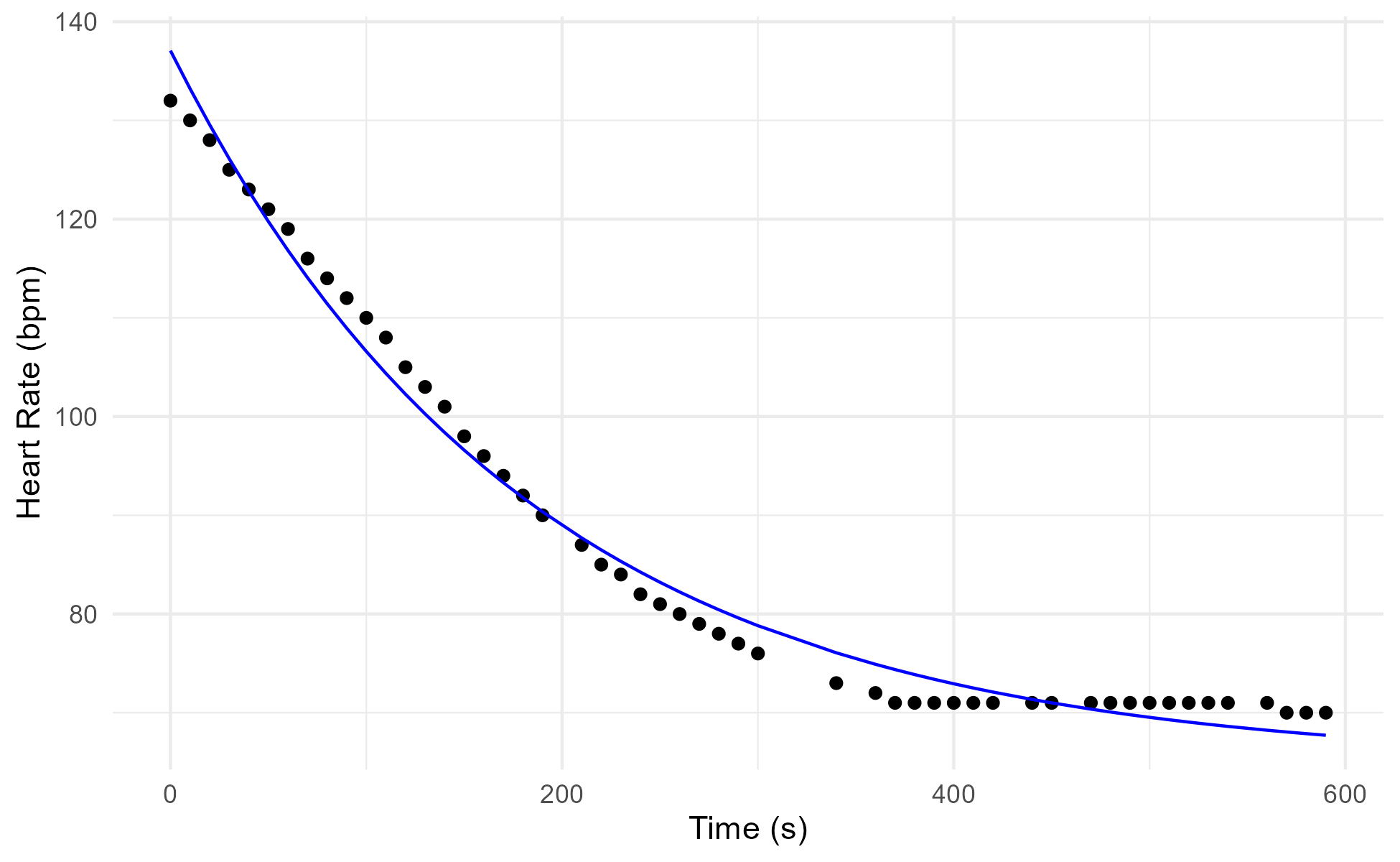


## Residuals of model fit


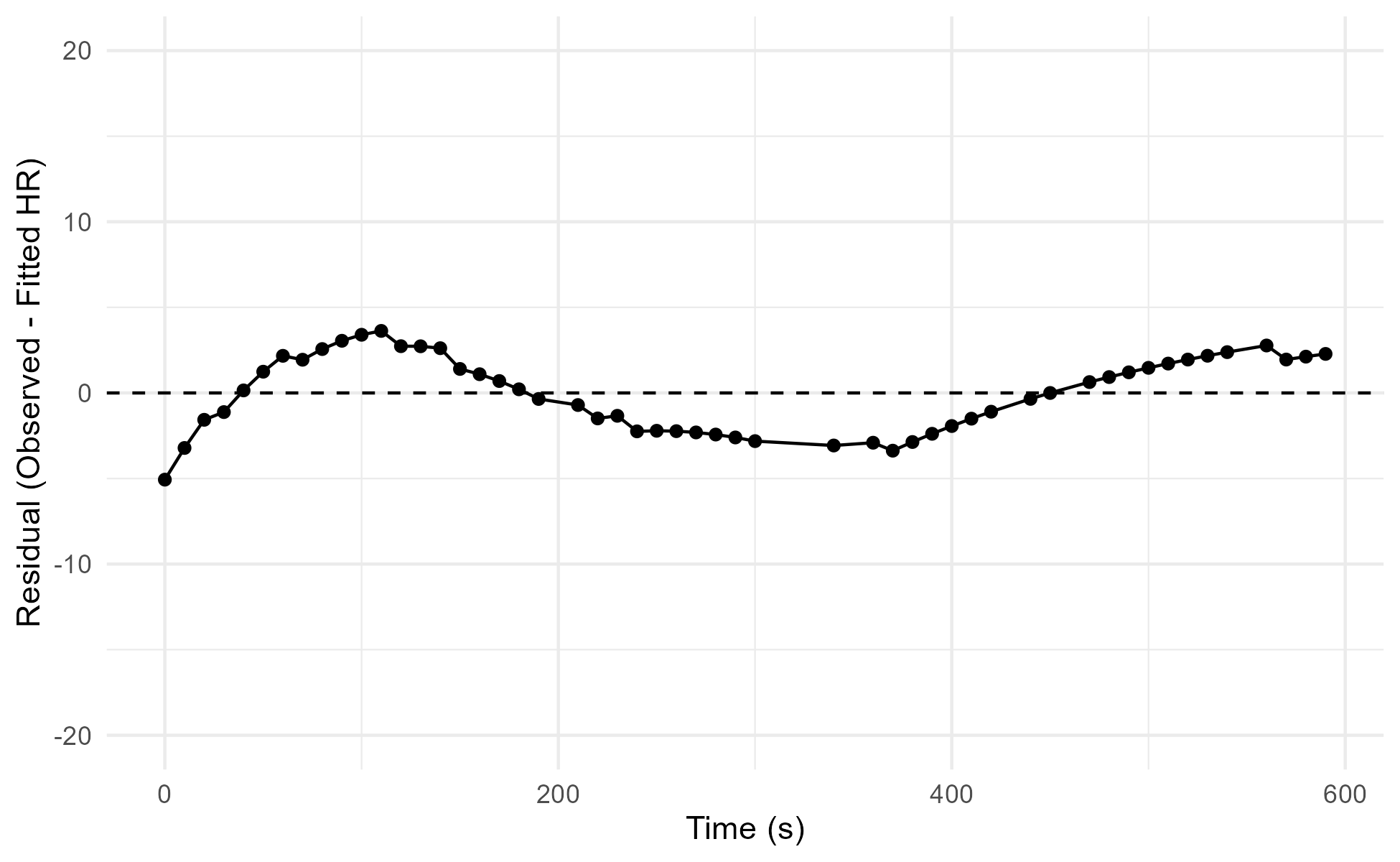


# Participant 8 – CVE trial

## Mono-exponential decay model fit


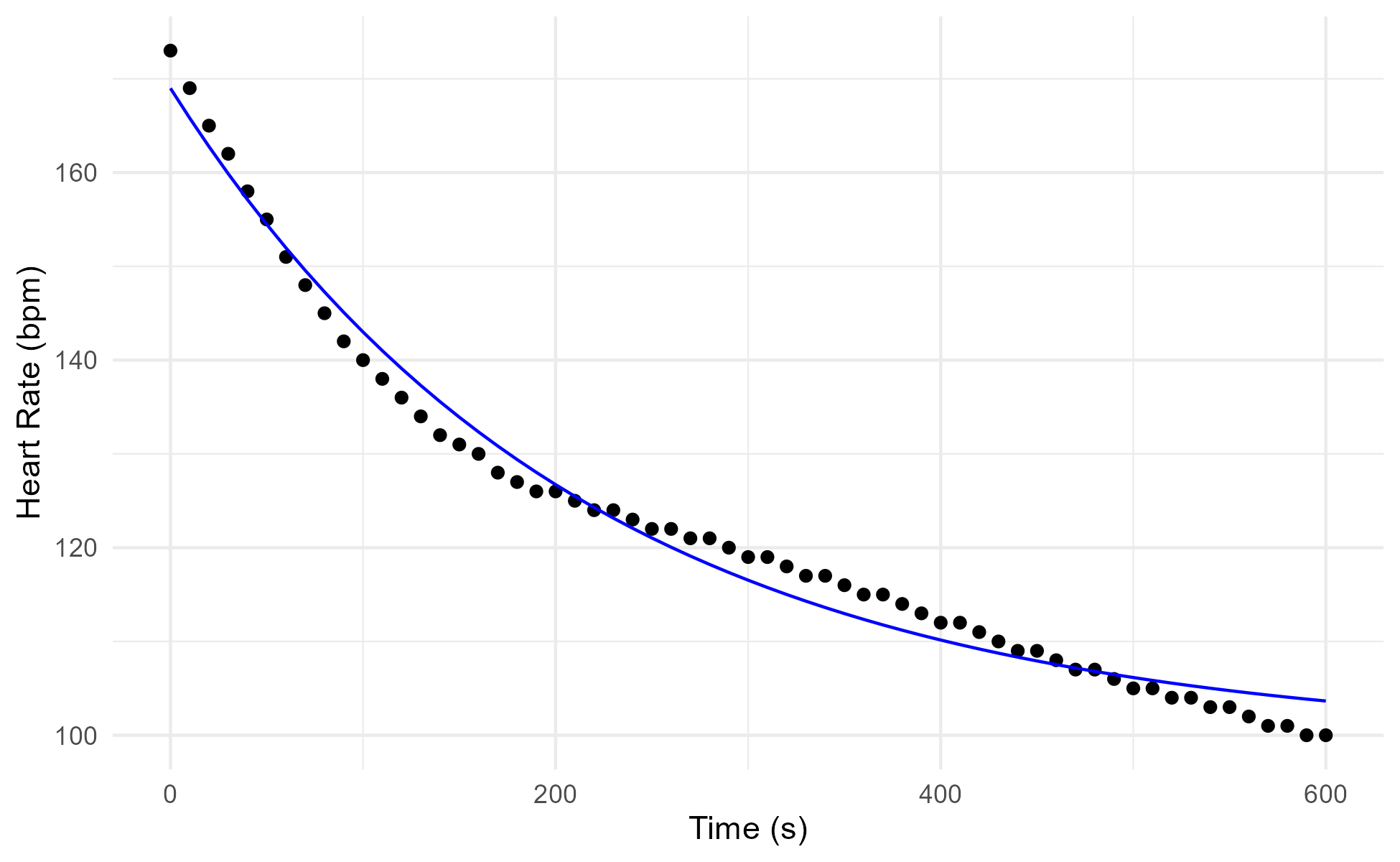


## Residuals of model fit


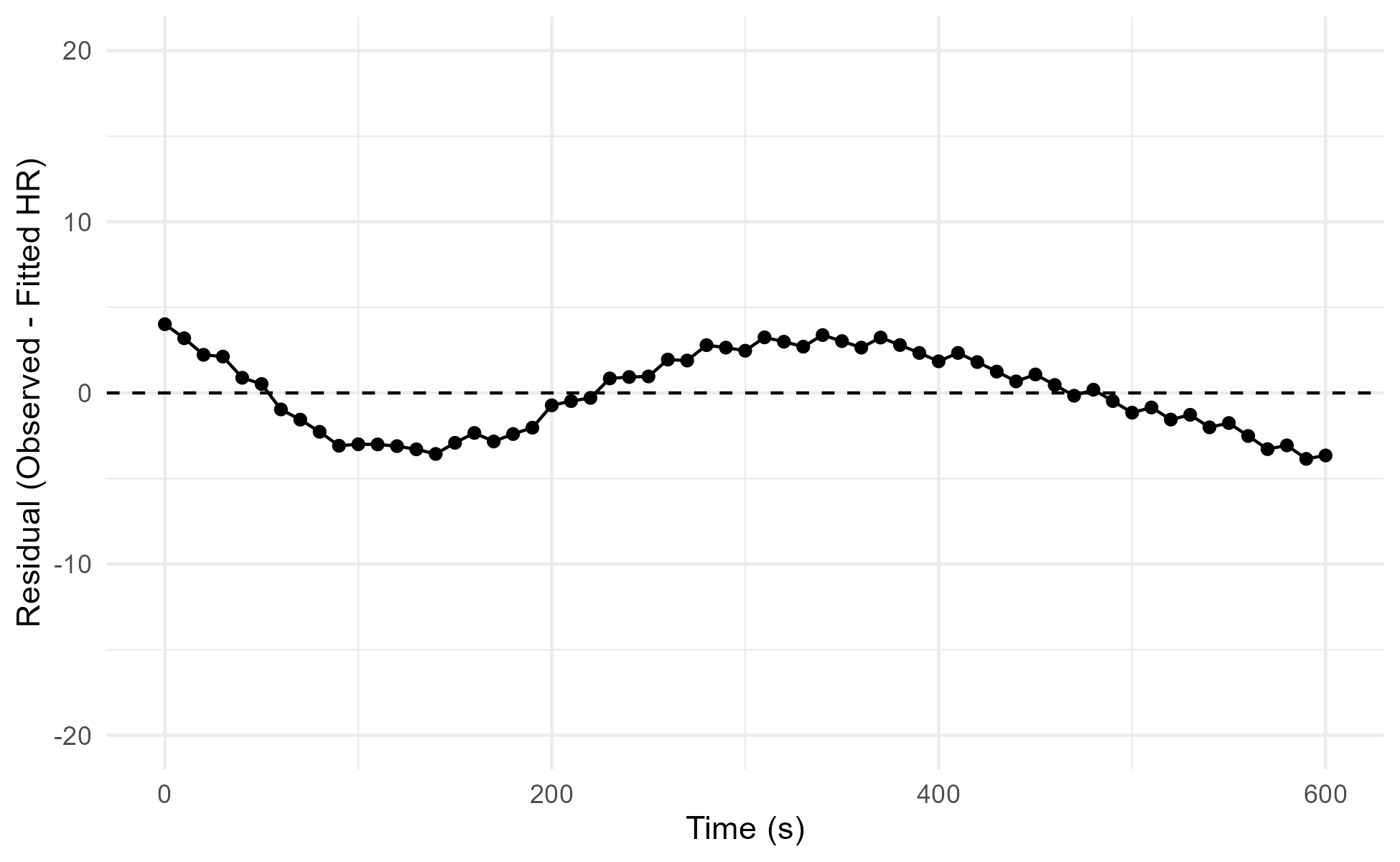


# Participant 9 – CME trial

## Mono-exponential decay model fit


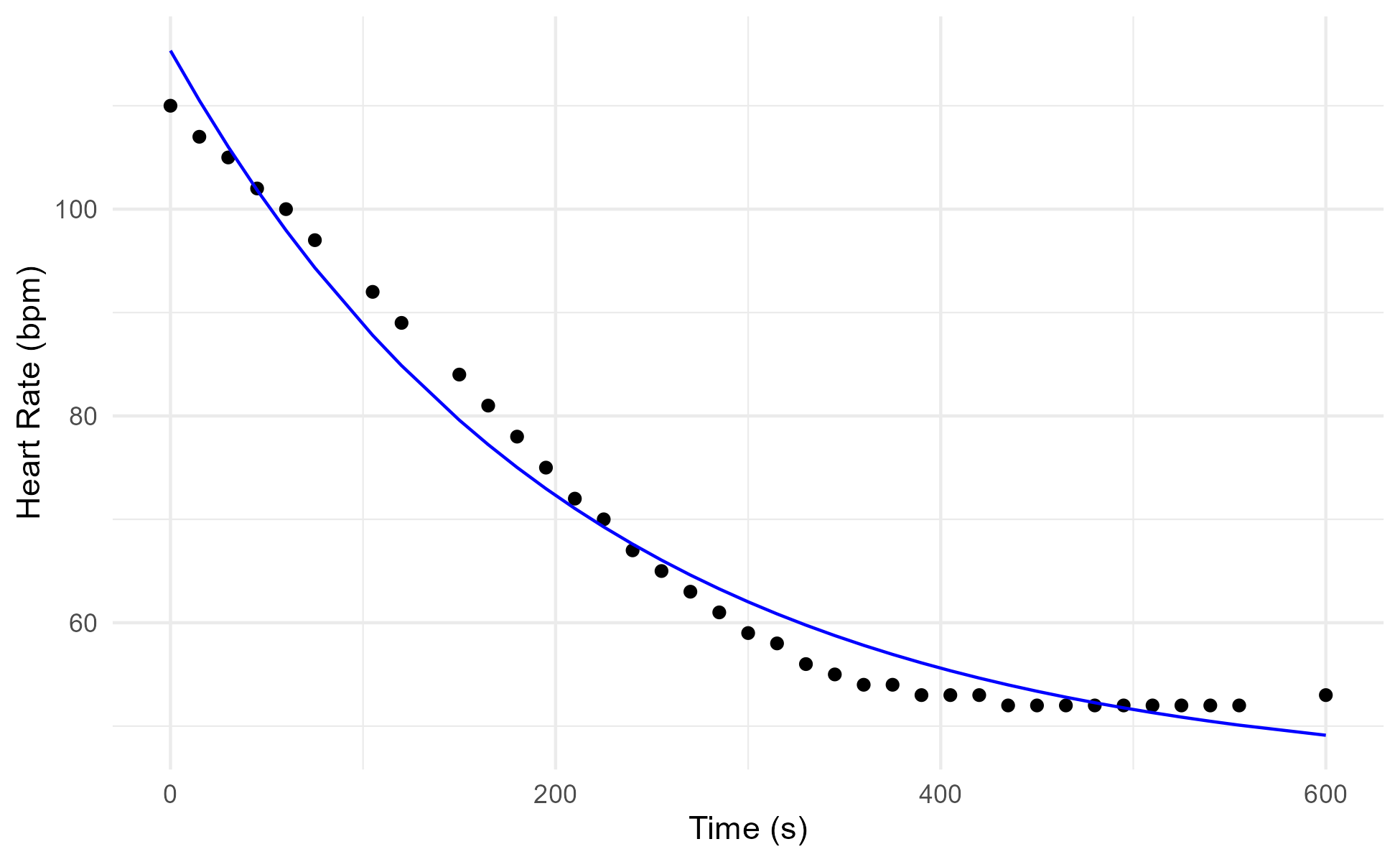


## Residuals of model fit


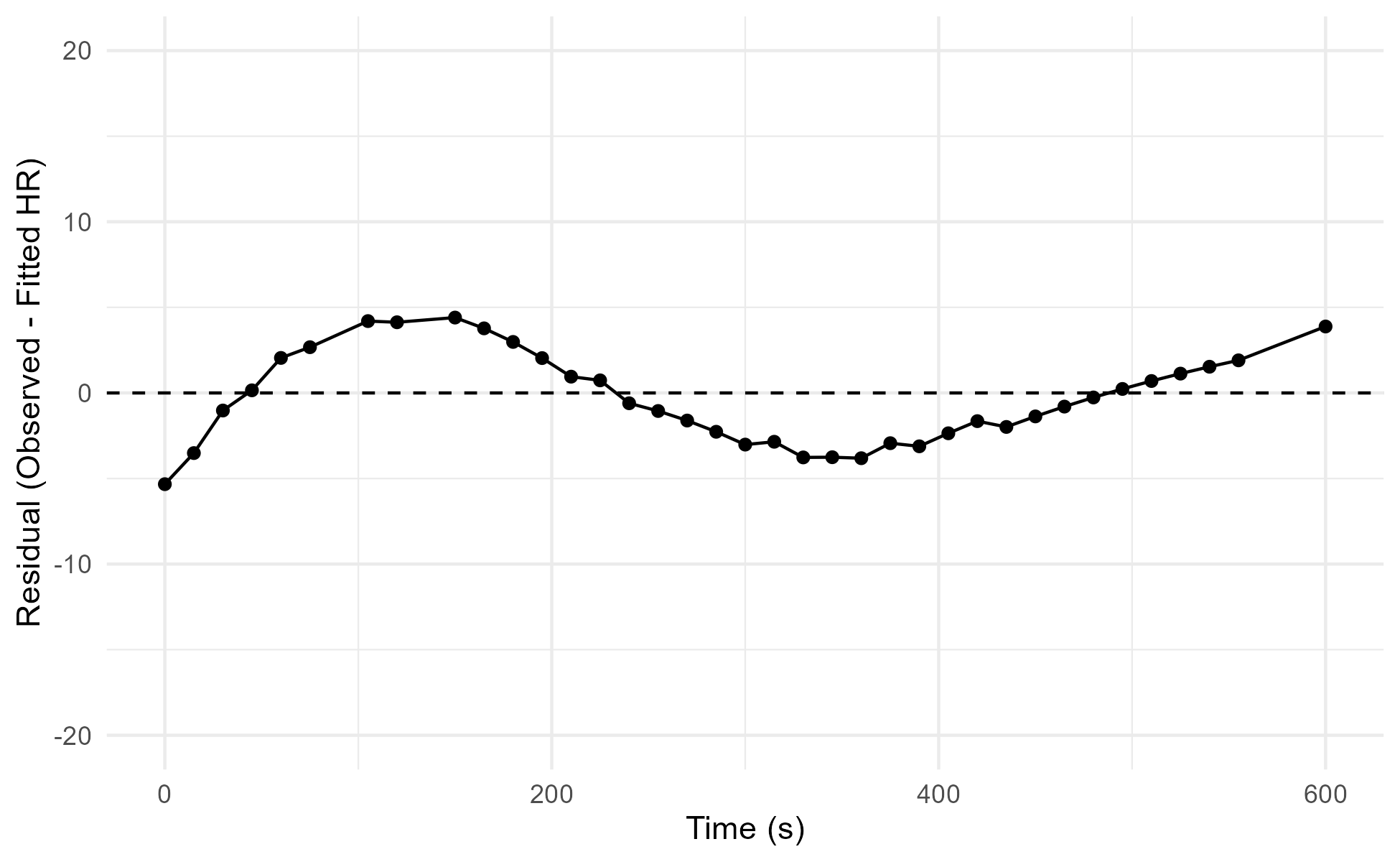


# Participant 9 – CVE trial

## Mono-exponential decay model fit


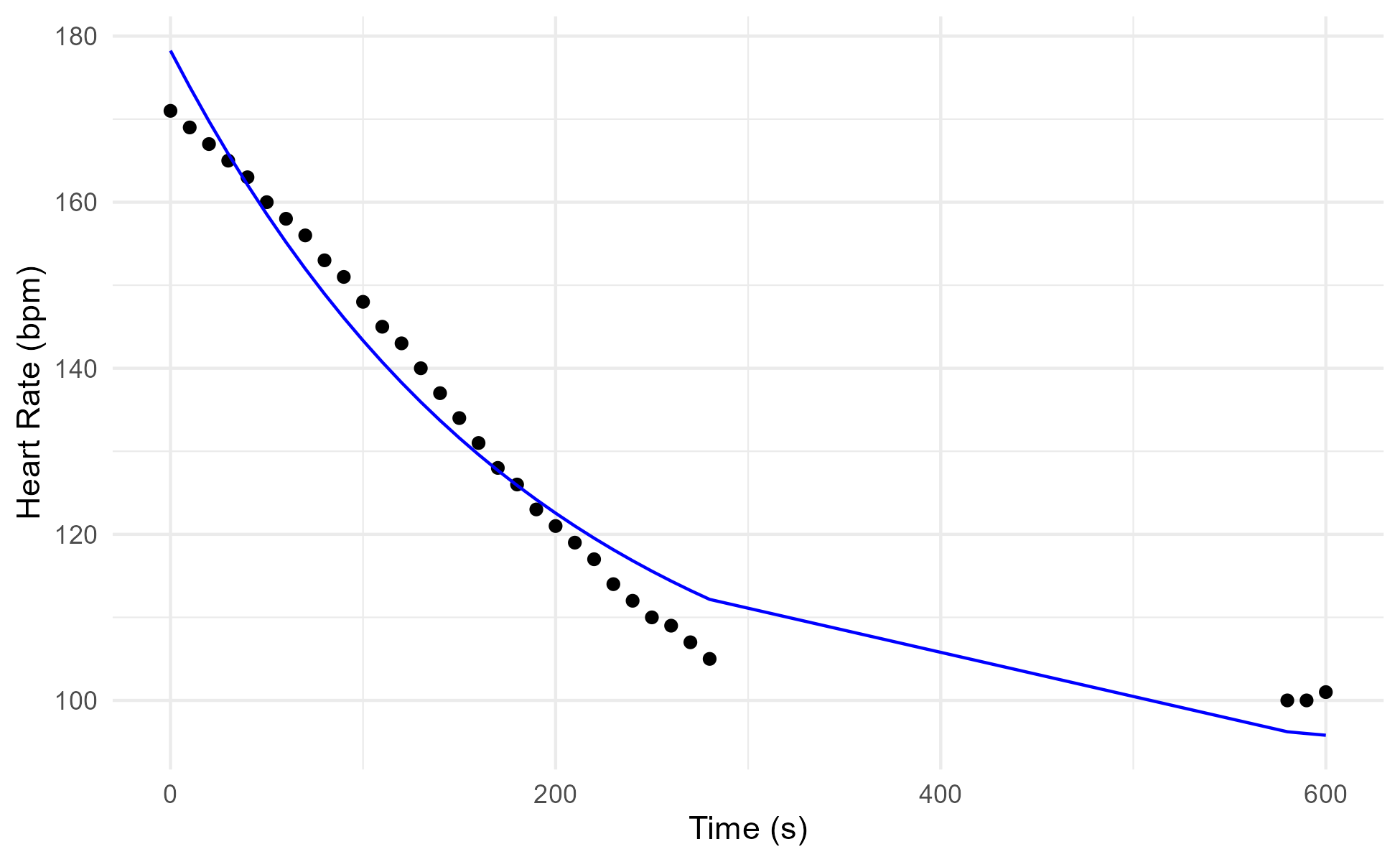


## Residuals of model fit


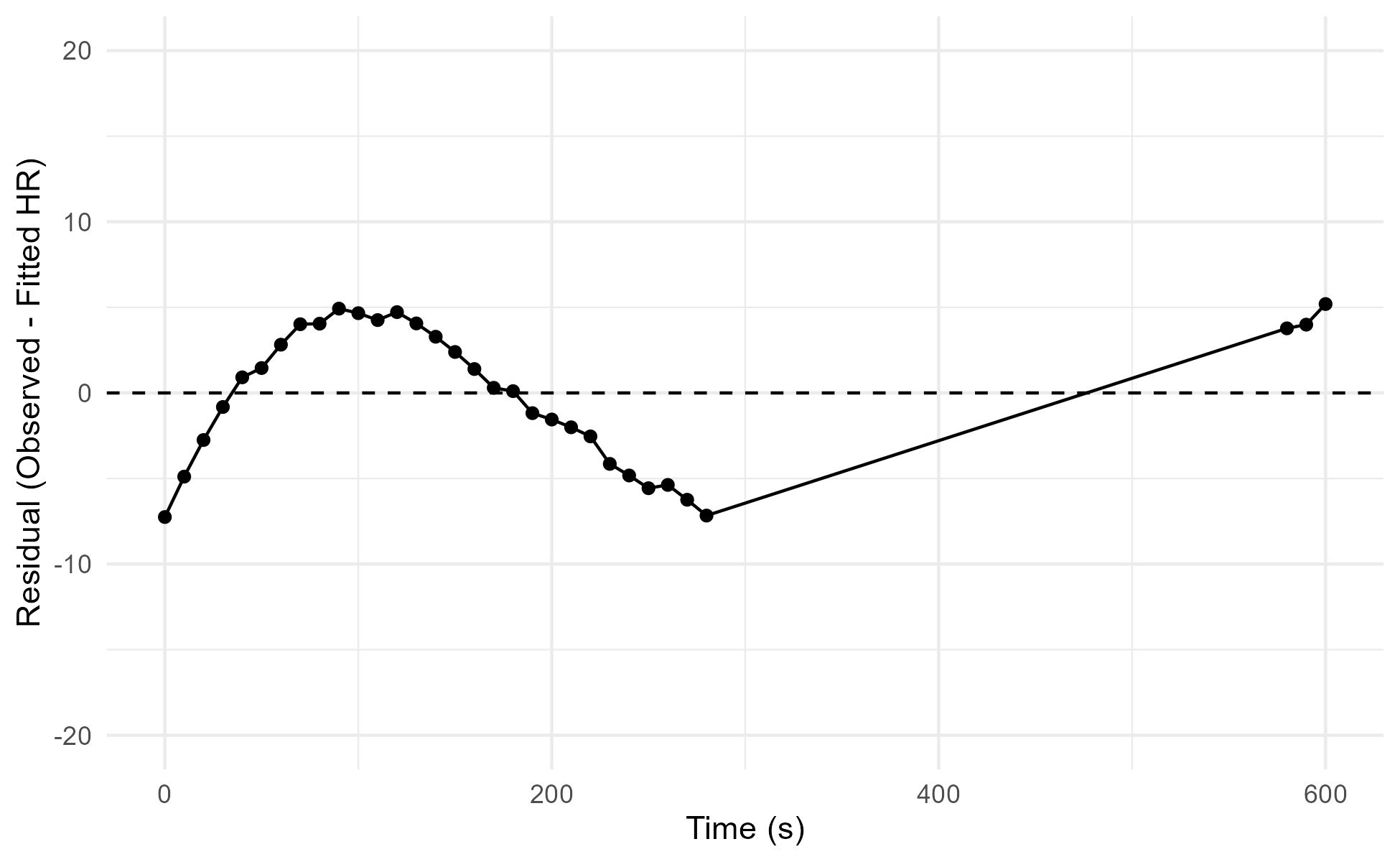


# Participant 10 – CVE trial

## Mono-exponential decay model fit


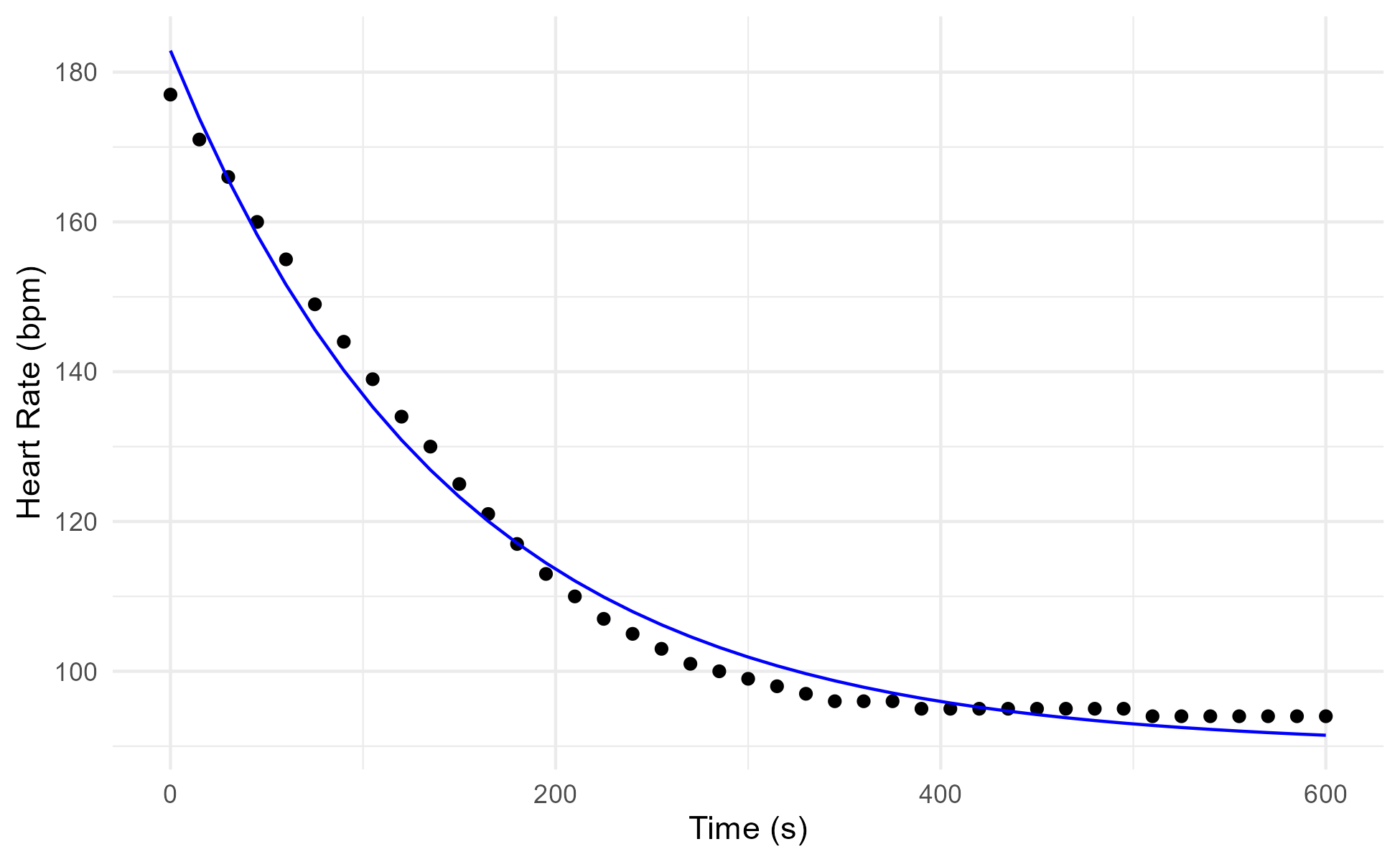


## Residuals of model fit


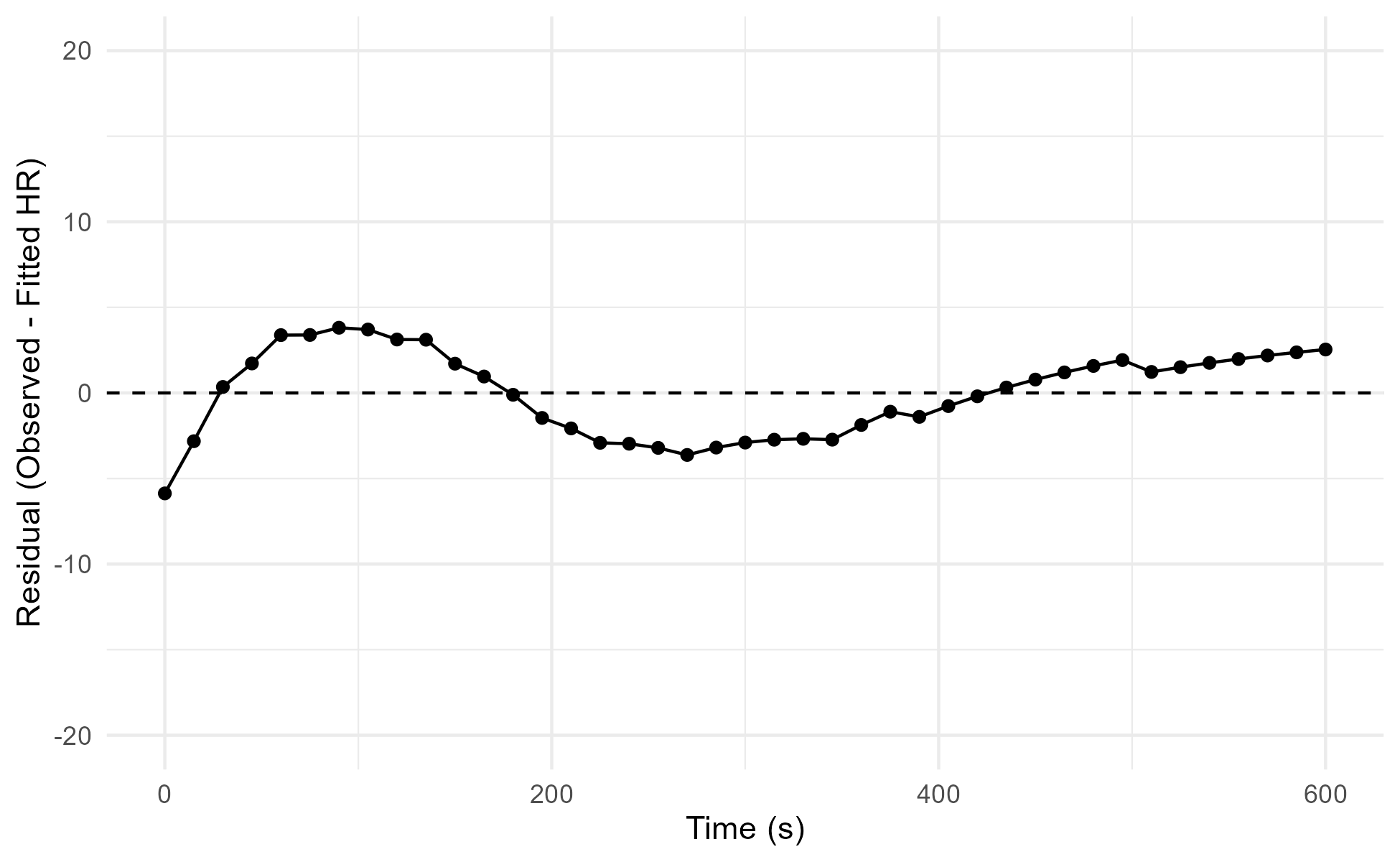


# Participant 11 – CME trial

## Mono-exponential decay model fit


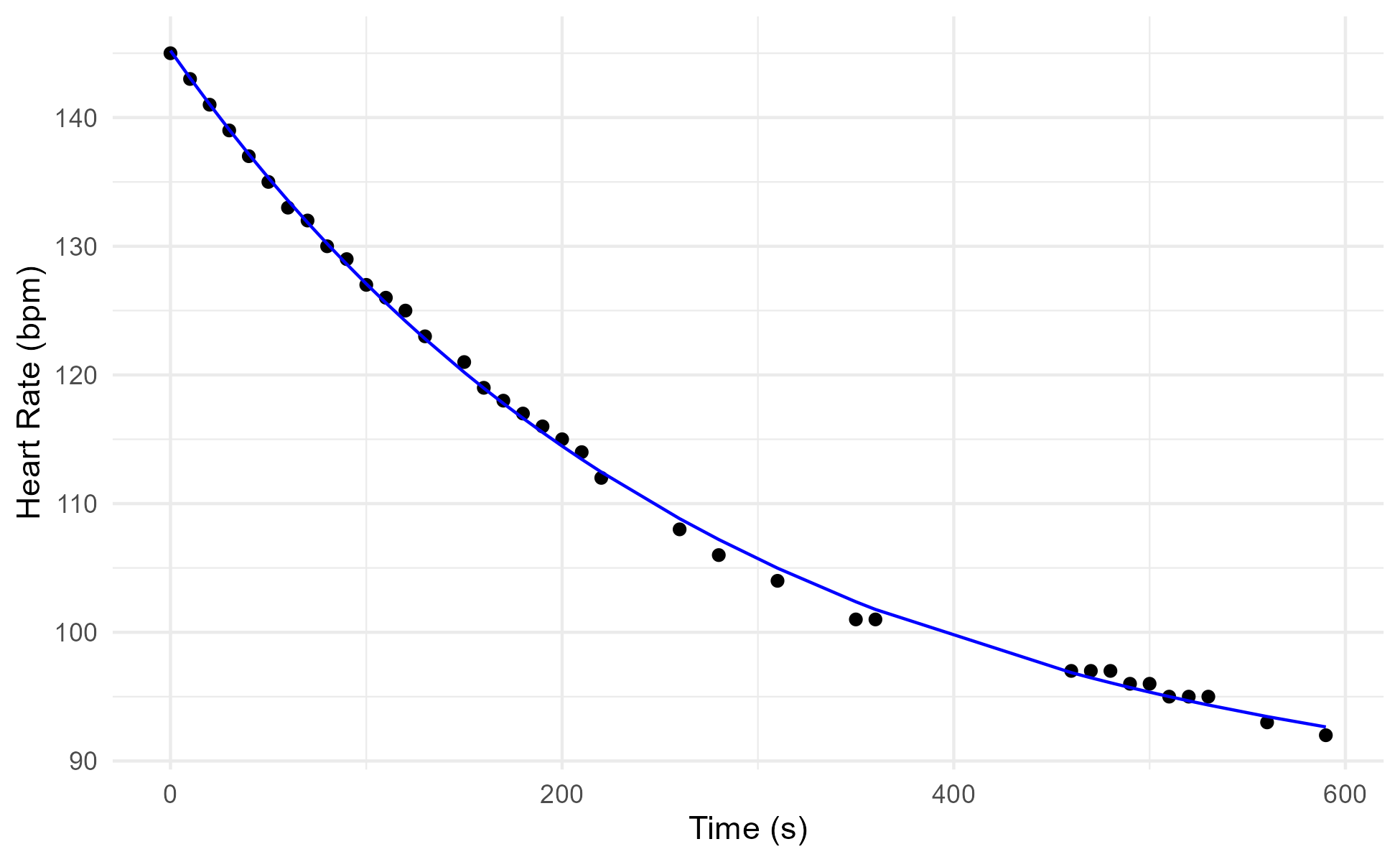


## Residuals of model fit


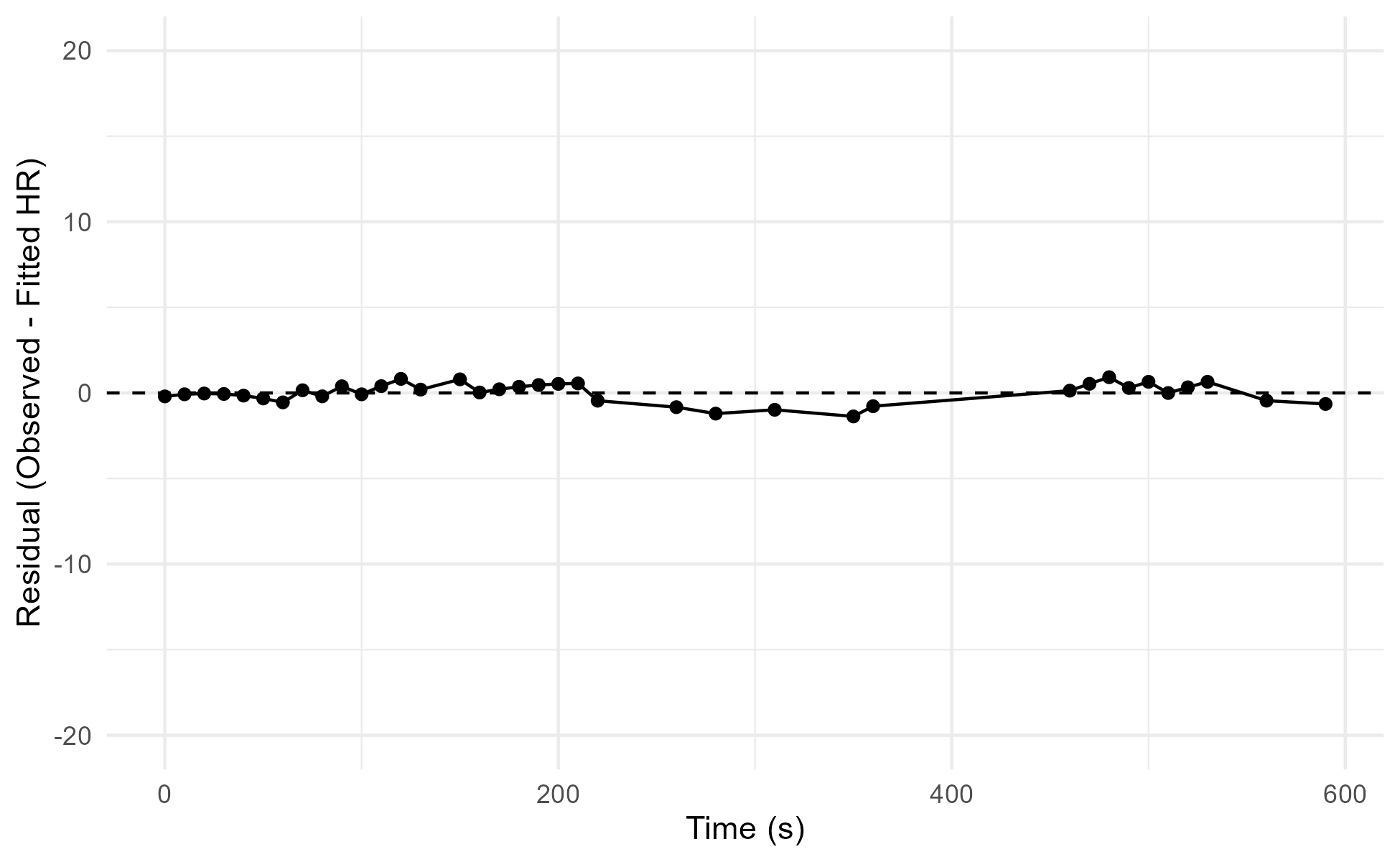


# Participant 11 – CVE trial

## Mono-exponential decay model fit


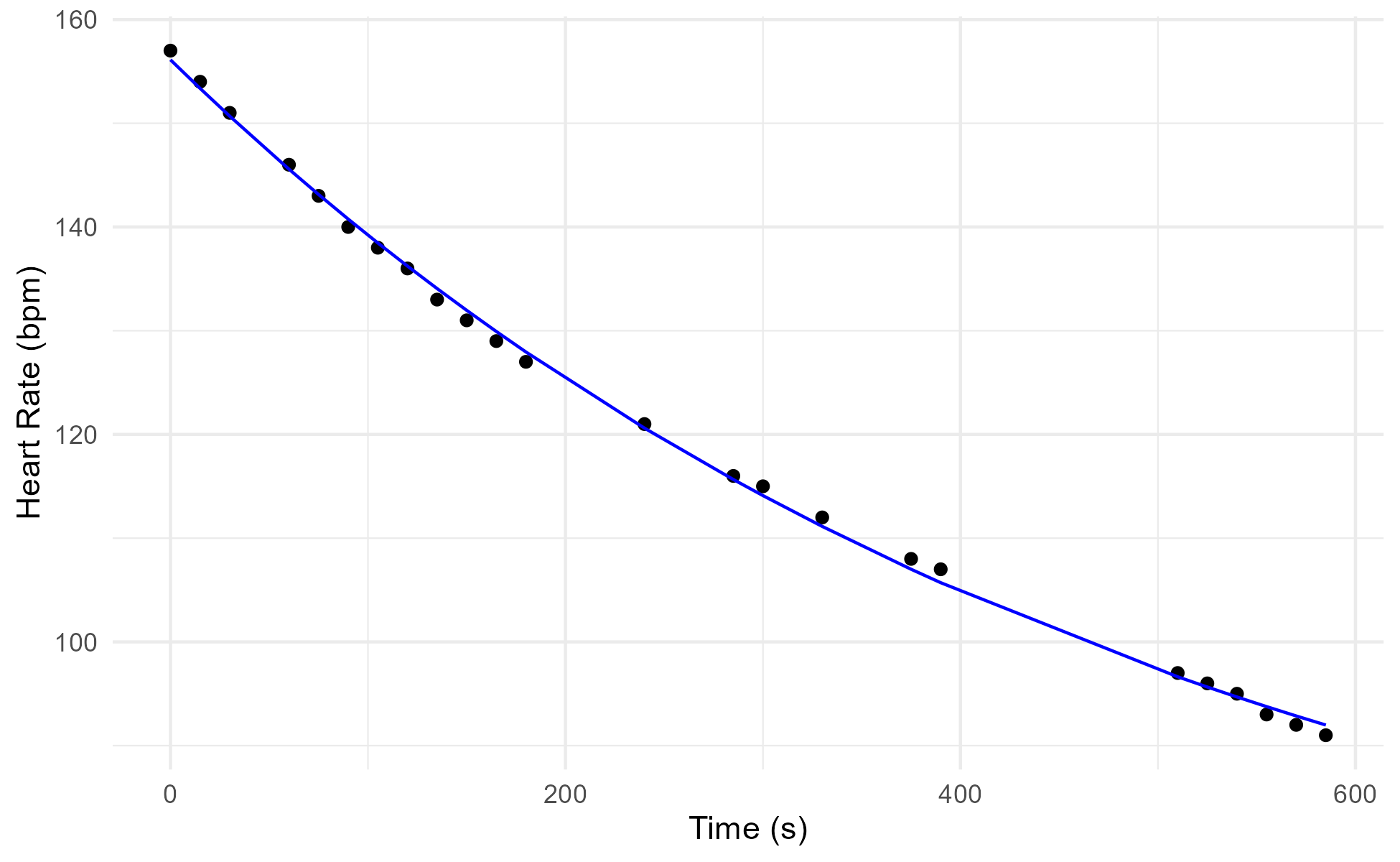


## Residuals of model fit


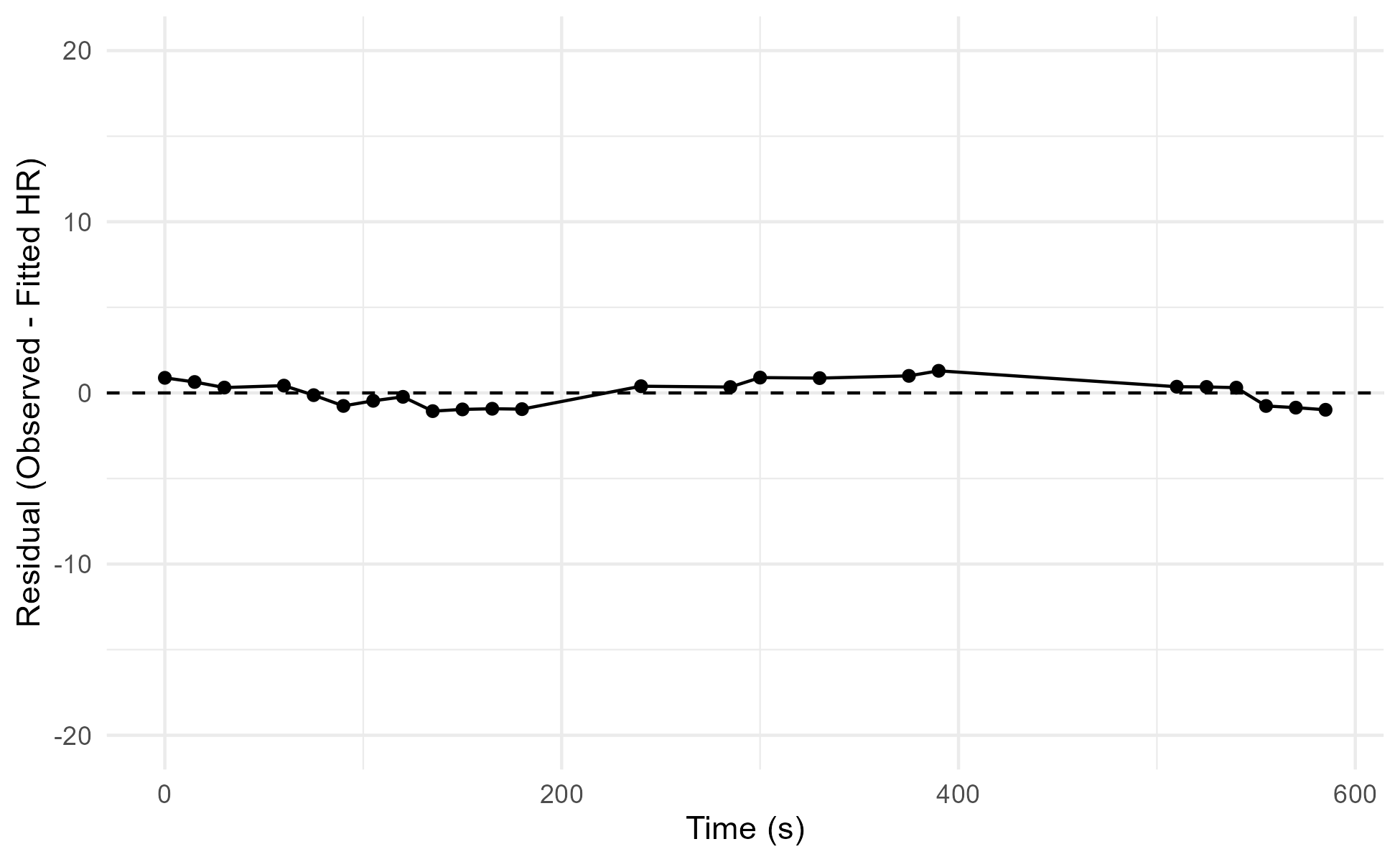


# Participant 12 – CME trial

## Mono-exponential decay model fit


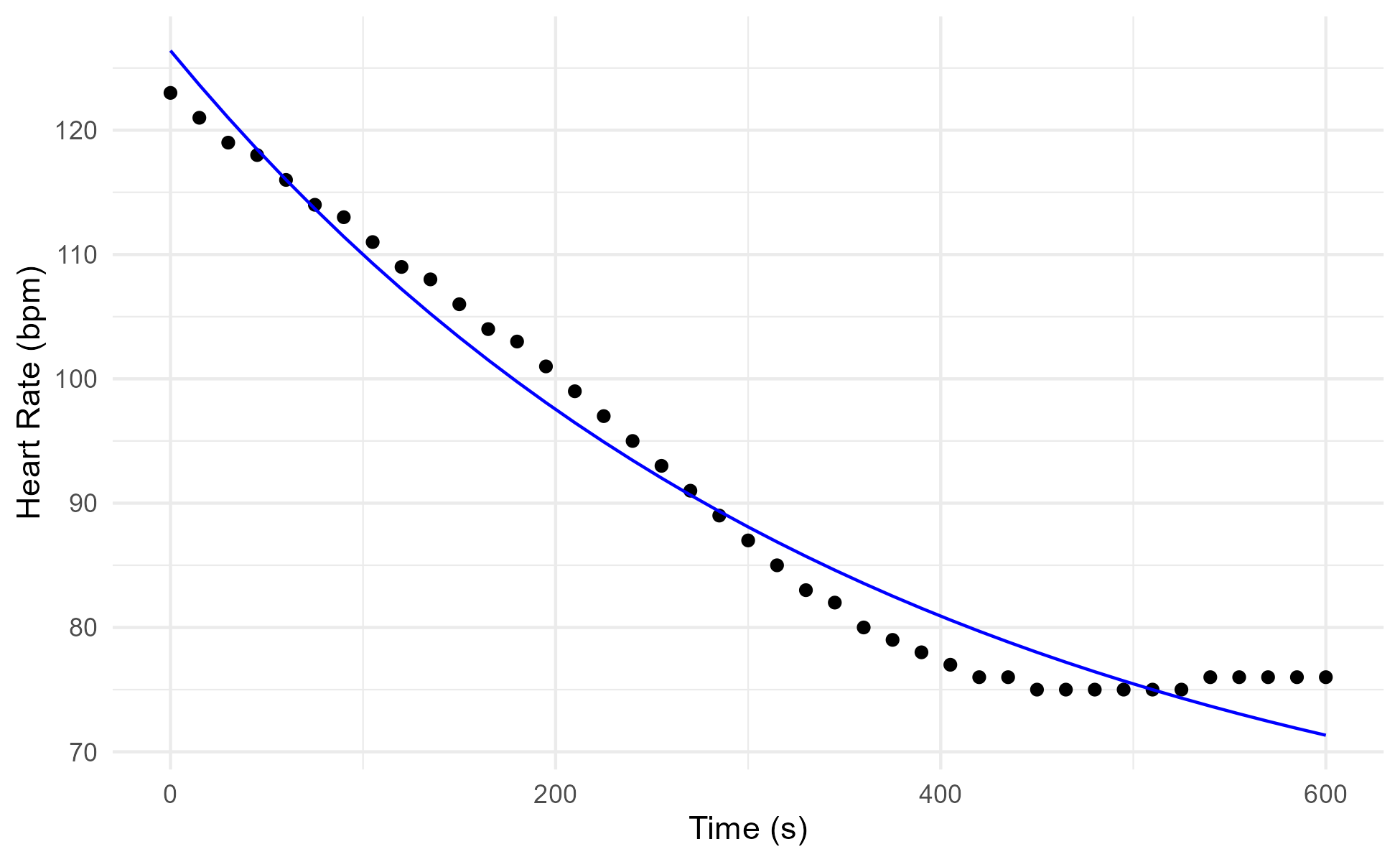


## Residuals of model fit


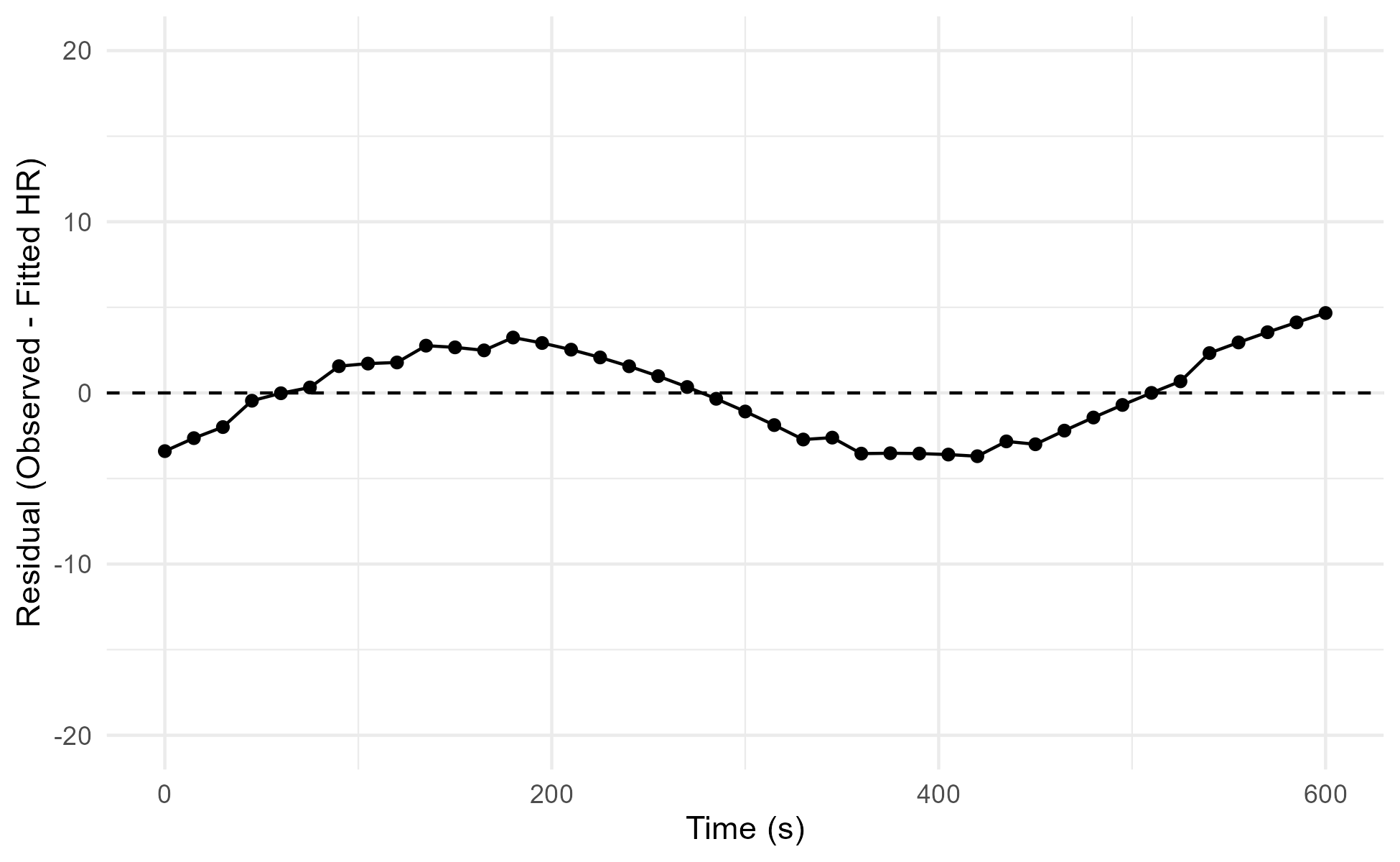


# Participant 12 – CVE trial

## Mono-exponential decay model fit


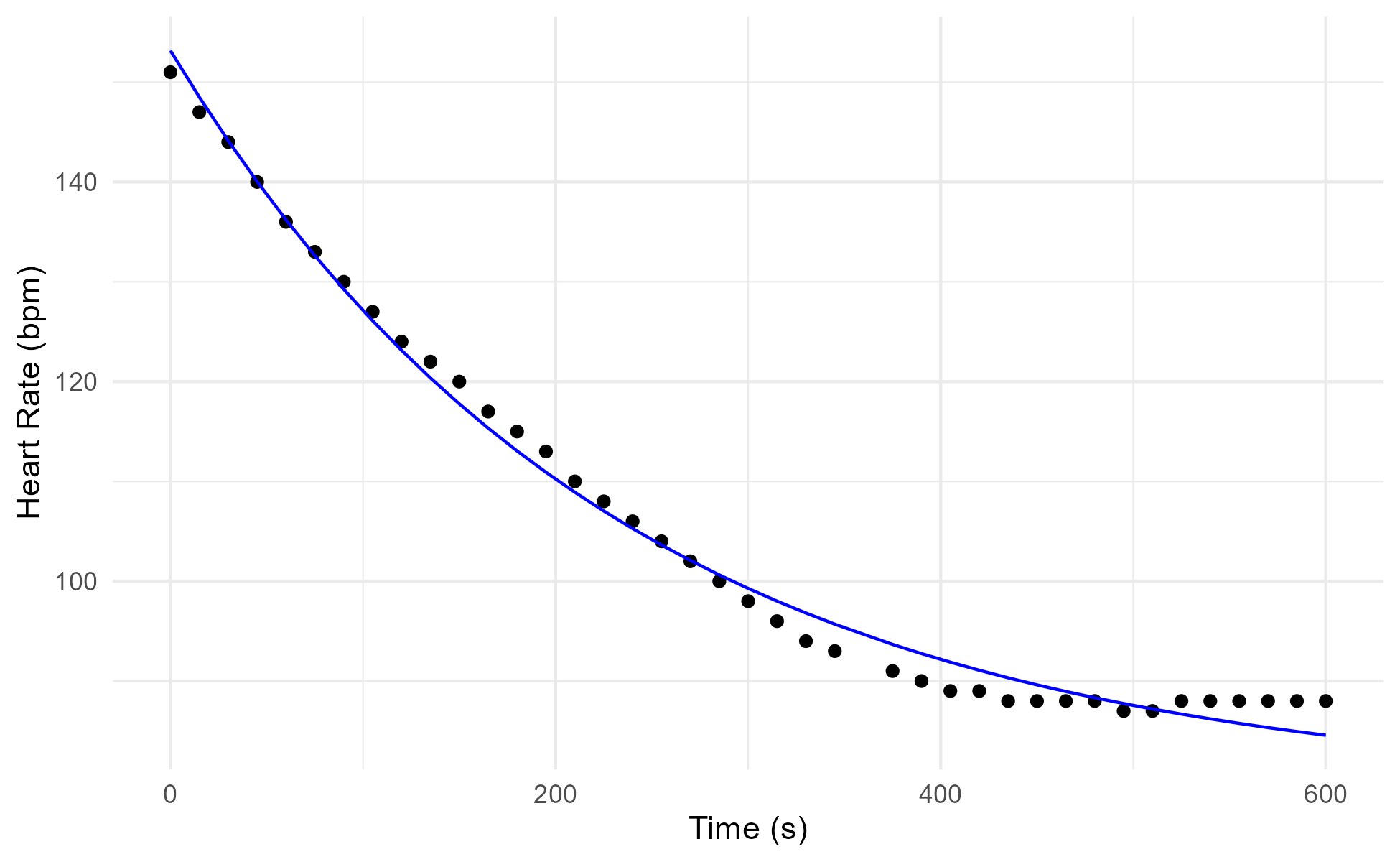


## Residuals of model fit


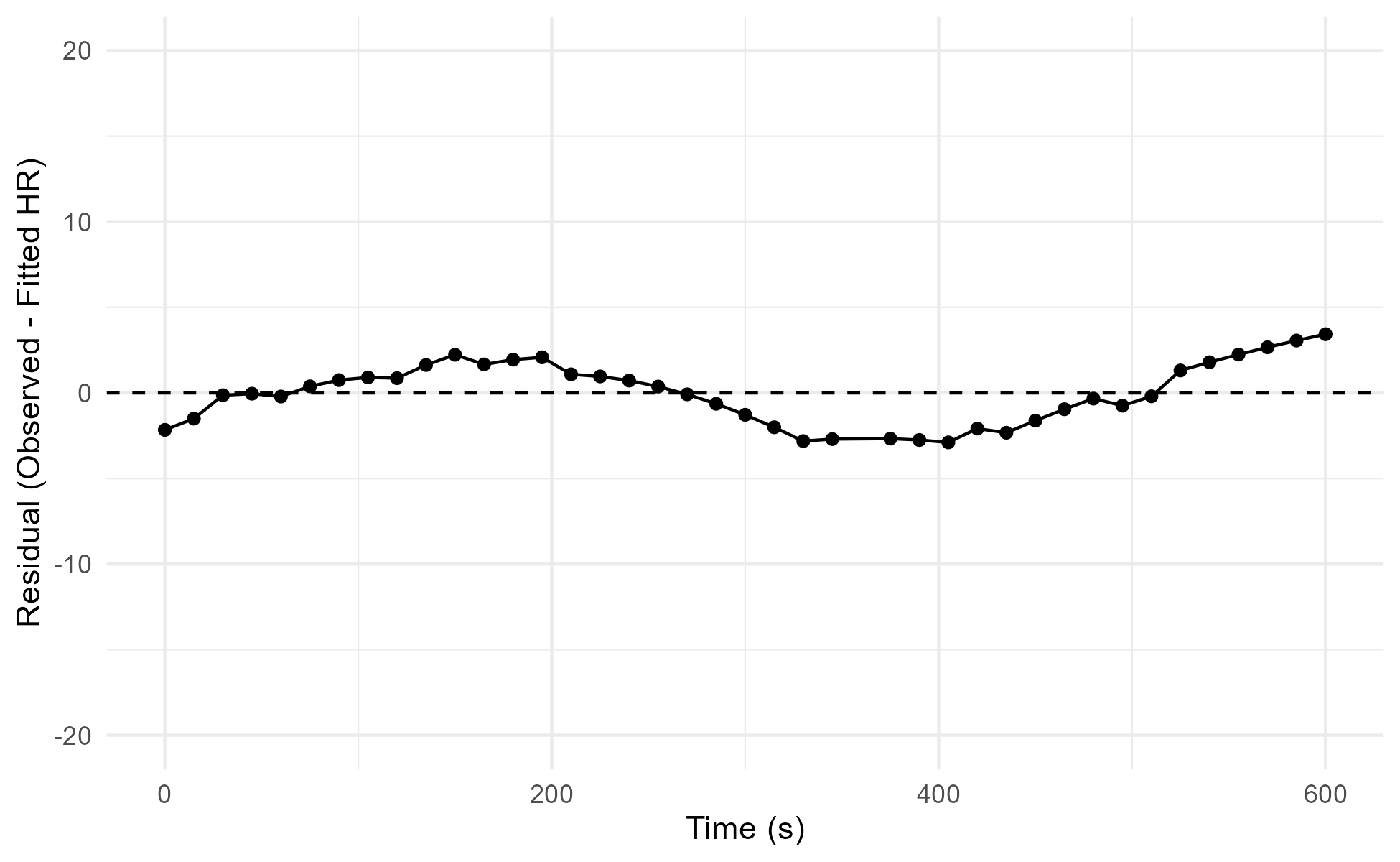


# Participant 13 – CME trial

## Mono-exponential decay model fit


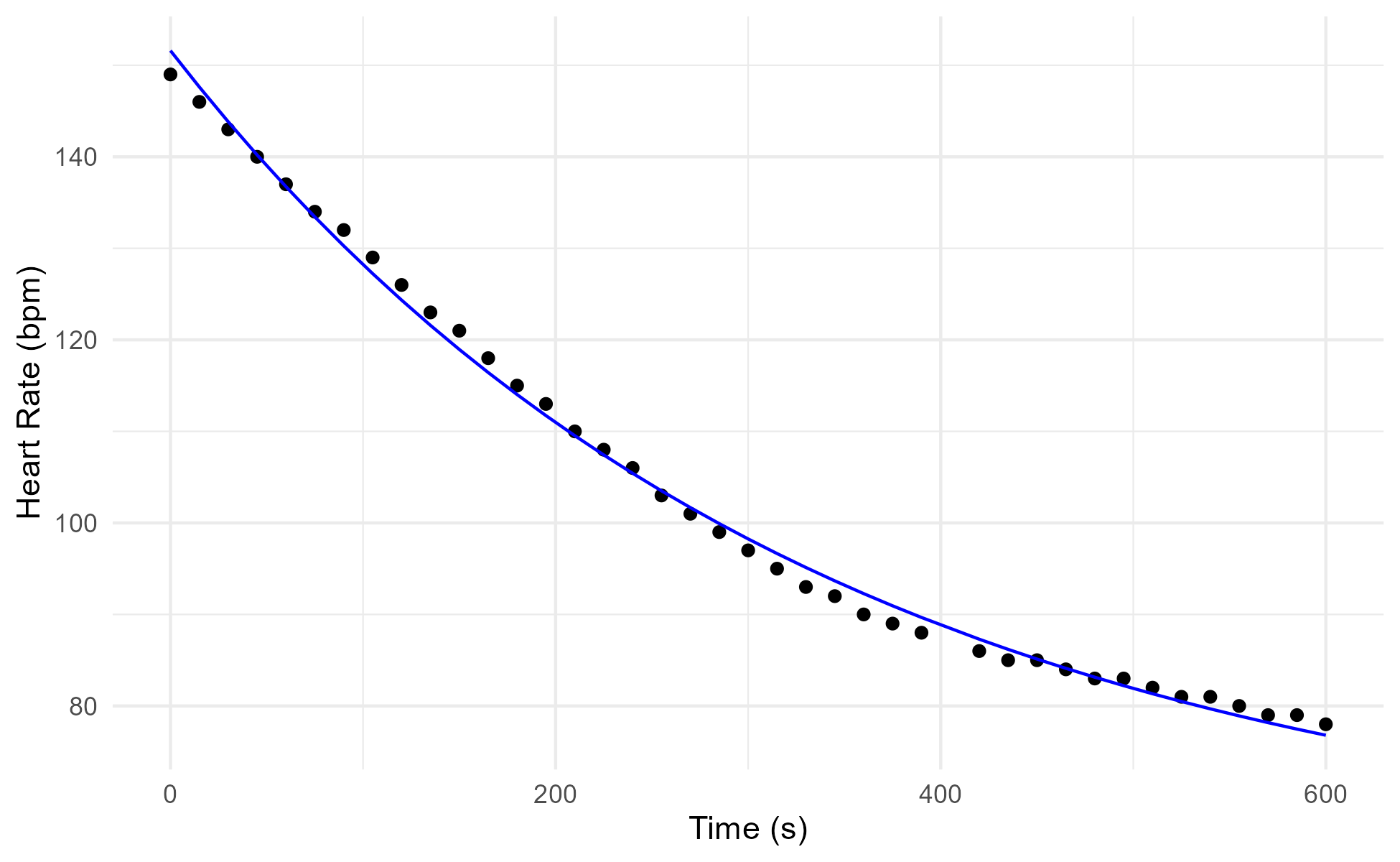


## Residuals of model fit


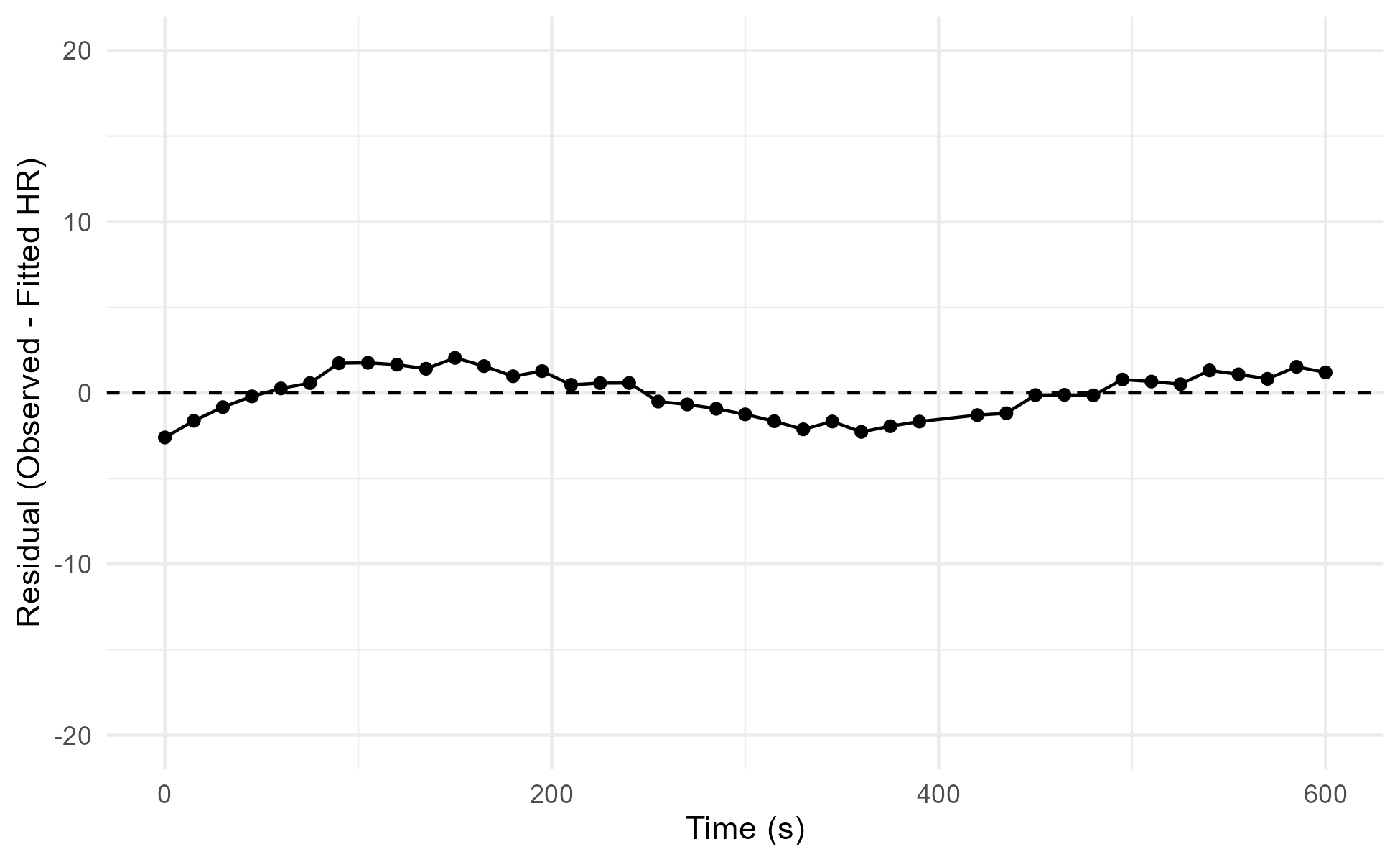


# Participant 13 – CVE trial

## Mono-exponential decay model fit


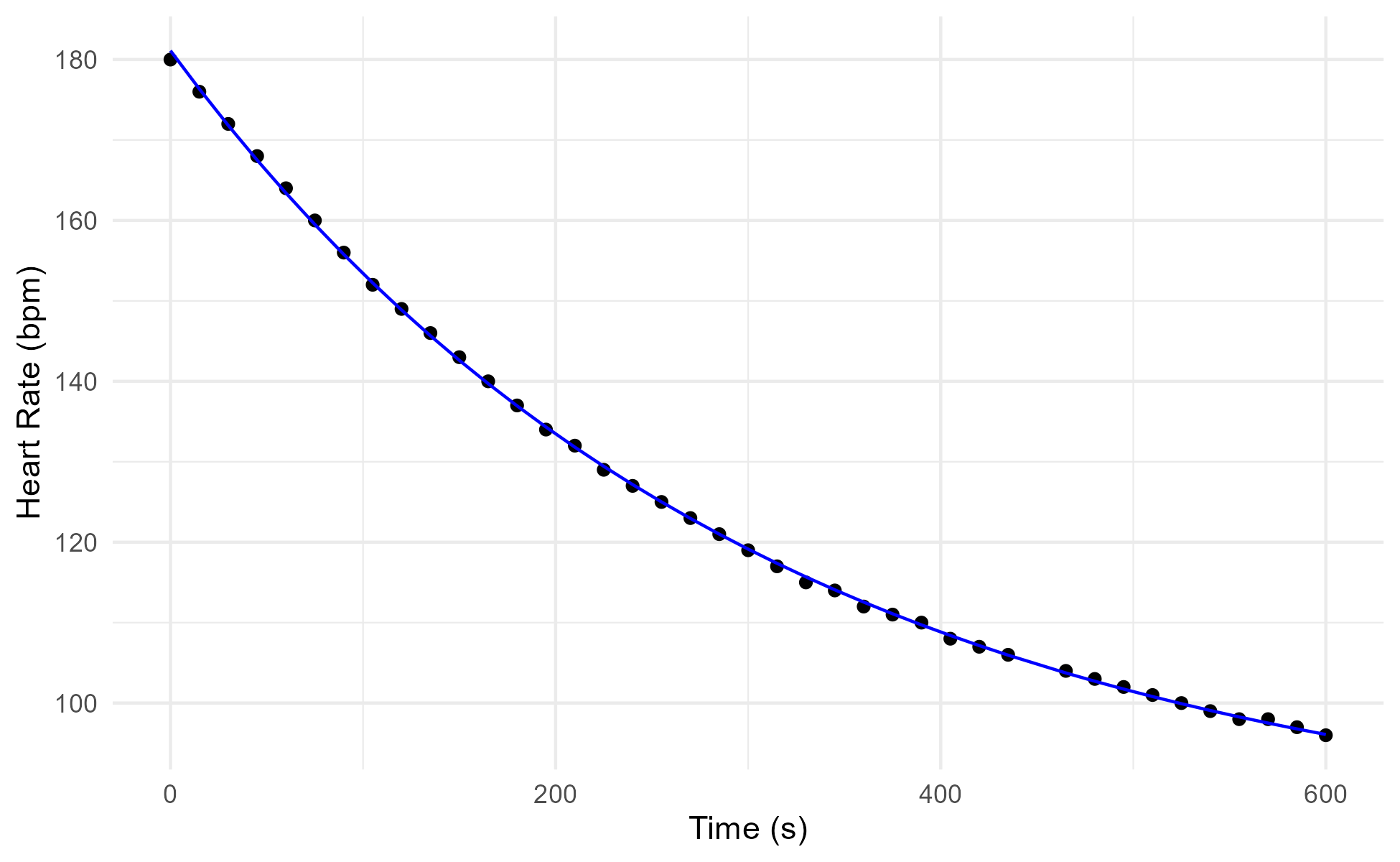


## Residuals of model fit


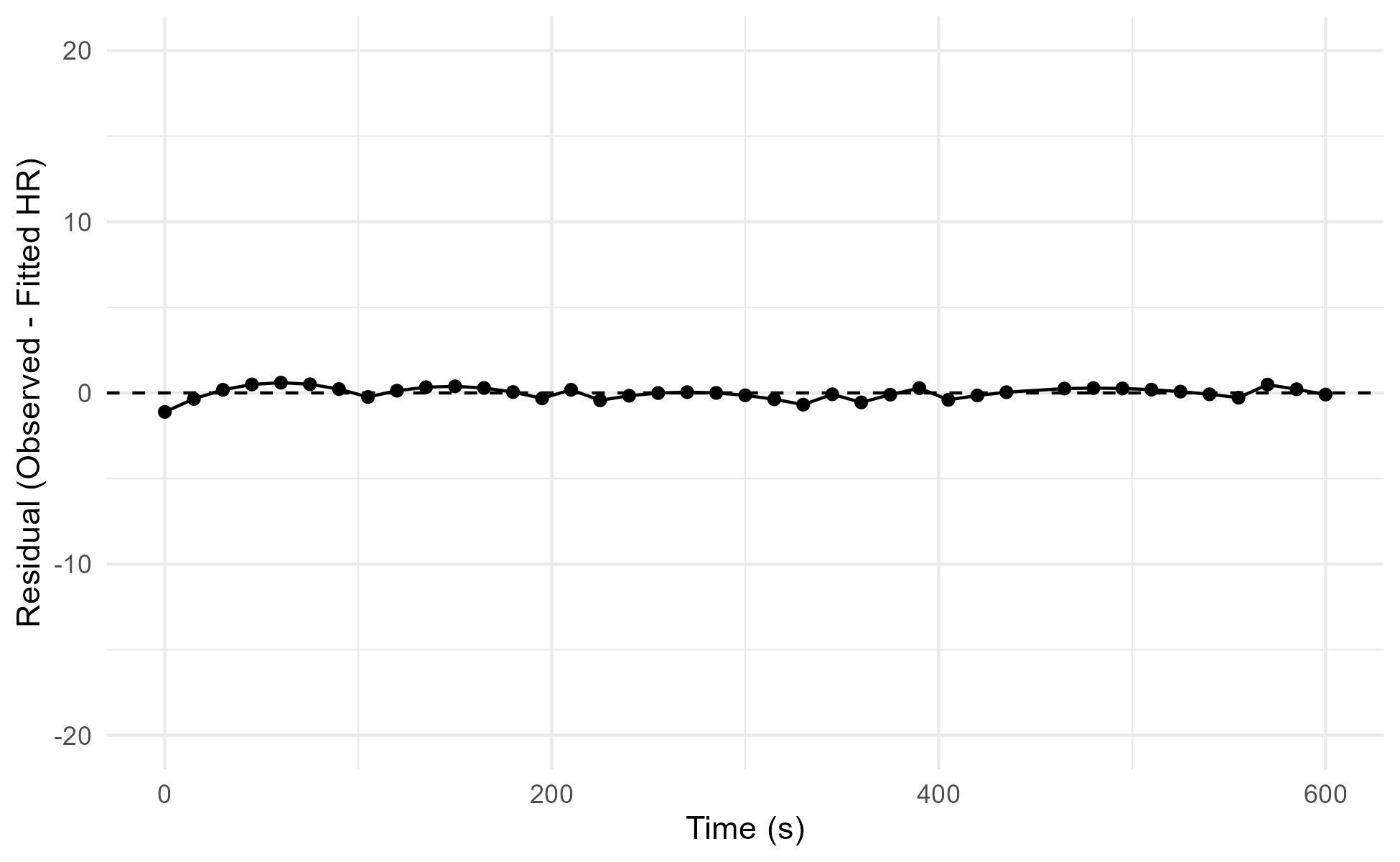


# Participant 14 – CME trial

## Mono-exponential decay model fit


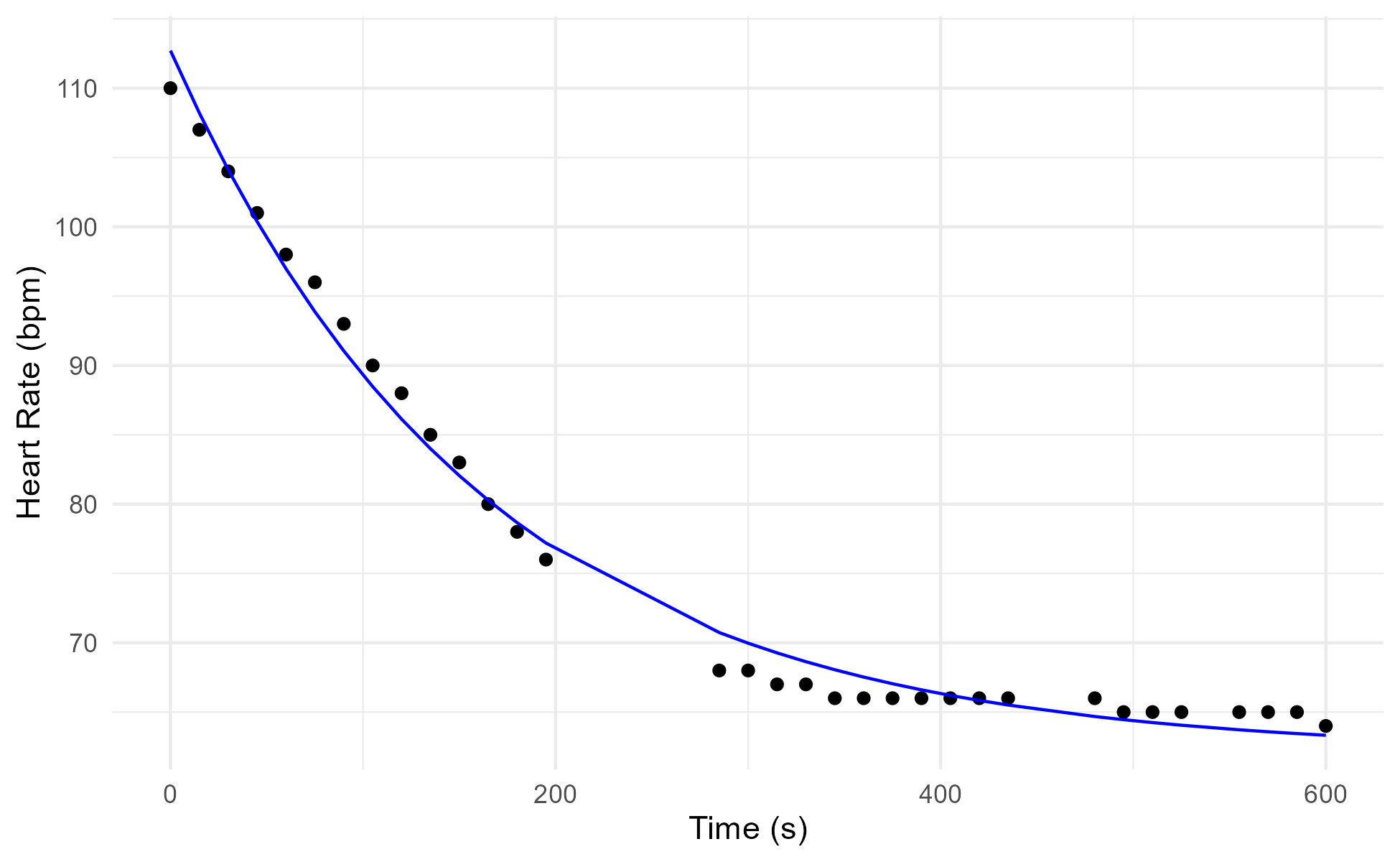


## Residuals of model fit


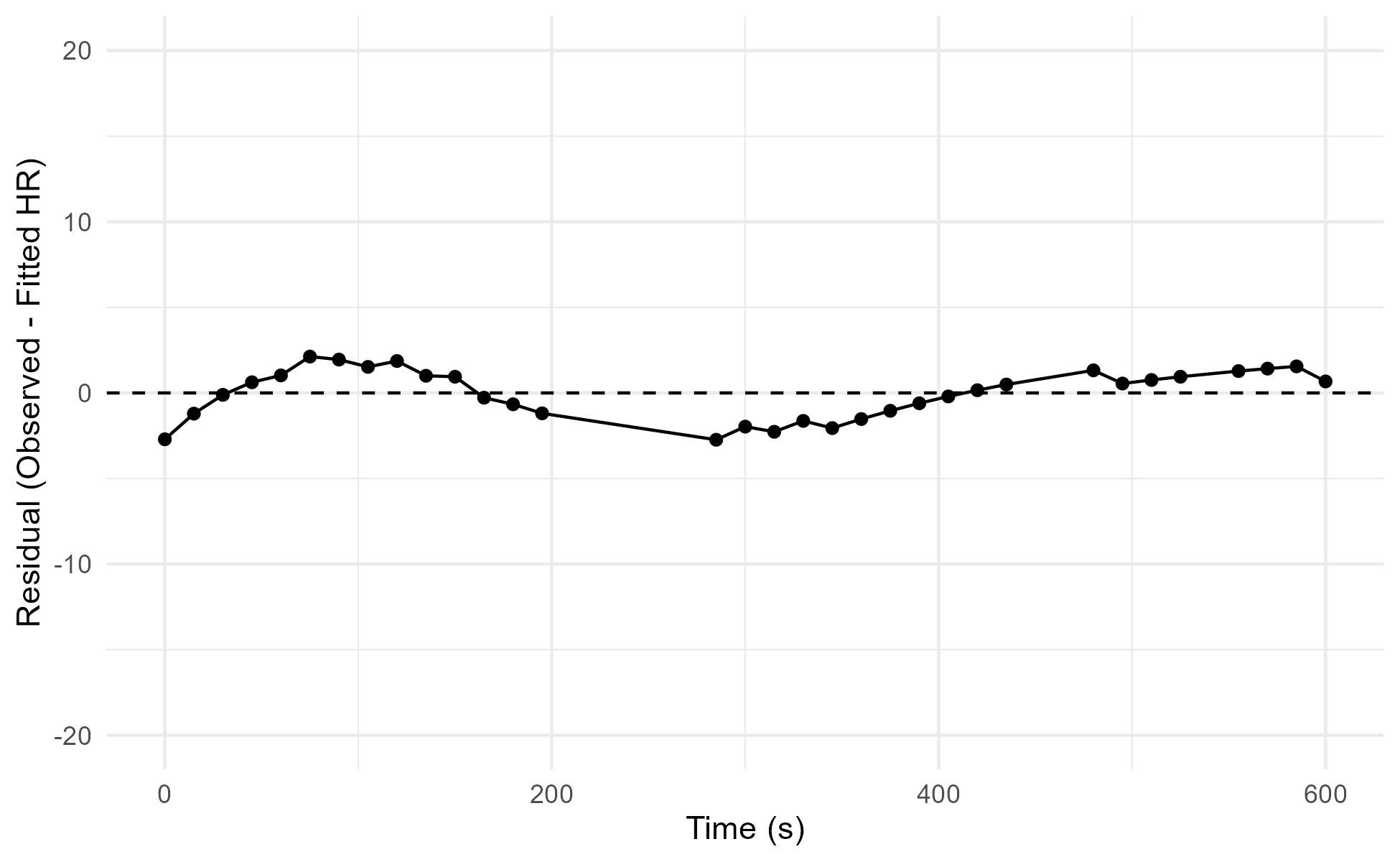


# Participant 14 – CVE trial

## Mono-exponential decay model fit


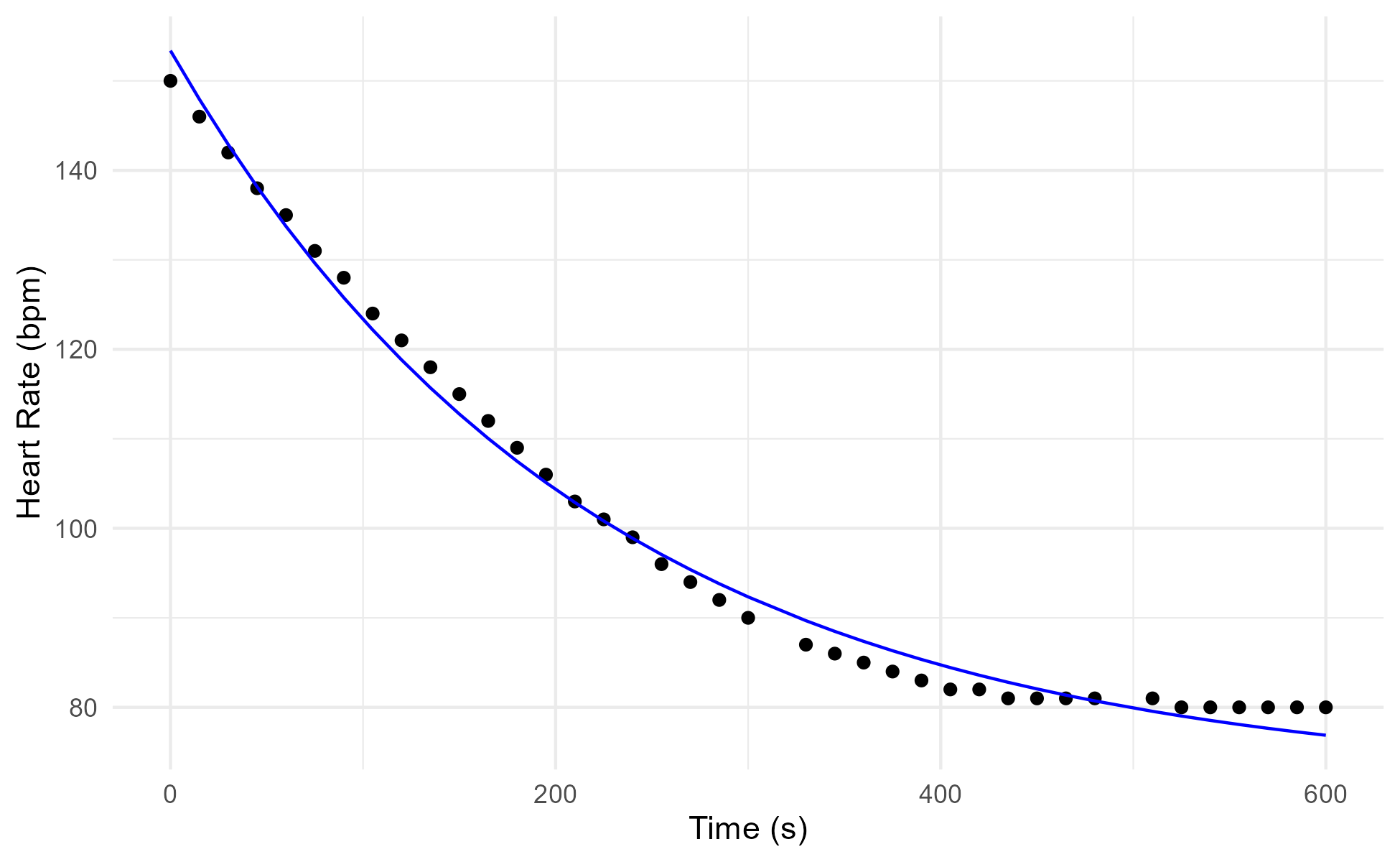


## Residuals of model fit


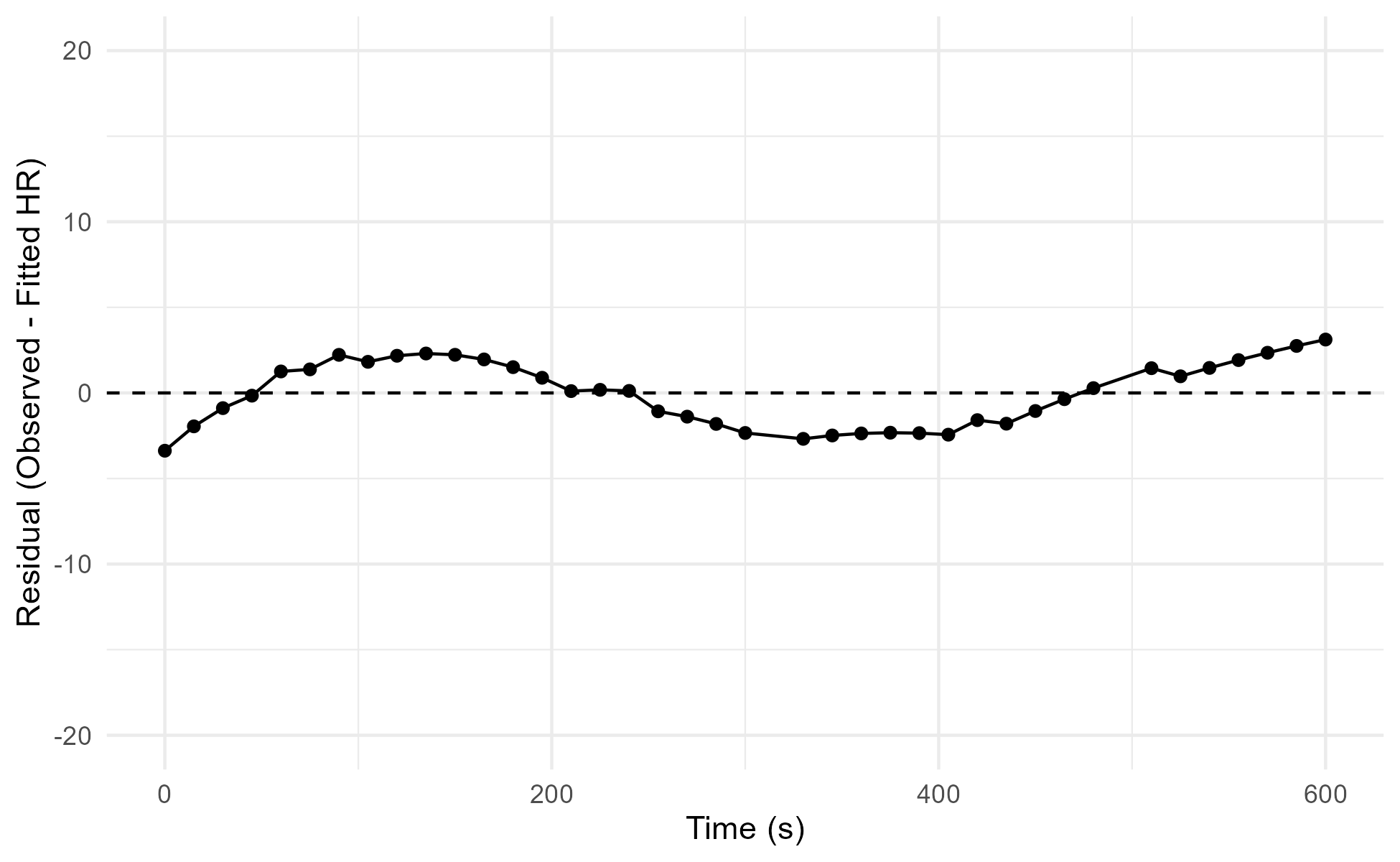


# Participant 15 – CME trial

## Mono-exponential decay model fit


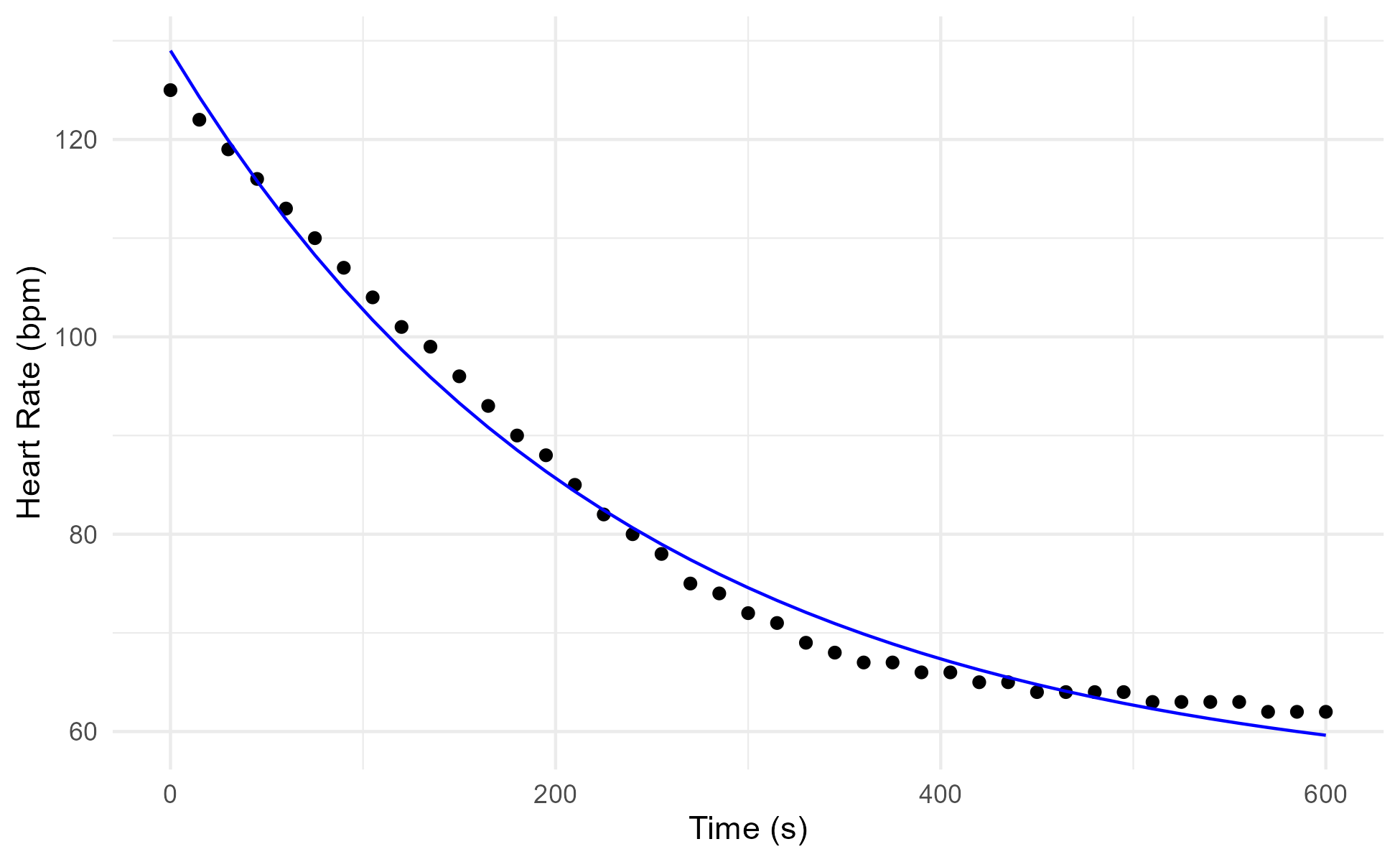


## Residuals of model fit


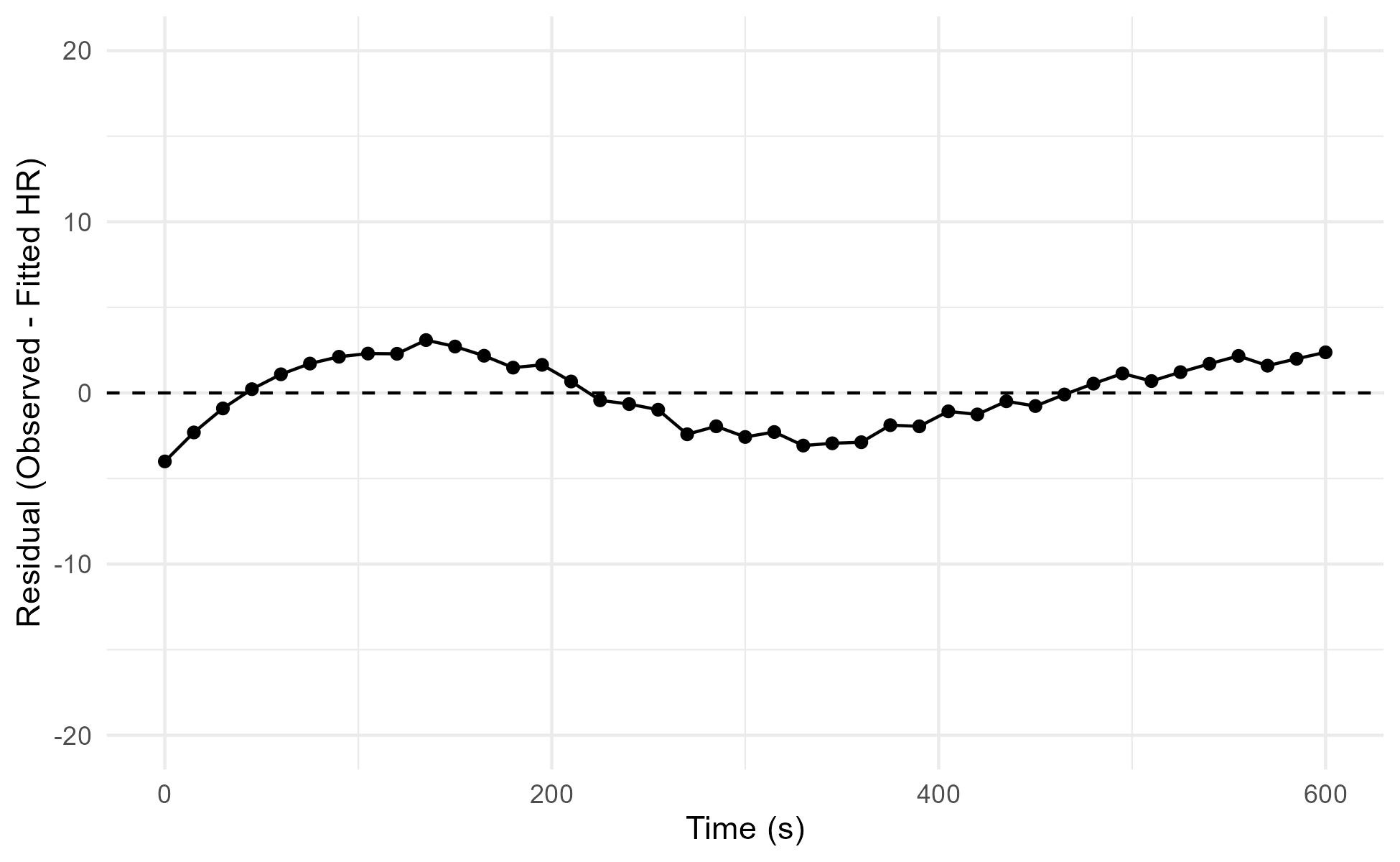


# Participant 15 – CVE trial

## Mono-exponential decay model fit


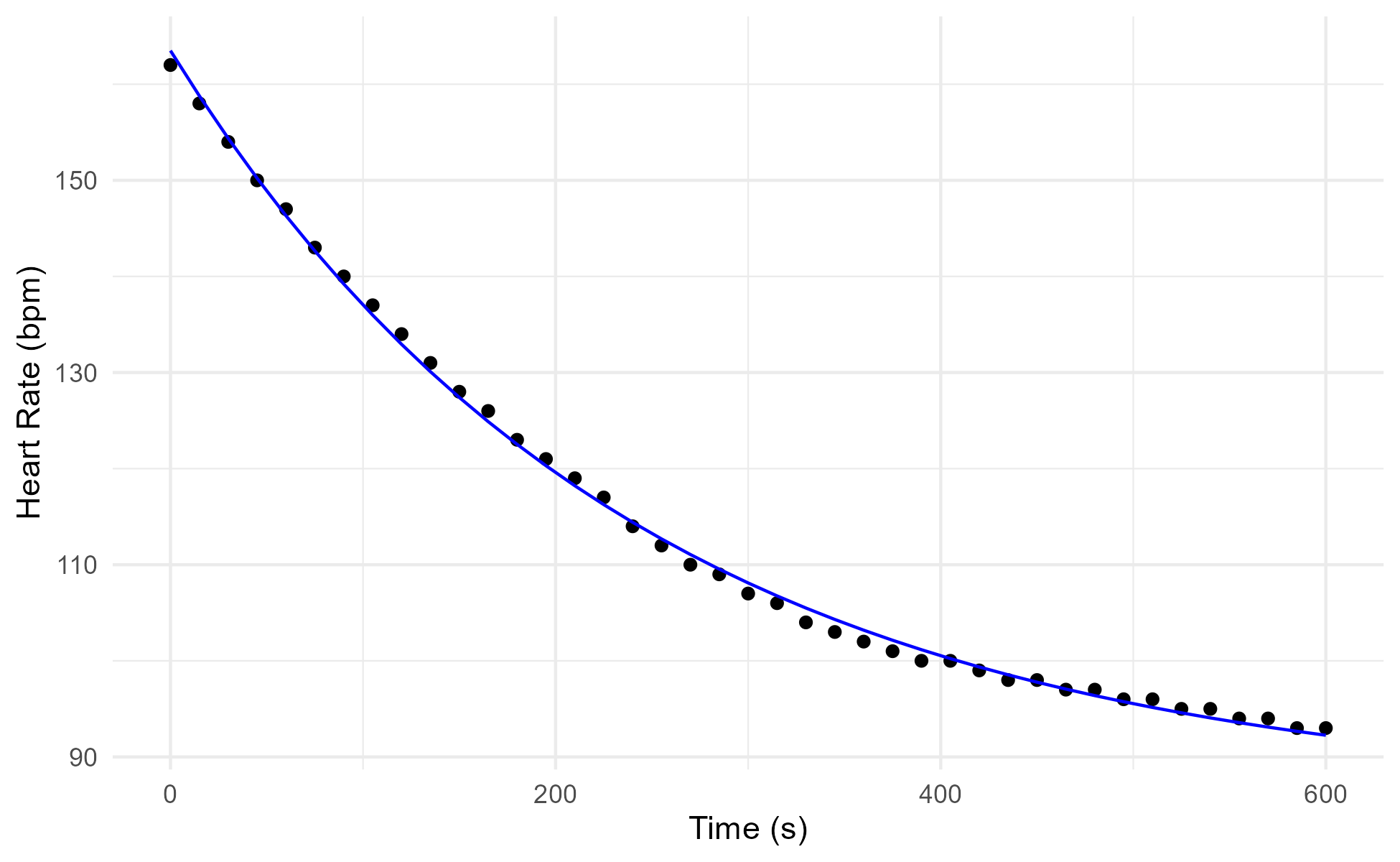


## Residuals of model fit


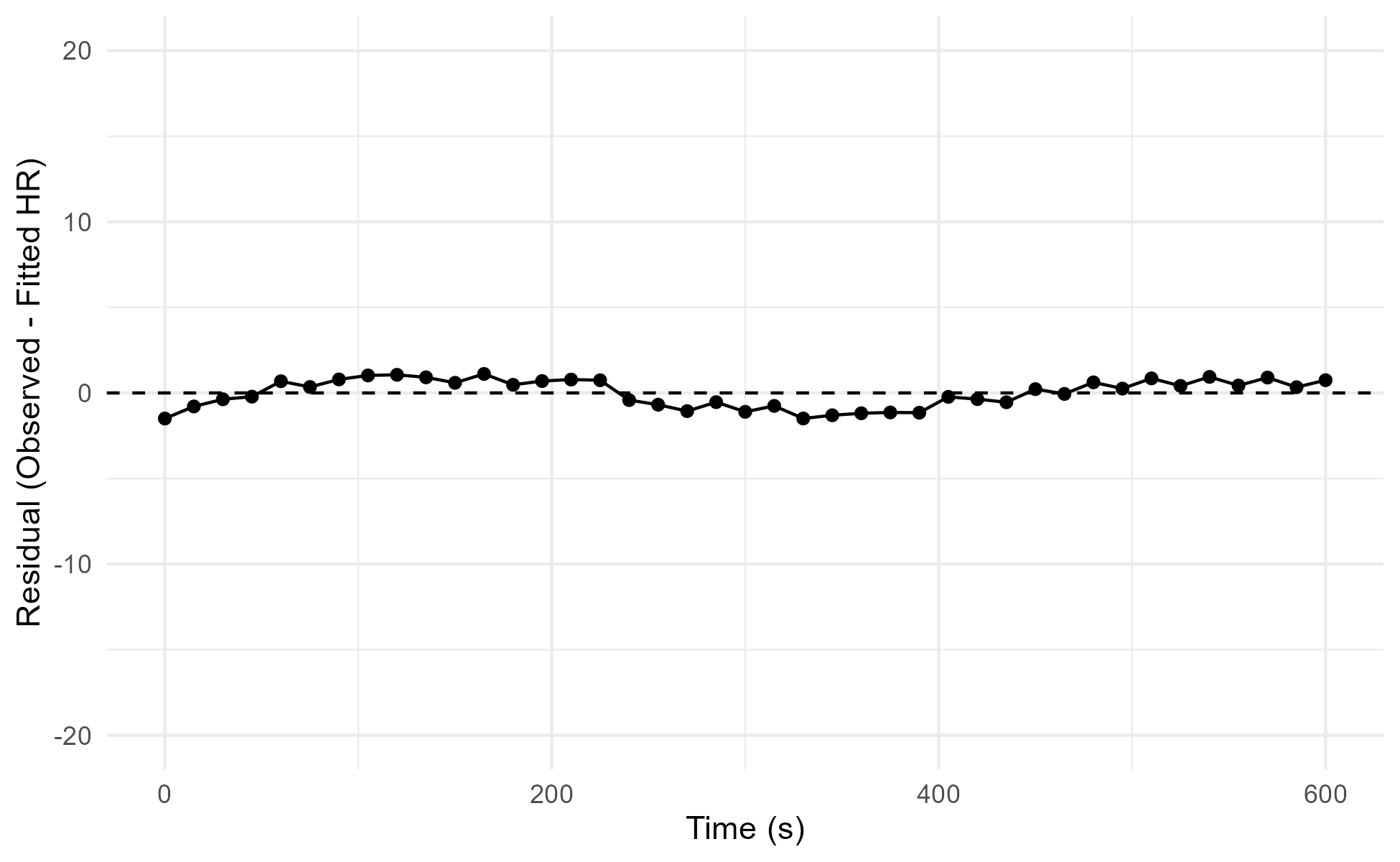


# Participant 16 – CME trial

## Mono-exponential decay model fit


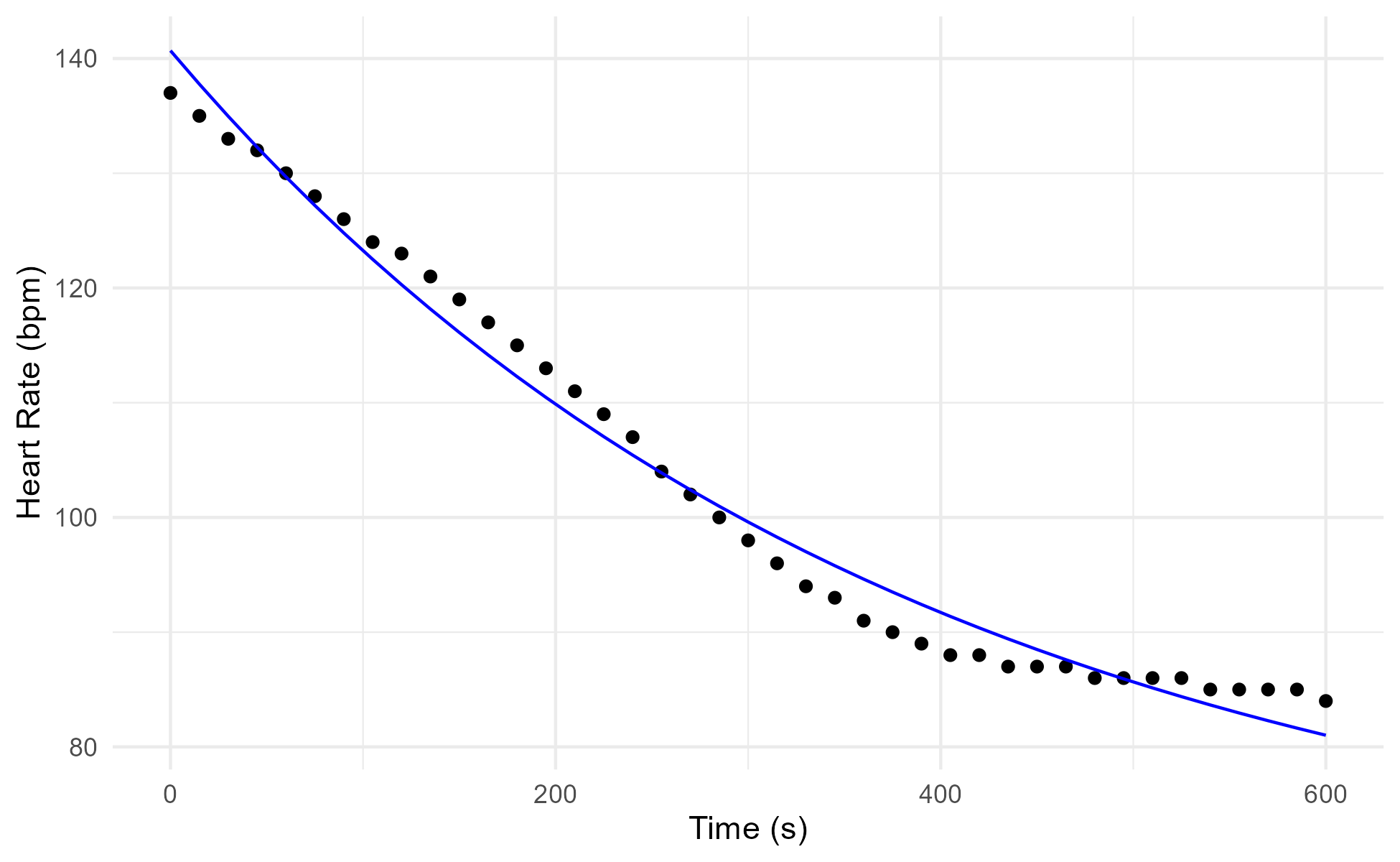


## Residuals of model fit


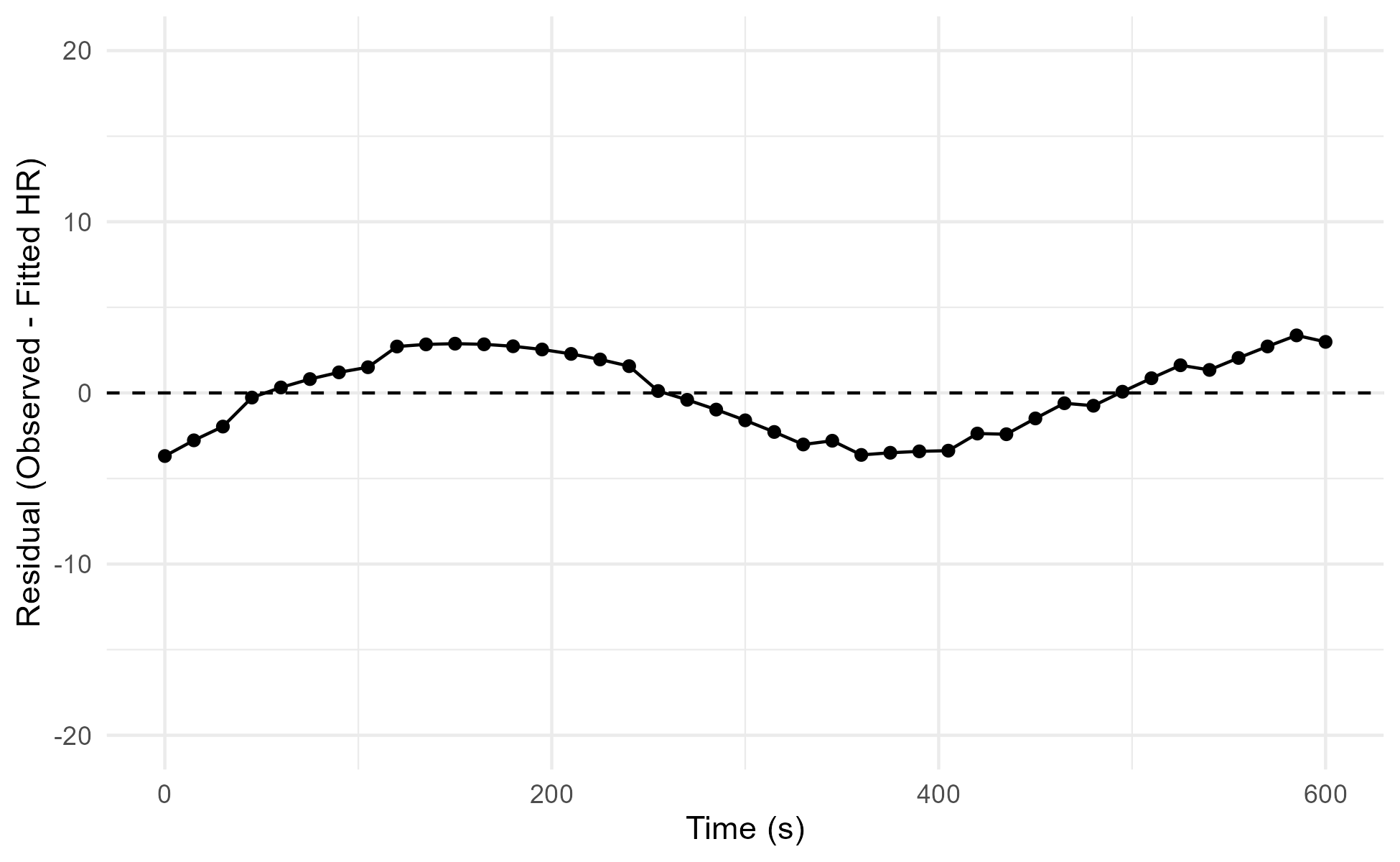


# Participant 16 – CVE trial

## Mono-exponential decay model fit


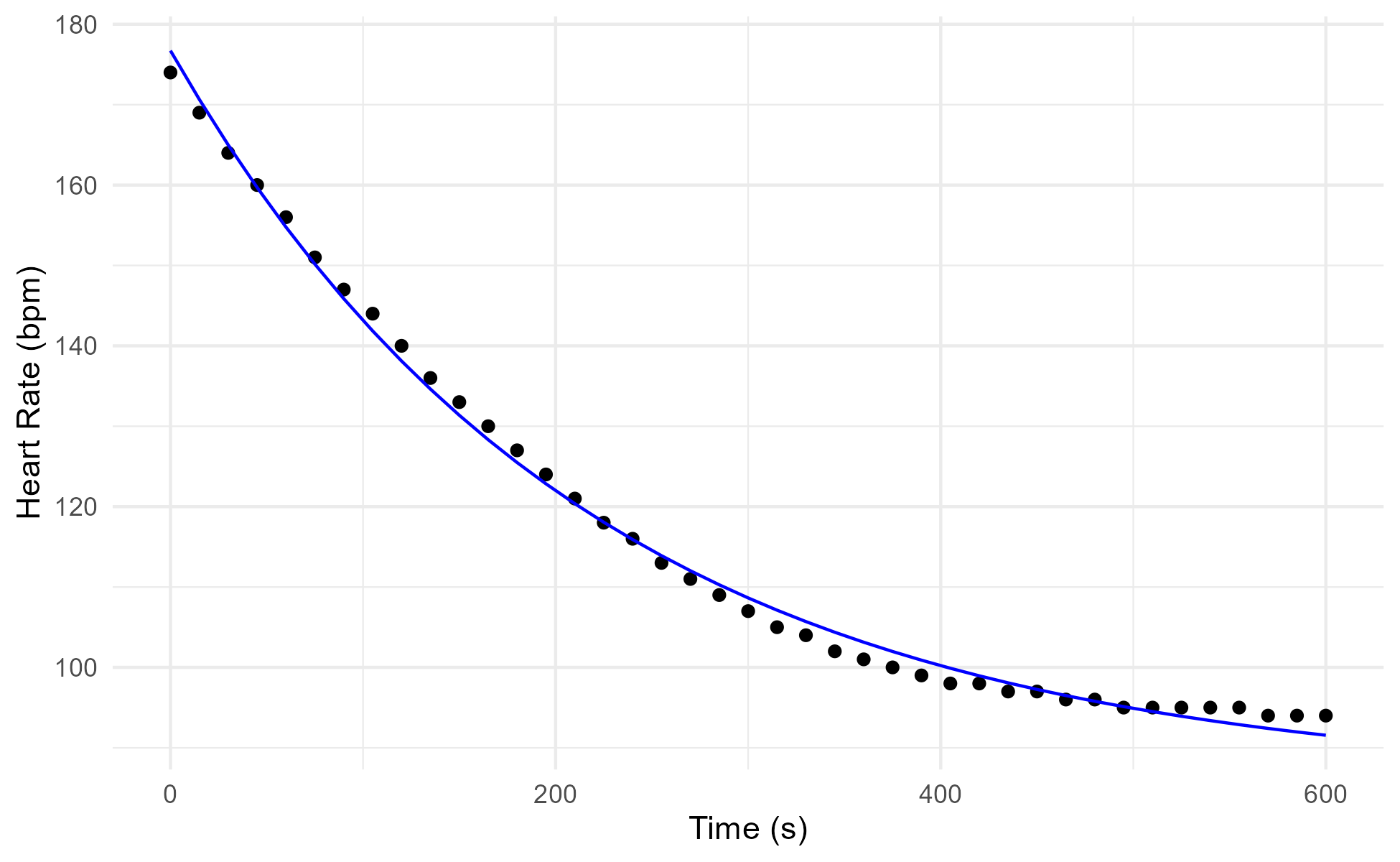


## Residuals of model fit


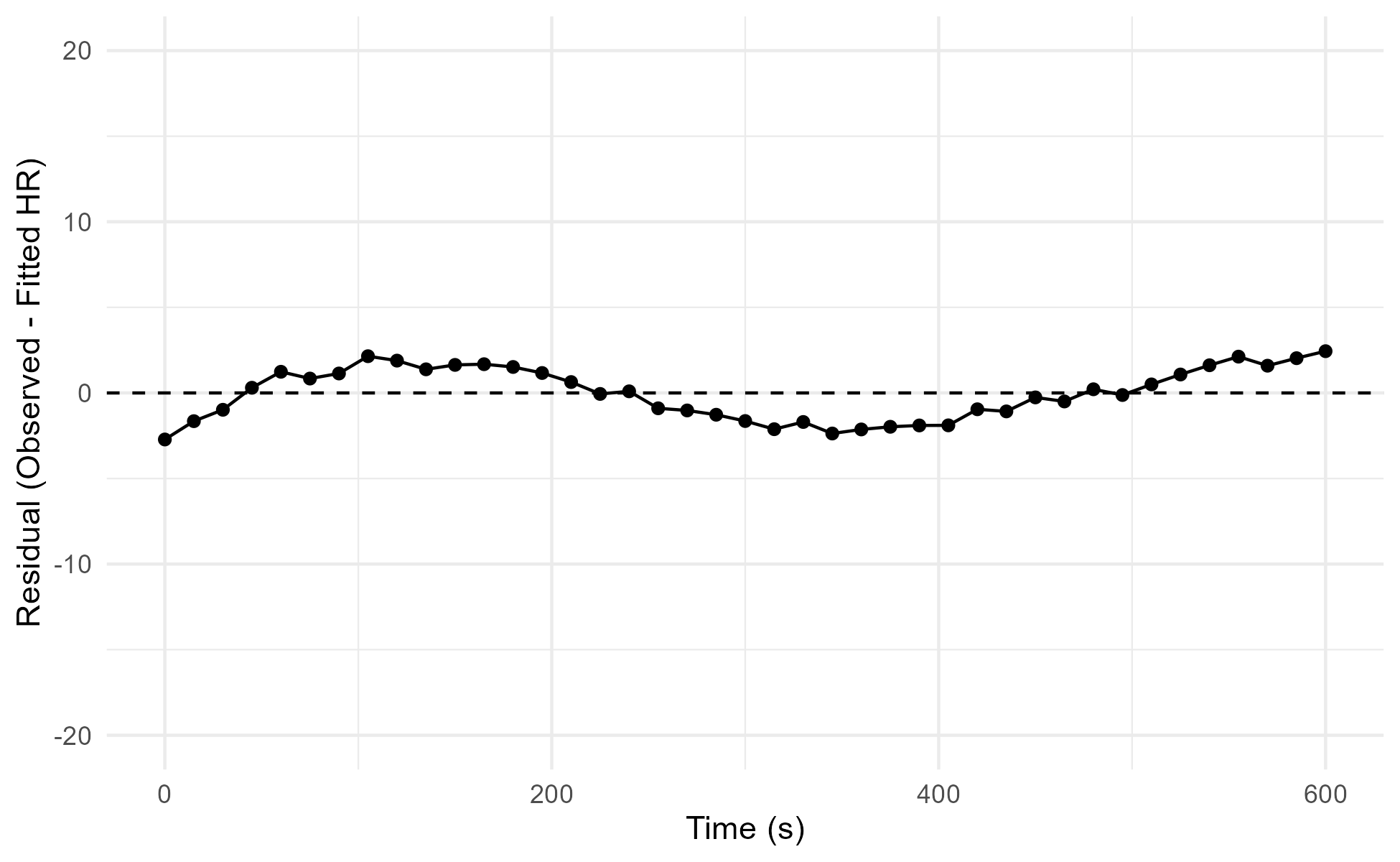


# Participant 17 – CME trial

## Mono-exponential decay model fit


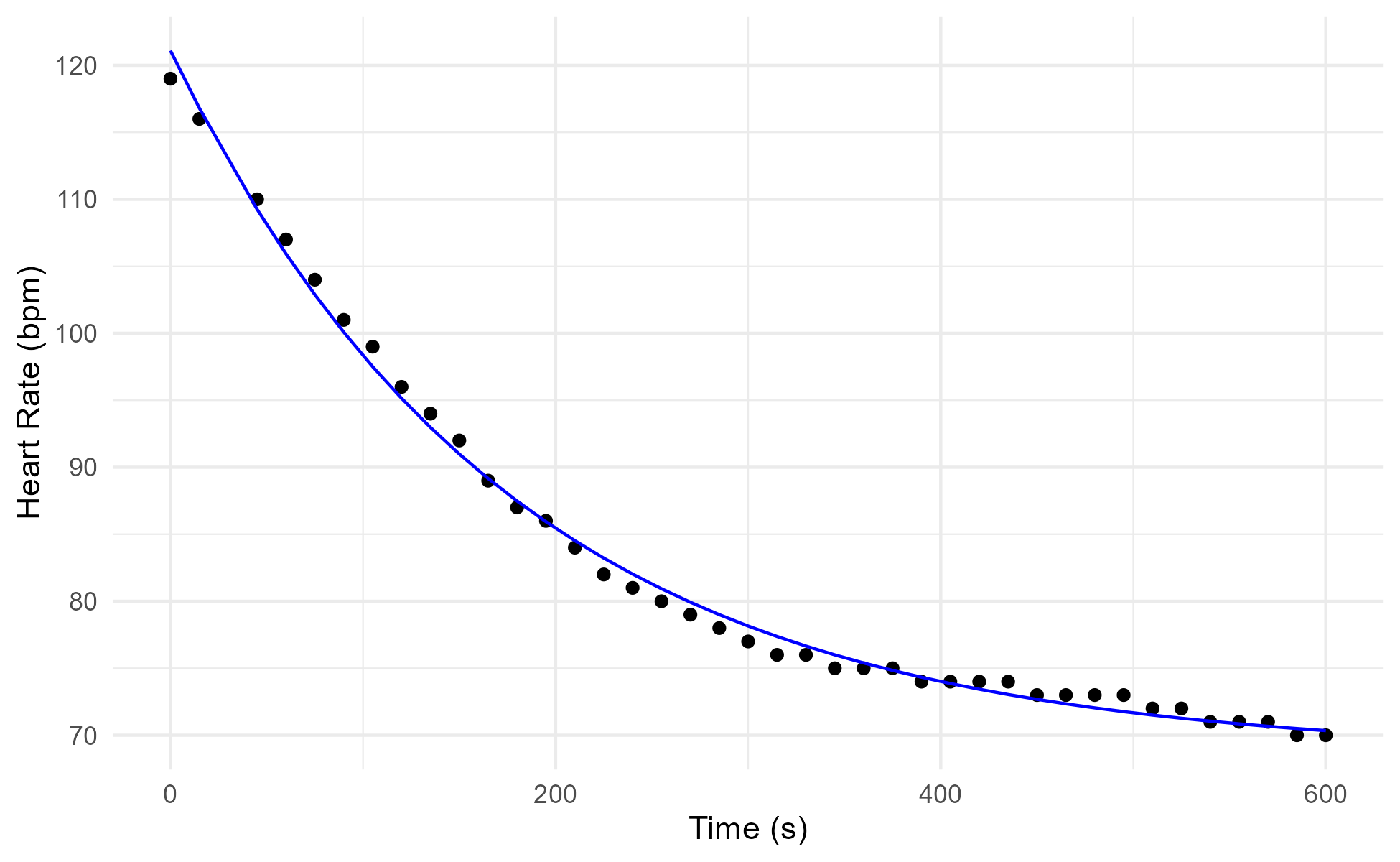


## Residuals of model fit


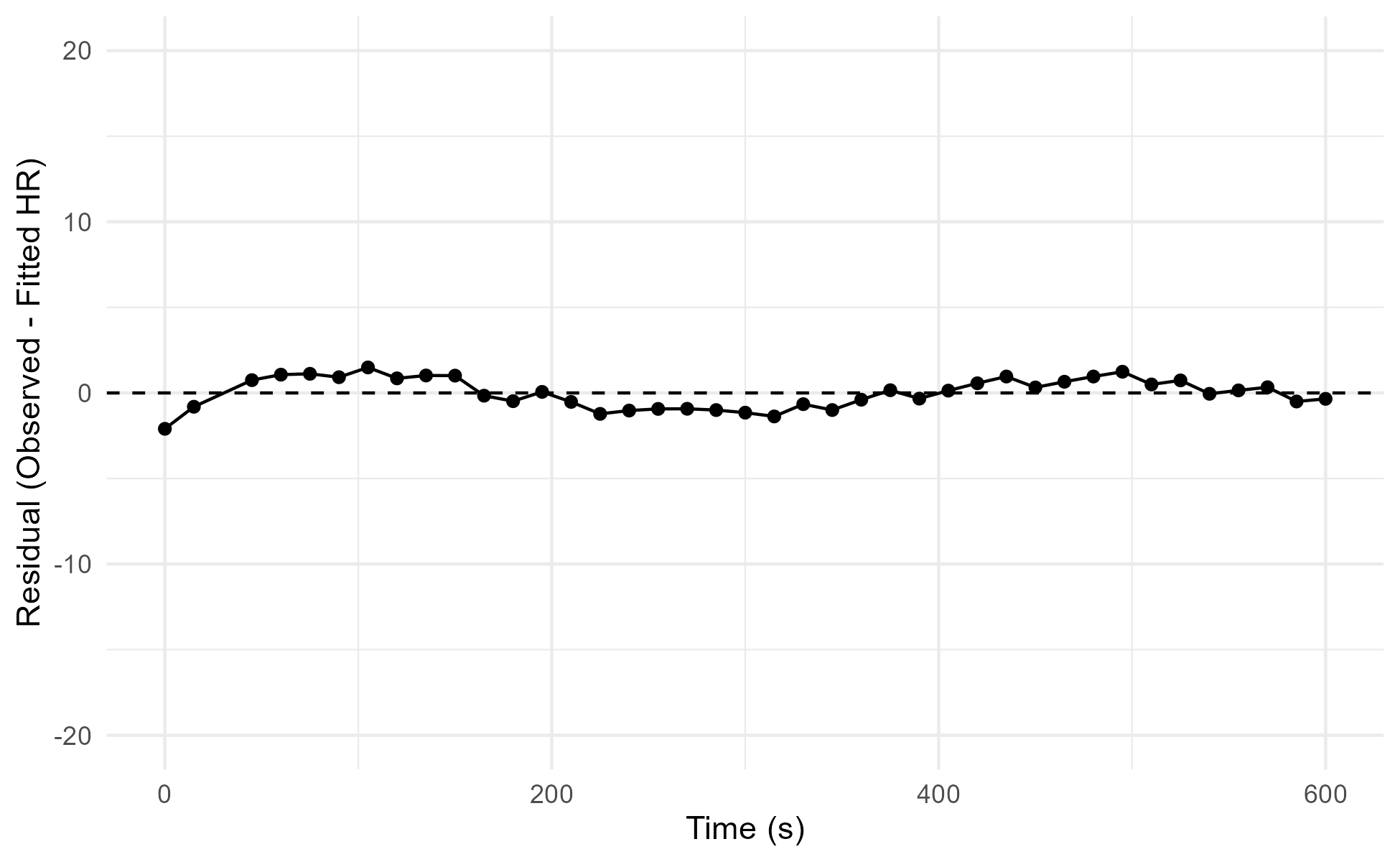


# Participant 17 – CVE trial

## Mono-exponential decay model fit


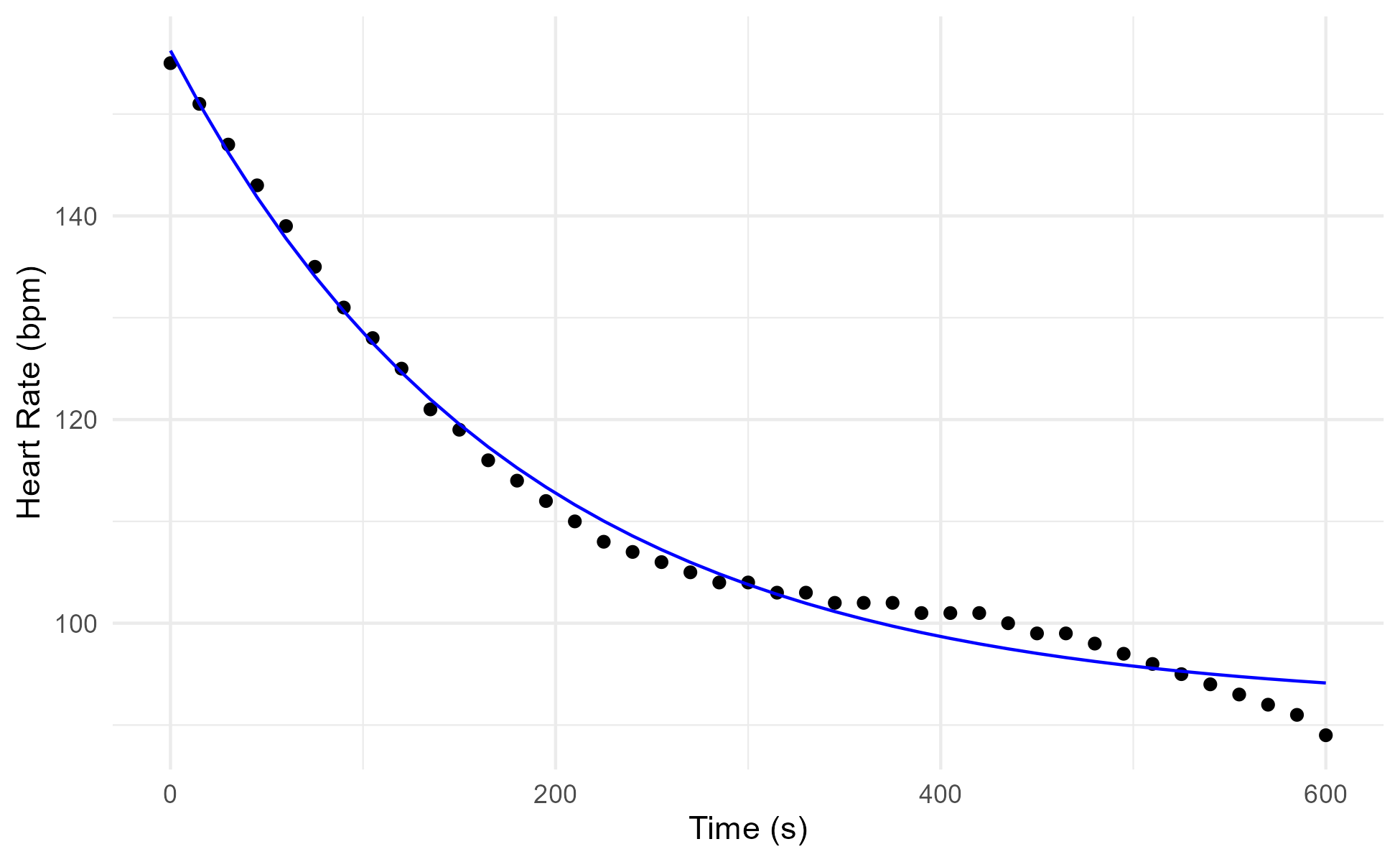


## Residuals of model fit


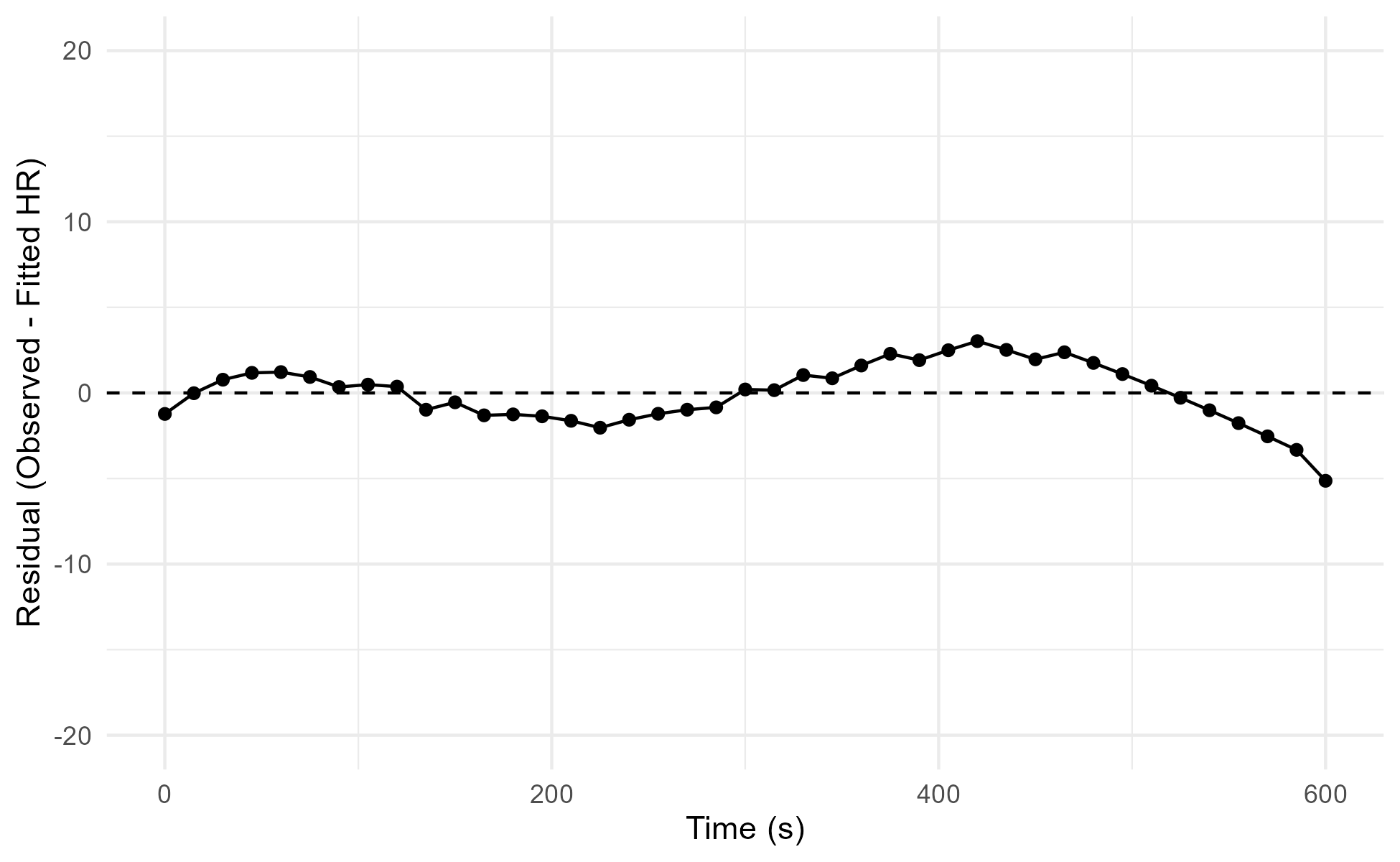

Supplement: Supplementary file 1 — Supplementary file1 (DOCX 3953 KB) [file 421_2026_6184_MOESM1_ESM.docx]
