# Supplementary material for: Heart rate recovery as a marker of post-exercise lipid metabolism following moderate- and vigorous-intensity exercise
Source: Eur J Appl Physiol. 2026 Mar 7;126(7):3747–61. doi: 10.1007/s00421-026-06184-y (PMC13380569; doi:10.1007/s00421-026-06184-y)
Supplement: Supplementary file 2 — Supplementary file2 (DOCX 659 KB) [file 421_2026_6184_MOESM2_ESM.docx]

**Supplementary Table 1**

**Heart rate recovery as a marker of** **post-exercise lipid metabolism following moderate- and vigorous-intensity exercise**

Dirk Weber^1^, Paola G. Ferrario^2^, Achim Bub^1,2^

^1^ Institute of Sports and Sports Science, Karlsruhe Institute of Technology, Karlsruhe, Germany,

^2^ Department of Physiology and Biochemistry of Nutrition, Max Rubner-Institute, Karlsruhe, Germany

*European Journal of Applied Physiology (Springer)*

**Corresponding author:**

Dirk Weber

Karlsruhe Institute of Technology (KIT)

Engler-Bunte-Ring 15

76131 Karlsruhe (Germany)

[dirk.weber@kit.edu](mailto:dirk.weber@kit.edu)

**Supplementary Table 1** Overview of inclusion and exclusion criteria for study participation

| **Inclusion Criteria** | **Exclusion Criteria** |
| --- | --- |
| - Healthy and physically active men - 18 years or older - Non-smokers - Provided written and informed consent | - Smokers - Volunteers with diseases of the gastrointestinal tract, metabolism, nervous system and infectious or immunological diseases in therapeutic need - Volunteers with tumors, acute or chronic infectious diseases - Volunteers with diseases of the cardiovascular system and/or cardiac pacemaker - Institutionalized patients in psychiatric hospitals - Volunteers with intolerances against gluten, fructose or lactose - Volunteers who donated blood in the last three months - Volunteers who may not adhere to the study protocol |

This supplementary material was previously published in:

Weber, D., Ferrario, P. G., & Bub, A. (2025). Exercise intensity determines circulating levels of Lac-Phe and other exerkines: A randomized crossover trial. *Metabolomics*, *21*(3), 63. https://doi.org/10.1007/s11306-025-02260-0
