# Supplementary material for: Heart rate recovery as a marker of post-exercise lipid metabolism following moderate- and vigorous-intensity exercise
Source: Eur J Appl Physiol. 2026 Mar 7;126(7):3747–61. doi: 10.1007/s00421-026-06184-y (PMC13380569; doi:10.1007/s00421-026-06184-y)
Supplement: Supplementary file 3 — Supplementary file3 (DOCX 665 KB) [file 421_2026_6184_MOESM3_ESM.docx]

**Supplementary Table 2**

**Heart rate recovery as a marker of** **post-exercise lipid metabolism following moderate- and vigorous-intensity exercise**

Dirk Weber^1^, Paola G. Ferrario^2^, Achim Bub^1,2^

^1^ Institute of Sports and Sports Science, Karlsruhe Institute of Technology, Karlsruhe, Germany,

^2^ Department of Physiology and Biochemistry of Nutrition, Max Rubner-Institute, Karlsruhe, Germany

*European Journal of Applied Physiology (Springer)*

**Corresponding author:**

Dirk Weber

Karlsruhe Institute of Technology (KIT)

Engler-Bunte-Ring 15

76131 Karlsruhe (Germany)

[dirk.weber@kit.edu](mailto:dirk.weber@kit.edu)

**Supplementary Table 2** Overview of calculated energy and macronutrient intake for (pre-)intervention days, actual energy and macronutrient intake at breakfast, and post-exercise water intake on both intervention days.

| ***Diet (pre-IV day and IV day)*** | ***Mean*** | **±** | ***SD*** | ***Min.*** | | ***Max.*** | |
| --- | --- | --- | --- | --- | --- | --- | --- |
| Energy intake (kcal)^1^ | 4102 | ± | 162 | 3770 | | 4324 | |
| Carbohydrates (%)^1^ | 55.0 | ± | 1.5 | 52.5 | | 57.4 | |
| Carbohydrates (g) ^1^ | 555.6 | ± | 23.2 | 522.6 | | 593 | |
| Fat (%)^1^ | 31.7 | ± | 1.6 | 29.1 | | 34.0 | |
| Fat (g)^1^ | 144.8 | ± | 9.7 | 124.8 | | 163.1 | |
| Proteins (%)^1^ | 10.9 | ± | 0.3 | 10.5 | | 11.4 | |
| Proteins (g)^1^ | 110.4 | ± | 6.6 | 97.2 | | 120.8 | |
| Fibers (%)^1^ | 2.3 | ± | 0.1 | 2.2 | | 2.4 | |
| Fibers (g)^1^ | 48.8 | ± | 2.4 | 44.9 | | 52.1 | |
| ***Breakfast (pre-CME trial)*** |  | | |  |  | |  |
| Energy intake (kcal)^2^ | 872 | ± | 195 | 456 | | 1428 | |
| Carbohydrates (%)^2^ | 63.9 | ± | 6.1* | 53.6 | | 77.6 | |
| Carbohydrates (g)^2^ | 137.7 | ± | 27.5 | 72.4 | | 197.2 | |
| Fat (%)^2^ | 25.0 | ± | 6.1 | 11.5 | | 35.9 | |
| Fat (g)^2^ | 25.1 | ± | 10.6 | 10.7 | | 57.7 | |
| Proteins (%)^2^ | 9.9 | ± | 2.5* | 6.9 | | 15.9 | |
| Proteins (g)^2^ | 21.4 | ± | 6.7* | 13.1 | | 37.1 | |
| Fibers (%)^2^ | 1.2 | ± | 0.1 | 0.9 | | 1.4 | |
| Fibers (g)^2^ | 5.3 | ± | 1.2 | 3.0 | | 7.9 | |
| ***Breakfast (pre-CVE trial)*** |  | | |  |  | | |
| Energy intake (kcal)^2^ | 981 | ± | 262 | 549 | | 1454 | |
| Carbohydrates (%)^2^ | 59.1 | ± | 7.4* | 44.4 | | 74.3 | |
| Carbohydrates (g)^2^ | 142.2 | ± | 35.6 | 91.2 | | 225.8 | |
| Fat (%)^2^ | 28.3 | ± | 6.4 | 13.3 | | 38.3 | |
| Fat (g)^2^ | 32.0 | ± | 12.5 | 11.3 | | 53.1 | |
| Proteins (%)^2^ | 11.5 | ± | 2.4* | 7.4 | | 16.3 | |
| Proteins (g)^2^ | 28.3 | ± | 10.7* | 13.0 | | 53.5 | |
| Fibers (%)^2^ | 1.1 | ± | 0.2 | 0.9 | | 1.5 | |
| Fibers (g)^2^ | 5.5 | ± | 1.4 | 4.0 | | 8.2 | |
| ***Liquid intake (post-exercise)*** |  |  |  |  | |  | |
| Water intake after CME trial (l) | 2.2 | ± | 0.6* | 1.3 | | 3.1 | |
| Water intake after CVE trial (l) | 2.7 | ± | 0.7* | 1.3 | | 4.0 | |

^1^: calculated energy/nutrient intake, i.e., amounts that should be consumed on pre-intervention and intervention days; ^2^: actual energy nutrient intake, i.e., amounts that were consumed at breakfast on intervention days, *: significant difference (*p* *<* 0.05) between CME and CVE trial based on paired Wilcoxon signed-rank test. %: percentage of energy intake from carbohydrates, fat, proteins, or fibers. CME: continuous moderate exercise; CVE: continuous vigorous exercise; IV: intervention; Max: maximum; Min: minimum; SD: standard deviation

This supplementary material was previously published in:

Weber, D., Ferrario, P. G., & Bub, A. (2025). Exercise intensity determines circulating levels of Lac-Phe and other exerkines: A randomized crossover trial. *Metabolomics*, *21*(3), 63. https://doi.org/10.1007/s11306-025-02260-0

Adapted from: Kistner, S. et al. Acute effects of moderate vs. vigorous endurance exercise on urinary metabolites in healthy, young, physically active men—A multi-platform metabolomics approach. Front. Physiol. 14, 1028643 (2023)
