# Supplementary material for: Heart rate recovery as a marker of post-exercise lipid metabolism following moderate- and vigorous-intensity exercise
Source: Eur J Appl Physiol. 2026 Mar 7;126(7):3747–61. doi: 10.1007/s00421-026-06184-y (PMC13380569; doi:10.1007/s00421-026-06184-y)
Supplement: Supplementary file 8 — Supplementary file8 (DOCX 18 KB) [file 421_2026_6184_MOESM8_ESM.docx]

**Supplementary Table 7**

Results of the linear mixed model analysis for different acylcarnitine subgroups at t4.

**Heart rate recovery as a marker of** **post-exercise lipid metabolism following moderate- and vigorous-intensity exercise**

Dirk Weber^1^, Paola G. Ferrario^2^, Achim Bub^1,2^

^1^ Institute of Sports and Sports Science, Karlsruhe Institute of Technology, Karlsruhe, Germany,

^2^ Department of Physiology and Biochemistry of Nutrition, Max Rubner-Institute, Karlsruhe, Germany

*European Journal of Applied Physiology (Springer)*

**Corresponding author:**

Dirk Weber

Karlsruhe Institute of Technology (KIT)

Engler-Bunte-Ring 15

76131 Karlsruhe (Germany)

[dirk.weber@kit.edu](mailto:dirk.weber@kit.edu)

# Linear Mixed Model Results for HRR:t4

| Group | Estimate | Standard Error | 95% CI | p-value | q-value |
| --- | --- | --- | --- | --- | --- |
| Short Chain | 0.064 | 0.027 | [0.012–0.117] | 0.017 | 0.019 |
| Medium Chain | 0.336 | 0.062 | [0.213–0.459] | < 0.001 | < 0.001 |
| Long Chain | 0.244 | 0.051 | [0.143–0.344] | < 0.001 | < 0.001 |
| Saturated | 0.156 | 0.035 | [0.087–0.225] | < 0.001 | < 0.001 |
| Monounsaturated | 0.278 | 0.054 | [0.170–0.386] | < 0.001 | < 0.001 |
| Polyunsaturated | 0.225 | 0.047 | [0.131–0.320] | < 0.001 | < 0.001 |
| Non-Hydroxy | 0.189 | 0.037 | [0.115–0.264] | < 0.001 | < 0.001 |
| Hydroxy | 0.278 | 0.069 | [0.140–0.415] | < 0.001 | < 0.001 |
| Lipid | 0.235 | 0.045 | [0.145–0.326] | < 0.001 | < 0.001 |
| Non-Lipid | 0.010 | 0.037 | [-0.063–0.082] | 0.793 | 0.793 |
